# Geography-guided industrial-level upcycling of polyethylene terephthalate plastics through alkaline seawater-based processes

**DOI:** 10.1126/sciadv.adu8381

**Published:** 2025-05-28

**Authors:** Zehao Xiao, Hongyu Guo, Fan Lv, Zheng Lin, Zongqiang Sun, Chenglong Sun, Yingjun Tan, Qizheng Huang, Mingchuan Luo, Shaojun Guo

**Affiliations:** School of Materials Science and Engineering, Peking University, Beijing, China.

## Abstract

The escalating plastic crisis can be mitigated by upgrading waste polyethylene terephthalate (PET). Leveraging the geographical advantages of offshores with established chlor-alkali industries, abundant renewable energy, and extensive seawater, we here present a technically and economically viable strategy of harnessing natural seawater as a medium to transform PET plastics into high-value chemicals. We report a nickel-molybdenum catalyst incorporating frustrated Lewis pairs for the efficient breakage of C─C bond and the oxidation of ethylene glycol, which sustains a current of 6 amperes at 1.74 volts over 350 hours, with a projected revenue of approximately $304 United States dollar (USD) per ton of processed PET plastics. In a customized electrolyzer, we successfully convert 301.0 grams of waste PET into 227.1 grams of p-phthalic acid (95.5% yield), 1486.2 grams of potassium diformate (67.2% yield), and approximately 214.9 liters of green hydrogen. This study paves the way for scalable PET upcycling, contributing to a circular economy and mitigating the plastic pollution crisis.

## Teaser

Geography-guided integration of alkaline seawater hydrolysis and electrolysis upgrades waste PET plastics at an industrial rate.

## INTRODUCTION

**Fig. 1.**
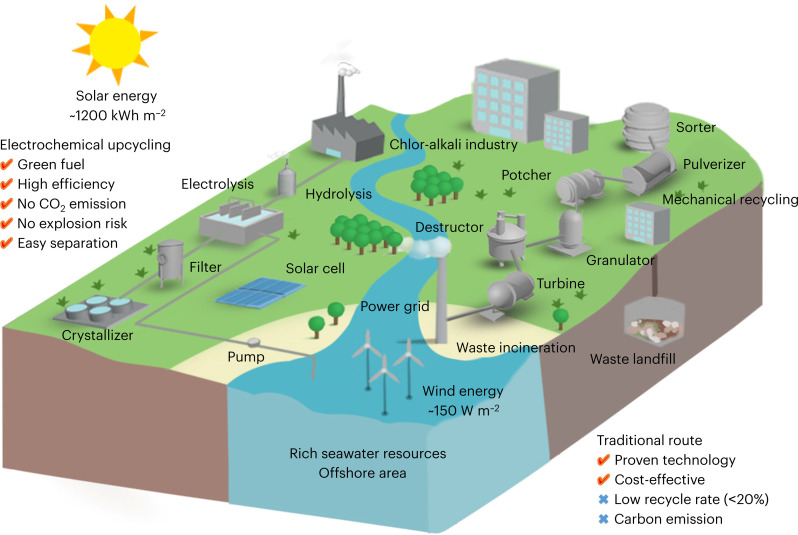
Technical route analysis. Schematic illustration of mechanical PET recycling, waste incineration, waste landfill, and electrochemical PET upcycling routes.

Over the past century, polyethylene terephthalate (PET) has emerged as a pivotal engineering plastic, witnessing massive production and widespread use in packaging, films, fibers, and containers. With an annual production rate of approximately 70 million tons, enhancing the recycling efficiency of PET plastics remains a pressing challenge (*1*, *2*). To reverse this trend, it is imperative to explore innovative techniques capable of converting PET waste into value-added products (*3*, *4*). However, conventional methods, such as mechanical recycling, incineration, and landfilling, face limitations in terms of low recycling efficiency, with substantial carbon footprint (Fig. 1) (*5*, *6*). An emerging and green strategy levers the alkaline depolymerization of PET into terephthalate and ethylene glycol (EG) (*7*), the former can be readily collected via pH-dependent precipitation. However, the latter EG is difficult to be separated because of its high solubility and boiling point (*8*, *9*), which urges a low-cost and eco-friendly route for upgrading EG from the hydrolysate. Recently, Hyeon and co-workers (*10*) reported a PET photoreforming strategy, and EG under this scheme is selectively oxidized by photogenerated holes into CO_2_; Ma and co-workers (*11*) reported a controlled hydrogenation thermocatalytic approach to transform PET into a degradable polyester. Despite the well-studied demonstration, there are still challenges of poor PET conversion efficiency and harsh reaction condition toward value-added products.

A promising strategy for alkaline PET hydrolysate electrolysis under mild condition involves generating cathodic green hydrogen while simultaneously converting anodic EG into value-added chemicals, all without emitting CO_2_. This approach leverages the alkaline chemistry to facilitate both PET hydrolysis and EG oxidation reaction (EGOR), which operates at a lower thermodynamic equilibrium potential (fig. S1) (*7*). While recent advancements have demonstrated the potential of cost-effective nonnoble metal catalysts (e.g., Co-Ni_3_N, CoNi_0.25_P, Co-NiS, and Ni_1_Mn_1_-MOF) for electrochemical PET upcycling (*12*–*15*), several challenges still remain for large-scale industrial implementation. One major challenge is ensuring a steady supply of massive water for hydrolysis, particularly in regions with water scarcity, such as northwest China, which accesses abundant renewable electricity but faces limitations due to low rainfall and long-distance water transportation (~5.7% of the country’s water reserve) (*16*, *17*). In contrast, offshore areas offer abundant seawater resources (more than 96.5% of global water storage), which can serve as a sustainable source of water for electrolysis while also harnessing offshore wind (100 to 200 W m^−2^) and solar energy (1200 to 1500 kilowatt-hour m^−2^) (*14*). Furthermore, these regions benefit from established chlor-alkali industries derived from seawater, providing a ready supply of KOH/NaOH for the process (Fig. 1) (*17*, *18*). Another critical aspect is the durability of the catalyst and system. Critical ampere-level current stability tests on electrochemical PET upcycling report are always ignored, leaving questions about their long-term performance under industrial-scale demands (*19*, *20*). Therefore, developing cost-effective catalysts that can sustainably convert PET hydrolysate into easily separable products under industrial operating conditions is essential. By leveraging the geographical advantages of offshore areas and focusing on catalyst optimization, we can pave the way for a more sustainable and efficient approach to PET upcycling.

In this work, we present an innovative and economically viable strategy for the successive hydrolysis and electrolysis of waste PET in alkaline natural seawater. This approach leverages the abundant seawater resources and the established chlor-alkali industry to furnish the necessary raw materials for PET hydrolysis while harnessing renewable energy sources to power the concurrent electrolysis process. To further enhance the efficiency of this process, we introduce Mo doping as an active auxiliary component into Ni hydroxides, thereby creating frustrated Lewis pairs (FLPs). These FLPs effectively combine spatially incompatible electron acceptors with donors, fostering charge redistribution and optimizing the adsorption energy required for the efficient conversion of EG to formate (figs. S2 and S3) (*21*–*23*). Contrary to universal strategies that often guarantee a stable income, the economic model underpinning electrochemical PET upcycling demonstrates a nuanced relationship between revenue and the applied voltage and current (fig. S4). However, the cost of widely used titanium alloy–made membrane electrode assembly will markedly increase as the catalyst amplification for higher current, and this equipment is prone to be severely corroded under heating alkaline solution. Hence, we used low-cost and stable polymeric methyl methacrylate to design the advanced slot-type parallel flow electrolyzer, which could drive a record current of 6 A under 1.74 V for more than 350 hours. Our integrated approach successfully upcycled 301.0 g of PET into approximately 214.9 liters of hydrogen gas, along with 227.1 g of p-phthalic acid (PTA; 95.5% yield) and 1486.2 g of potassium diformate (KDF; 67.2% yield), through the continuous operation of evaporation, crystallization, and filtration. This transformation generated a substantial revenue of approximately $304 USD per ton of processed PET. We further unravel the underlying mechanisms, revealing the stability of active sites facilitated by the spontaneous reduction of EG, and the pivotal role played by FLPs in accelerating C─C bond cleavage. This proof-of-concept demonstration not only offers a promising solution to alleviate the pressing plastic crisis but also establishes a benchmark for efficient PET degradation methods, achieving the highest efficiency with the lowest CO_2_ emissions among existing approaches.

## RESULTS

### Catalyst design and characterizations

**Fig. 2.**
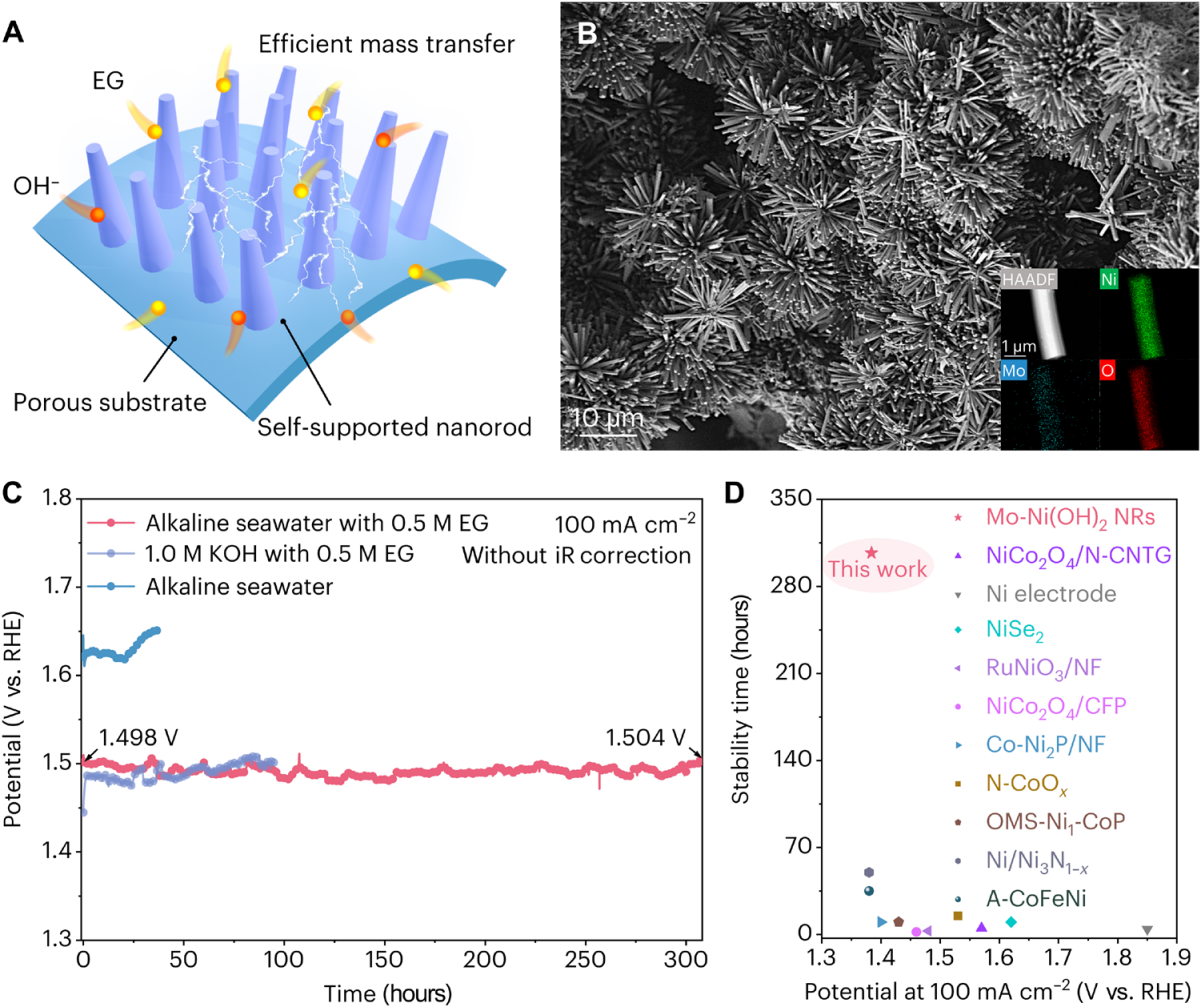
Characterizations and catalytic activities of catalysts. (**A**) Schematic illustration of the Mo-Ni(OH)_2_ electrode. (**B**) SEM image of Mo-Ni(OH)_2_. Inset shows the energy-dispersive spectroscopy elemental mapping images of Mo-Ni(OH)_2_. (**C**) Chronopotentiometry (CP) test in 1.0 M KOH with 0.5 M EG and alkaline seawater with/without 0.5 M EG. (**D**) Comparison of EGOR activity with recently reported non-noble metal catalysts. HAADF, high angle angular dark field.

We initially screened the potential cost-effective and sustainable catalysts for EGOR and investigated the effect of pure water/seawater media on their catalytic activity. Given molybdate as a class of self-supported precursors, we proposed the continuous reconstruction of NiMoO_4_·*x*H_2_O on nickel foam (NF) via in situ electrooxidation coupled with MoO_4_^2−^ ions leaching, followed by the spontaneous chemical reduction of EG to Mo-Ni(OH)_2_ nanorods during EGOR (figs. S5 and S6). The scanning electron microscope (SEM) image shows that the densely interconnected nanorods are beneficial to mass and charge transfer (Fig. 2, A and B, and figs. S7 to S10). The transmission electron microscope (TEM) image with the selected-area electron diffraction reveals typical lattice mismatching of the amorphous phase (fig. S11) (*24*). The x-ray diffraction (XRD) pattern (fig. S12) indicates that the NiMoO_4_·*x*H_2_O precursor (PDF #12-0348) transforms into nickel hydroxide phase (PDF #14-0117).

As a control, we synthesized Ni(OH)_2_ nanosheets (NSs) with similar mass loading (figs. S13 and S14) (*25*). The Ni 2p spectra indicate that Mo-Ni(OH)_2_ has a lower ratio of Ni^3+^/Ni^2+^ (fig. S15) (*26*, *27*). Decreasing signal in Mo 3d spectra fits with the inductively coupled plasma optical emission spectrometry (ICP-OES) result that large amounts of MoO_4_^2−^ ions dissolve from the catalyst during the electrooxidation process (table S1 and fig. S16). The disappearance of the Ni^3+^-O peak in the O 1s spectra verifies the conversion of hydroxides (fig. S17) (*28*). The negative shift of Ni *K*-edge x-ray absorption near edge structure spectra of Mo-Ni(OH)_2_ represents a lower average valence state (figs. S18 to S20). Fourier transform extended x-ray absorption fine structure (EXAFS) spectra in *R*-space show intensity maximum of Ni-Ni scattering at 2.48 Å and Ni-O coordination at 2.08 Å (table S2), confirming that the spontaneous chemical reduction of EG results in the decreased oxide-modified nickel state (*29*).

### Electrocatalysis of EG oxidation

Half-cell EGOR performances under alkaline water/seawater media were evaluated through a three-electrode system. As shown in figs. S21 and S22, linear sweep voltammetry (LSV) curves that exhibit Mo-Ni(OH)_2_ only requires 1.38 and 1.41 V versus the reversible hydrogen electrode (RHE) to drive 100 and 300 mA cm^−2^. Notably, the reducing EGOR activity as more MoO_4_^2−^ ions leaching means that Mo sites are critical to boosting intrinsic activity (figs. S23 and S24). Furthermore, LSV curve in 1.0 M KOH as the control demands higher potentials of 1.52 and 1.59 V versus RHE to drive 100 and 300 mA cm^−2^ (figs. S25 and S26). The lowest Tafel slope (37.0 mV dec^−1^) of Mo-Ni(OH)_2_ represents the fastest reaction kinetics (fig. S27). The comparably excellent EGOR activity of our designed Mo-Ni(OH)_2_ catalyst under alkaline water/seawater media indicates the feasibility of natural seawater feed to the electrochemical PET upcycling strategy. ^1^H nuclear magnetic resonance (NMR) spectroscopy shows that the peak located at 3.5 parts per million (ppm) of EG weakens as EGOR proceeds, while the peak located at 8.3 ppm of formate increases, and no obvious peak belonging to incomplete oxidation product of glycolate at 3.8 ppm is observed (fig. S28). The high-performance liquid chromatography (HPLC) chromatograms demonstrate that the highest faradic efficiency (FE; 95.8%) with selectivity (97.7%) is obtained at 1.55 V versus RHE (figs. S29 to S32).

The Mo-Ni(OH)_2_ shows an impressive stability with negligible deactivation over 300 hours under a current density of 100 mA cm^−2^ in a flow electrolyzer (Fig. 2C and figs. S33 and S34). The activity of Mo-Ni(OH)_2_ surpasses most of existing non-noble metal catalysts (Fig. 2D and table S3). We also synthesized other FLP-based catalysts (e.g., Mo-Co and W-Ni) using our approach and demonstrated enhanced activity toward the methanol/ethanol oxidation reaction (figs. S35 to S38).

Furthermore, a lower zeta potential of Mo-Ni(OH)_2_ (−6.24 mV) than that of Ni(OH)_2_ (−0.79 mV) implies better chlorine resistance of the former (fig. S39). As shown in figs. S40 and S41, increasing double-layer capacitance (*C*_dl_) of Mo-Ni(OH)_2_ for EGOR implies the larger electrochemical active surface area (ECSA). Both ECSA and mass loading–normalized LSV curves (fig. S42) indicate that Mo-Ni(OH)_2_ still has higher turnover frequency (fig. S43) and intrinsic catalytic performance. The Mo-Ni(OH)_2_ catalyst shows high catalytic stability for EGOR, as confirmed by the retained nanorod structure in the SEM result (fig. S44), the nearly unchanged ratio of Ni^2+^/Ni^3+^ in Ni 2p x-ray photoelectron spectroscopy (XPS) spectra (fig. S45), and also the almost unchanged lattice fringe of 0.233 nm assigned to the (101) plane for Ni(OH)_2_ in the XRD and TEM results (figs. S46 and S47) after long-term EGOR.

### Mechanism studies of EGOR

According to the above analysis, we proposed a series of mechanism analysis toward EGOR. The intermittent analysis was used to study the dehydrogenation of EG and proton deintercalation under the absence of applied potential. As shown in fig. S48, when an oxidation potential of 1.35 V versus RHE was applied in 1.0 M KOH, oxidation current occurred along with the proton removal of hydroxides to oxyhydroxides (M-OH → M-OOH). Afterward, 0.1 M EG was added, while the potential was maintained at open circuit potential (OCP). As the potential of 1.0 V versus RHE was applied, the reduction current was decreased after adding EG, indicating that the generated oxyhydroxides accept protons from EG to hydroxides.

LSV curves under different KOH and EG solutions were measured to study reaction orders and coadsorptions of OH^−^ and EG. As shown in fig. S49, the EGOR exhibits strong dependence with OH^−^, which is consistent with the mechanism proposed by Fleischmann *et al*. (*30*) that alkaline alcohol oxidation requires necessary alcohol preoxidation to aldehyde [RCH_2_OH_ads_ + NiOOH → RCHOH_ads_ + Ni(OH)_2_]. The higher Nernstian shift of pH with onset potential for Mo-Ni(OH)_2_ was calculated as 75.5 mV pH^−1^, which indicates that the FLPs influence the proton surface coverage (fig. S50) (*31*). The linearity of current densities with increasing concentrations of EG shows a turning point, which represents the competitive adsorption relationship between EG and OH^−^ over the active sites (fig. S51).

**Fig. 3.**
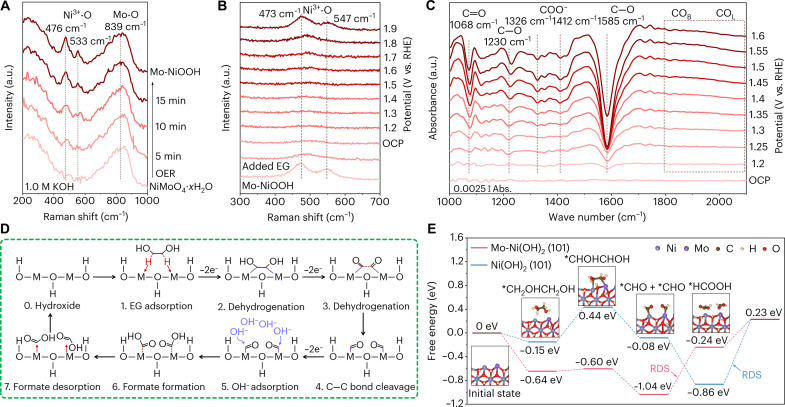
Insights of the EGOR mechanism. (**A**) In situ Raman spectra of Mo-NiOOH collected in 1.0 M KOH. (**B**) In situ Raman spectra of Mo-Ni(OH)_2_ collected in 1.0 M KOH with 0.1 M EG. (**C**) In situ FTIR spectra of Mo-Ni(OH)_2_ collected in 1.0 M KOH with 0.1 M EG. (**D**) Schematic representation of the EGOR mechanism. (**E**) Gibbs free energy of EGOR stepwise pathway for Mo-Ni(OH)_2_ (101) and Ni(OH)_2_ (101). a.u., arbitrary units. abs., absorbance.

Operando electrochemical impedance spectra (EIS) were used to observe the properties of the reaction at the catalyst/electrolyte interface. As for oxygen evolution reaction (OER), characteristic peaks at the low-frequency region (0 to 1 Hz) below 1.35 V versus RHE relate to the oxidation of Ni^2+^-OH to Ni^3+^-O (fig. S52) (*32*). As for EGOR, characteristic peaks decrease at the middle-frequency region (1 to 100 Hz) over 1.20 V versus RHE, which confirms that the chemical reduction rate of EG is higher than the self-oxidation rate (fig. S53). Therefore, we conclude that EGOR directly proceeds at the interface between surface hydroxide layers instead of oxidized layers (e.g., NiOOH and NiO*_x_*). Moreover, the lower impedance values of Mo-Ni(OH)_2_ obtained by fitting Bode plots through the equivalent circuit indicate that the introduction of Mo is essential to improve the conductivity (fig. S54). In the Raman spectra of Mo-NiOOH, two strong doublet peaks at 476 and 553 cm^−1^ belong to the Ni^3+^─O stretching vibration (Fig. 3, A and B) (*33*). At potentials below 1.7 V versus RHE, the absence of NiOOH characteristic peaks further indicates that stable active components for EGOR are hydroxides. As shown in Fig. 3C, comparing with the Fourier transform infrared (FTIR) spectra at OCP, the absorption band at 1068 cm^−1^ is assigned to the aldehyde stretching of glyoxal (*34*). Representative absorption bands of formate at 1230, 1326, 1412, and 1585 cm^−1^ belong to the C─O stretching, the antisymmetric and symmetric stretching of COO^−^, and the C─O asymmetric stretching (*35*–*38*). Furthermore, no signal of bridge-bonded CO (CO_B_, 1840 to 1900 cm^−1^) or linearly bonded CO (CO_L_, ~2050 cm^−1^) means that EGOR proceeds without CO intermediate (*39*).

### Computations based on density functional theory

Density functional theory calculations were processed over the typical (101) facet of Mo-Ni(OH)_2_ and Ni(OH)_2_ configurations (fig. S55). The density of states analysis suggests that the d-band center of Mo-Ni(OH)_2_ (−5.25 eV) is more closely situated to the Fermi energy level than that of Ni(OH)_2_ (−5.64 eV), indicating that the construction of FLPs promotes intrinsic conductivity (fig. S56). Differential charge density distribution analysis shows the enhanced local electron transfer between adsorbed EG and active sites, which profitably facilitates the activation of adsorbed EG (fig. S57). Because the integral of crystal orbital Hamilton population (ICOHP) reflects the amount of overlapping electron orbitals in chemical bonds (fig. S58), more positive ICOHP indicates weaker strength and thermodynamically favorable cleavage ability of C─C bond in adsorbed EG, which is in accordance with the longer C─C bond length of EG-Mo-Ni(OH)_2_ (1.523 Å) than in EG-Ni(OH)_2_ (1.513 Å) (*40*). Furthermore, Gibbs free energy profiles of EGOR are studied along the stepwise oxidation pathway (* + CH_2_OHCH_2_OH → *CHOHCHOH → *CHO + *CHO → *HCOOH + *HCOOH; Fig. 3D and fig. S59). Notably, the rate-determining step (RDS) with the maximal Gibbs energy barrier of EGOR for Ni(OH)_2_ is the formate desorption (1.09 eV), and RDS for Mo-Ni(OH)_2_ is the oxidation of glyoxal to formate (0.80 eV), indicating that EGOR proceeds with more favorable reaction kinetics on FLPs (Fig. 3E). Moreover, the higher EG adsorption energy and lower formate desorption energy of Mo-Ni(OH)_2_ indicate that FLPs conductively tune the binding ability and barely influence the hydroxyl affinity (figs. S60 and S61). As indicated above, the possible EGOR pathway is illustrated in fig. S62. In detail, EG initially reacts with Mo-NiOOH and dehydrogenates to glyoxal without dehydrogenation of *CO. Subsequently, electrons transfer from the electron-donating Mo sites to the electron-accepting Ni in FLPs, and adsorbed dissociative OH^−^ species activate glyoxal oxidation to formate, as further confirmed by LSV curves under different intermediates (fig. S63).

### Electrocatalysis of hydrogen evolution

We also showed the excellent hydrogen evolution reaction (HER) activity of NiO/NiMoO*_x_* through annealing the NiMoO_4_·*x*H_2_O precursor under H_2_/Ar atmosphere. The XRD pattern and TEM characterization indicate the typical (101) plane of the NiO state in NiO/NiMoO*_x_* (figs. S64 and S65). Impressively, NiO/NiMoO*_x_* only requires overpotentials of 75 and 126 mV to drive 100 and 300 mA cm^−2^ (fig. S66). Tafel slope of NiO/NiMoO*_x_* (40.5 mV dec^−1^) indicates that the RDS for HER is the Heyrovsky step (H* + e^−^ + H_2_O → H_2_ + OH^−^; fig. S67) (*41*). This remarkably enhanced HER activity is attributed to the high conductivity, electrochemical active sites, and *H adsorption ability of the NiO/NiMoO*_x_* catalyst after annealing reduction (figs. S68 to S72). As shown in figs. S73 and S74, hydroxyl species coupled with nickel sites formed flower-like NSs during the Volmer step (H_2_O + e^−^ → OH^−^ + H*). This reconstruction from NiO/NiMoO*_x_* to *OH-NiO/NiMoO*_x_* is confirmed by the nonhydrogen-bonded O─H stretching vibration of hydroxyls at 3638 cm^−1^ in the FTIR spectra and *E_g_*(*T*) phonons of Ni(OH)_2_ at 502 cm^−1^ in the Raman spectra (figs. S75 and S76) (*24*, *42*). Decreasing signals of Ni^0^ at 851.4 eV indicates the conversion from metallic state to hydroxides (fig. S77) (*43*). Impressively, the reconstructed *OH-NiO/NiMoO*_x_* catalyst exhibits excellent HER stability for over 350 hours under a current density of 100 mA cm^−2^ with nearly 100% FE (figs. S78 and S79). Generated hydroxyl species with higher electronegativity contribute to improving the resistance ability of chlorine corrosion and strengthen HER stability (fig. S80).

### Electrocatalysis of PET upcycling in natural seawater

**Fig. 4.**
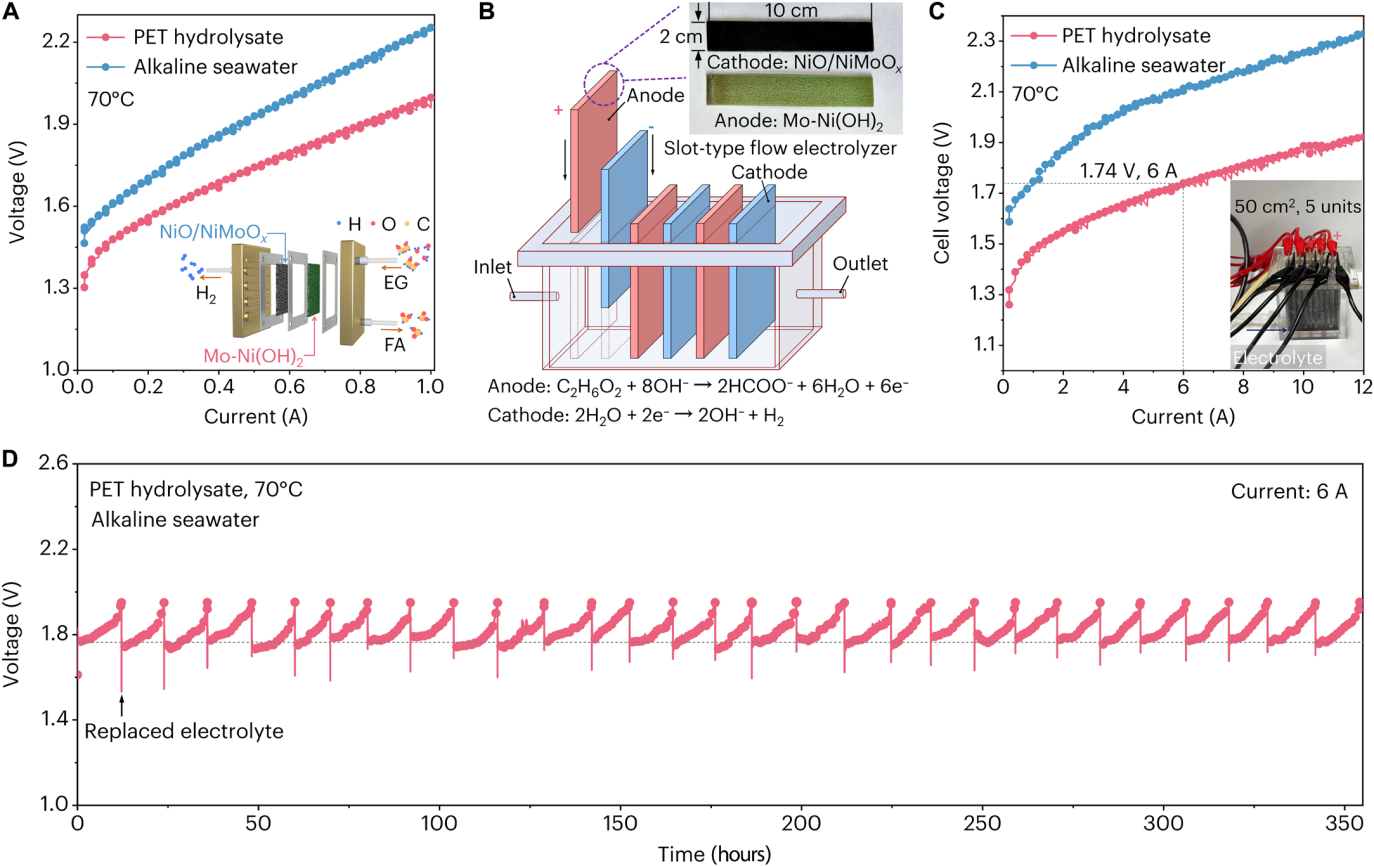
Electrocatalytic activities of electrochemical PET upcycling. (**A**) LSV curves for PET hydrolysate electrolysis at the commercial titanium alloy–made flow electrolyzer. (**B**) Schematic illustration of the slot-type parallel flow electrolyzer. Inset exhibits scale-up anode and cathode. (**C**) LSV curves for PET hydrolysate electrolysis at the five-unit parallel flow electrolyzer. (**D**) CP test for PET hydrolysate electrolysis at 6 A.

Considering that the as-developed catalysts show excellent half-cell activity, we integrated NiO/NiMoO*_x_* as cathode and Mo-Ni(OH)_2_ as anode to extend the promising feasibility of our proposed electrochemical PET upcycling strategy under natural seawater at the device level. Impressively, replacing OER with EGOR combined with HER holds great technical significances on energy-saving consumption, eliminating oxygen/hydrogen explosion risk and avoiding expensive ion exchange membranes (fig. S81). Inspired by the above results, ground PET powders (100 mesh, 1 μm in grain size) from commercial plastic bottles were hydrolyzed into PTA (79%) and EG (21%) at 70°C in 4.0 M KOH, with high conversion efficiency of nearly 100% after 24 hours (fig. S82). Hence, PET hydrolysate under alkaline natural seawater was used as the electrolyte for the electrolysis at the commercial titanium alloy–made flow electrolyzer (fig. S83). As shown in Fig. 4A and fig. S84, the PET hydrolysate electrolysis exhibits excellent stability and saves energy consumption by more than 9% than alkaline seawater electrolysis. However, because scale-up electrolysis requires heating (60° to 80°C) and high operating current to improve efficiency, the expensive device cost of titanium alloy (460 USD kW^−1^) remarkably increases as the catalyst amplification. Furthermore, the compromised resistance of this material to hot alkaline electrolyte generates extra working and maintenance cost (fig. S85).

For the profitable and ampere-level current requirement in industrial applications, we further combined the low-cost polymethyl methacrylate material and sheet structure of metal foam–based catalyst to design the slot-type flow electrolyzer. This practical device is small sized, lightweight, and can easily realize catalyst replacement according to the required working condition (Fig. 4B). In this work, we use five pairs of catalysts to assemble a five-unit parallel flow electrolyzer (working area of 50 cm^2^; fig. S86). LSV curves in Fig. 4C show that this device only requires 1.74 V for PET hydrolysate electrolysis to drive an industrial demand current of 6 A and saves energy consumption by more than 17% than seawater electrolysis. Impressively, the constant PET hydrolysate electrolysis was achieved at a current of 6 A for more than 350 hours under alkaline natural seawater (Fig. 4D and fig. S87). If the EG of PET hydrolysate is mainly converted to formate, then the applied voltage will markedly increase by 0.42 V compared to that of the initial state (fig. S88); hence, the reaction process of PET hydrolysate upcycling can be estimated through the increasing applied voltage.

### Product analysis of electrochemical PET upcycling

**Fig. 5.**
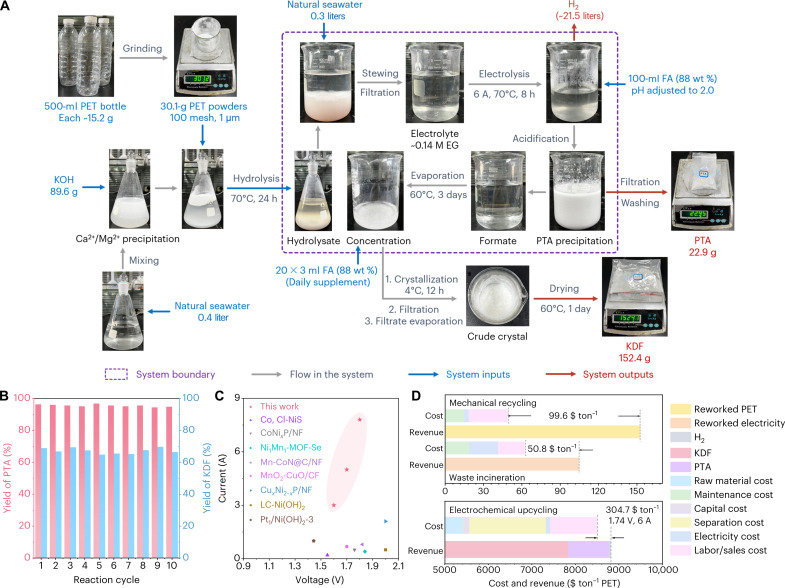
Technical route of electrochemical PET upcycling. (**A**) The schematic illustration for the technical route of electrochemical PET upcycling. (**B**) Yields of PTA and KDF under the same 10 cycles of operations. (**C**) Comparison of an energy-saving system driven by alcohol oxidation with recently reported catalysts. (**D**) Technoeconomic analysis of mechanical recycling, waste incineration, and electrochemical upcycling. h, hours.

Constructing the closed-loop technical route to recycle reactants and products is critical to promote the practical engineering value of electrochemical PET upcycling. However, most of the previous researches failed to further separate liquid products from the electrolyte. Therefore, we proposed the direct downstream conversion process (Fig. 5A), and supplementary formic acid (FA) for acidizing electrolyte can simultaneously recycle unreacted KOH, precipitate PTA, and react with formate to produce KDF, which is an important growth promoter in animal feed approved by the European Union to replace antibiotics (*44*). The notable solubility (room temperature) difference of KDF (337 g/100 ml) allows it to be easily separated from several highly abundant salts in seawater [e.g., NaCl (35.9 g/100 ml), KCl (34.2 g/100 ml), and Na_2_SO_4_ (28.9 g/100 ml)] through evaporation concentration and cooling crystallization. Namely, KDF was obtained through filtering the initially precipitated crystals and then evaporating the remaining filtrate.

Owing to the inevitable volatilization of FA during evaporation concentration, the replenishment of FA is important to increase the purity of KDF. Notably, the most time-consuming crystallization process can make full use of the solar energy in the offshore area to save energy consumption. According to the experiment, we successfully upcycled 301.0 g of PET powders ground from waste plastic bottles to PTA (95.5% average yield; 227.1 g), KDF (67.2% average yield; 1476.2 g), and concomitant H_2_ (~214.9 liters) under repetitive operations (Fig. 5B and figs. S89 and S90). XRD and FTIR results of homemade PTA and KDF further confirm the feasibility of our proposed technical route (fig. S91). Impressively, this excellent performance under industrial demand current takes the leading position among the existing energy-saving systems driven by alcohol oxidation (Fig. 5C and table S4), which also holds the highest PET conversion efficiency than other routes of thermocatalysis, enzyme catalysis, and photocatalysis (table S5).

The subsequent technoeconomic analysis exhibits that electrochemical PET upcycling under the given condition in this work profits $304 USD for upcycling per ton of waste PET and is around three times more profitable than traditional PET recycling routes (Fig. 5D and details in the Supplementary Materials), highlighting the economic superiority of this innovative catalyst/reactor/technology design. Further improvement of revenue still requires optimization of critical technical parameters. Life cycle assessments are conducted to discuss the environmental effect of greenhouse gas emissions, terrestrial ecotoxicity, marine ecotoxicity, and human noncarcinogenic toxicity. The use of chemicals and waste disposal is a main factor that causes CO_2_ emission and environmental pollution. Because of the large amount of CO_2_ emissions generated by the requirement of upstream feedstock KOH, the CO_2_ emission of our technology is only slightly lower than waste incineration. In addition, mechanical recycling produces large waste emissions owing to the low recycle rate; the higher recycle rate of electrochemical PET upcycling effectively reduces the toxicity of chemical pollution to the environment and human health (fig. S92 and table S6) (*45*).

## DISCUSSION

Alkaline depolymerization of PET combined with hydrolysate upcycling holds great engineering value for alleviating the increasingly severe plastic crisis. However, its widespread implementation is limited by the keen demand of water feed, specifically for inland regions where fresh water is scarce. Thus, we believe that deploying this technology in offshore areas is practically meaningful due to virtually limitless access for seawater resources, vast wind, and solar energy for renewable electricity and local chlor-alkali industry for acquiring NaOH/KOH. In this work, we successfully developed hydrolysis and electrolysis in a technically and economically feasible route for upcycling waste PET plastics into value-added PTA, KDF, and concomitant H_2_ using natural seawater. We created a nickel-molybdenum catalyst that effectively facilitated C─C bond cleavage and maintained stability under spontaneous EG reduction, achieving an unprecedented current of 6 A at 1.74 V for more than 350 hours, with a revenue of $304 USD per ton of processed PET. Together, our innovative catalyst/reactor/technology design with PET upcycling efficiency sets up a substantial foundation for the industrialization and commercialization of electrochemical PET upcycling strategy, bridging the critical borders between waste management, seawater electrolysis, and large-scale industrial application.

## MATERIALS AND METHODS

### Chemicals

All chemicals in this work were purchased and used without further purification. Ni(NO_3_)_2_·6H_2_O, Fe(NO_3_)_3_·9H_2_O, (NH_4_)_6_Mo_7_O_24_·2H_2_O, KOH, urea [CO(NH_2_)_2_], and EG (C_2_H_6_O_2_) were purchased from Aladdin Biochemical Technology Co. Ltd. in China. Methanol (CH_4_O), ethanol (C_2_H_5_O), FA (CH_2_O_2_; 88 wt %), glycolic acid (C_2_H_4_O_3_), gloxal (C_2_H_2_O_2_), NH_4_NO_3_, and hydrochloric acid were purchased from Macklin Biochemical Co. Ltd. in China. NFs (1.5 mm in thickness and 350 g cm^−2^ in areal density) were purchased from Kunshan Lvchuang Technology Co. Ltd. in China. Deionized (DI; 18.2 ohm cm^−1^) water used in this work was standard solutions. Commercial water PET bottles were grinded to powders of 100 mesh.

### Synthesis of NiMoO_4_·*x*H_2_O, Mo-NiOOH, and Mo-Ni(OH)_2_

A piece of bare NF (4 cm²) was ultrasonically washed under conditions of 3 M HCl, ethanol, and DI water. Subsequently, the mixture consisted of 30 ml of DI water, 1.2 mmol of Ni(NO_3_)_2_·6H_2_O, and 0.3 mmol of (NH_4_)_6_Mo_7_O_24_·2H_2_O was stirred for 10 min in the Teflon autoclave. Next, the cleaning NF was immersed into the above solution and kept at 150°C for 6 hours. After the temperature was cooled down, the NiMoO_4_·*x*H_2_O precursor was rinsed by DI water and dried in air. The Mo-NiOOH catalyst was synthesized by a chronoamperometry method under a three-electrode system in 1.0 M KOH at a current density of 100 mA cm^−2^ for 20 min, in which the NiMoO_4_·*x*H_2_O precursor (1.0 cm^2^) was used as the working electrode, the graphite rod was used as the counter electrode, and the Hg/HgO electrode served as the reference electrode. The synthesized Mo-NiOOH catalyst was immersed into a 1.0 M KOH electrolyte containing 0.1 M EG under stirring, and black Mo-NiOOH was spontaneously reduced to green Mo-Ni(OH)_2_. The loading mass was measured to be 14.2 mg cm^−2^.

### Synthesis of Ni(OH)_2_ NSs

The mixed solution for the hydrothermal treatment consisted of 30 ml of DI water, 2 mmol of Ni(NO_3_)_2_·6H_2_O, 10 mmol of urea, and 4 mmol of NH_4_F was stirred for 10 min in the Teflon autoclave. Subsequently, a piece of cleaning NF (4 cm²) was immersed into the above solution and kept at 120°C for 6 hours. After the temperature cooled down, the green Ni(OH)_2_ catalyst was rinsed by DI water and dried in air overnight. The loading mass was measured to be 7.8 mg cm^−2^.

### Synthesis of NiO/NiMoO*_x_*

The obtained NiMoO_4_·*x*H_2_O precursor was annealed at 450°C under a ramp rate of 5°C min^−1^ at H_2_/Ar (10:90, 80 standard cubic centimeter per minute) atmosphere for 2 hours.

### Synthesis of RuO_2_ and 20 wt % Pt/C on NF

The mixed solution consisted of 5.0 mg of commercial RuO_2_ or 20 wt % Pt/C powders, 30 μl of Nafion, and 600 μl of isopropanol was ultrasonically treated for 1 hour. Next, the above mixture was slowly dropped on the cleaning NF under red light.

### Characterizations

The morphology was analyzed by SEM (ZEISS, Sigma 300) and TEM (Thermo Fisher Scientific, Talos F200i). The crystal structure was analyzed by XRD (Bruker D8 Advance, Cu Kα x-ray source; 2° min^−1^). Chemical states were detected by XPS (Thermo Fisher Scientific, K-Alpha, Al-Kα x-ray source). In situ FTIR (Nicolet iS 10) and Raman spectra (LabRAM HR Evolution; λ = 532 nm) were controlled on the electrochemical workstation (CHI 760E). Elemental contents were analyzed by ICP-OES (Agilent, 7700s). Products of EGOR were qualitatively determined by NMR spectroscopy (500 MHz; Bruker Avance III HD), in which 500 μl of electrolyte was mixed with 500 μl of D_2_O and 30 μl of dimethyl sulfoxide. Products of EGOR were quantitatively analyzed by HPLC (Agilent 1260) equipped with an organic acid column (Coregel 87H3), a wavelength detector (210 nm), and a mobile phase of 5 mM H_2_SO_4_ aqueous solution (60°C, 0.6 ml min^−1^).

### EXAFS measurement and analysis

EXAFS spectroscopy was conducted at the BL11B beamline of the Shanghai Synchrotron Radiation Facility (SSRF). All EXAFS data were processed in Athena software (version 0.9.26) for background, preedge line, and postedge line calibrations. Coordination number, bond length, Debye-Waller factor, and *E*_0_ shift (CN, R, and Δ*E*_0_) were set, and Fourier transform *k*^3^-weighted (*k*) quantitative curves in the *R* space were fitted in the Artemis software (version 0.9.26). The *K*-range of 3 to 11 Å^−1^ and *R* range of 1 to 3 Å were used for fitting the data.

### Electrochemical measurement

All electrochemical measurements under the standard three-electrode system were acquired on the electrochemical workstation (CHI 760E), in which graphite rod was used as the counter electrode, Hg/HgO (1.0 M KOH) electrode served as the reference electrode, and the geometric area of the working electrode is 1.0 cm^2^. Natural seawater was obtained from Bohai Sea (5 to 10 m in depth) in the Huludao city of Liaoning province in China. The pH values of 1.0 M KOH and 1.0 M KOH + seawater were measured to be 13.8 and 13.7. All measured potentials with 85% *iR* correction were transferred to versus RHE according to the following equation: *E*_RHE_ = *E*_Hg/HgO_ + 0.098 + 0.059 × pH. All LSV curves were recorded at a scan rate of 2 mV s^−1^. EIS were tested from 0.1 to 10,000 Hz with an amplitude of 5 mV. Ampere-level current test was conducted at the Neware BTS-5V12A power system.

### Flow electrolyzer fabrication

All chronopotentiometry (CP) tests in the homemade flow electrolyzer (fig. S34) were conducted without *iR* correction, and 0.05 liter of electrolyte was daily supplied to the 1 liter of beaker according to the regular consumption during long-term CP test for EGOR and HER. The slot-type five-unit flow electrolyzer was made of acrylic glass and connected to the power station by a pair of one-in and five-out copper clips. The electrolyzer was 6 cm high, 3 cm wide, and 5 cm long. The water outlet was 6 mm in diameter and located at a height of 4.5 cm. Each slot was 3 mm thick and 2 cm apart, and prepared anode and cathode were interleaved into a slot for use. The PET hydrolysate electrolyte with a volume of 0.7 liter at a flow rate of 10 ml min^−1^ was controlled by the peristaltic pump, and it was kept at 70°C under stirring. As for the long-term stability test under ampere-level current, 50 g of PET powders were added to the alkaline seawater solution and heated at 70°C under stirring for 24 hours. After the sediment was filtered, this solution was used as the electrolyte for the stability test at 70°C under stirring. Because of water evaporation and continuous consumption of KOH, the cutoff voltage was set to be 1.95 V, and then operation was repeated after replacing the fresh electrolyte. The catalysts were stored in the flowing 2 wt % EG solution during the non-testing period.

### Product separation

After PET hydrolysate electrolysis, 100 ml of FA (88 wt %) was slowly added into the electrolyte to adjust the pH to 3.0 under stirring. After the temperature was cooled down, PTA precipitations were filtrated and washed for several times. Afterward, the filtrate was concentrated at 60°C under stirring for 3 days, with a daily supplement of 20 ml of FA (88 wt %) until the crystals precipitated in the solution. Afterward, the hot solution was cooled at 4°C for 12 hours. Last, the KDF product was obtained through filtering the initially precipitated crystals and then evaporating the remaining filtrate.

## Supplementary Materials

### This PDF file includes:

Supplementary Text Figs. S1 to S92 Tables S1 to S6 References

Correction (13 August 2025):
In the originally published version of the Supplementary Materials, fig. S29 was inadvertently a duplicate of fig. S30, and eq. S2 mistakenly included “✕ F” at the end of the equation. The Supplementary Materials have been updated to correct these errors. The original version is available here:
