# Supplementary material for: Geography-guided industrial-level upcycling of polyethylene terephthalate plastics through alkaline seawater-based processes
Source: Sci Adv. 2025 May 28;11(22):eadu8381. doi: 10.1126/sciadv.adu8381 (PMC12118594; doi:10.1126/sciadv.adu8381)
Supplement: Supplementary file 2 — Correction (13 August 2025): In the originally published version of the Supplementary Materials, fig. S29 was inadvertently a duplicate of fig. S30, and eq. S2 mistakenly included “✕ F” at the end of the equation. The Supplementary Materials have been updated to correct these errors. The original version is available here: [file sciadv.adu8381_sm.v1.pdf]

Science Advances
AAAS

Supplementary Materials for

Geography-guided industrial-level upcycling of polyethylene terephthalate plastics through alkaline seawater-based processes

Zehao Xiao *et al.*

Corresponding author: Mingchuan Luo, m.luo@pku.edu.cn; Shaojun Guo, guosj@pku.edu.cn

Sci. Adv. **11**, eadu8381 (2025)
DOI: 10.1126/sciadv.adu8381

This PDF file includes:

Supplementary Text
Figs. S1 to S92
Tables S1 to S6
References

Supplementary Text

Methods

Product analysis. The Faradaic efficiency (FE) of target product is calculated through corresponding electron transfer at per reaction from the equation S1:

$$FE = 100\% \times \frac{n_{product} \times n_e}{Q} \times F \quad \text{(S1)}$$

Where n_e is the number of transferred electron, $n_{product}$ is the amount of target product, F is the Faradaic constant (96,485 C·mol^{-1}) and Q is the amount of electric charge. The selectivity of target product is calculated through the amount of total product at per reaction from the equation S2:

$$Selectivity = 100\% \times \frac{n_{product}}{n_{total}} \times F \quad \text{(S2)}$$

Turnover frequency (TOF) calculation. TOF indicates the reactants of per reaction at per active site per time. The premise is that all active sites adequately participate in the whole reaction. The amount of active sites is calculated from the equation S3:

$$n = Q \,/\, (2 \times F) \quad \text{(S3)}$$

Where Q is the amount of voltammetry charge based on cyclic voltammetry (CV) curves under different scan rates in the non-Faradaic region. The TOF is calculated from the equation S4:

$$\mathrm{TOF} = I \,/\, (2 \times n \times F) \quad \text{(S4)}$$

Where I is the current density measured from linear sweep voltammetry (LSV) polarization curves and n is the amount of active sites.

Electrochemical active surface area (ECSA) calculation. ECSA relates to layer capacitance (C_{dl}), which is calculated from plotting the discrepant current densities $\Delta j = (j_a - j_c)$ where the slope of the linearly fits twice time of C_{dl} under different scan rates in the non-Faradic region. C_{dl} is calculated from the equation S5:

$$\Delta j = 2 \times C_{dl} \times v \quad \text{(S5)}$$

Where j is the half of double layered capacitive current density (mA·cm^{-2}), geometric area of the electrode is 1.0 cm^2, capacitance value at per area is 40 μF·cm^{-2} and v is the scan rate (mV·s^{-1}).

Computational details. All spin-polarized density functional theory (DFT) methods were performed using the Vienna ab-initio simulation package (VASP) code through the projector augmented wave (PAW) method.(*46, 47*) The generalized gradient approximation (GGA) combined with Perdew-Burke-Ernzerhof (PBE) functional formulations were employed to describe the exchange-correlation term.(*48*) The projector augmented wave (PAW) pseudo-potentials were employed to describe ionic cores.(*47*) The Brillouin zone was sampled with 1 × 5 × 1 Monkhorst-Pack k-point for the geometry optimization and the entire model contained 5 layers. A vacuum layer of 15.83 Å along the b-direction was applied to separate surface and sufficiently avoid periodic molecular interaction. The cut-off energy for the plane-wave basis was set to be

500 eV. The Van der Waals (VDW) interactions were described through the empirical correction in Grimme's scheme (DFT-D3) in all calculations.(*49*) Partial occupancies of the Kohn-Sham orbitals were allowed using the Gaussian smearing method with the width of 0.05 eV. The electronic energy was considered self-consistent when the energy change was smaller than 10^{-5} eV. The geometry optimization was considered convergent when the energy change was smaller than 0.05 eV·Å^{-1}. The DFT + U methodology was used to solve the on-site coulomb and exchange interactions of the strongly localized electrons of Ni 3d and Mo 3d. The U values for Ni and Mo in Mo-Ni(OH)$_2$ were respectively set to be 6.0 and 3.5 eV.(*50*) The U values of Ni and Mo in $NiMoO_4$ are set to 6.2 and 4.4 eV, while the U value of Ni in NiO is set to 8.0 eV. The adsorption energy (E_{ads}) is calculated from the equation S6:

$$E_{ads} = E_{ad/sub} - E_{ad} - E_{sub} \tag{S6}$$

Where $E_{ad/sub}$ is the total energy of the optimized adsorbate/substrate system, E_{ad} is the total energy of the adsorbate in the structure and E_{sub} is the total energy of the substrate.

The Gibbs free energy (ΔG) for each elemental step is calculated from the equation S7:

$$\Delta G = \Delta E_{DFT} + \Delta E_{ZPE} - T \times \Delta S \tag{S7}$$

Where ΔE_{DFT} is the electronic energy change obtained from DFT calculations, ΔE_{ZPE} is the zero-point energy and ΔS is the entropy change obtained from frequency calculations at 298.15 K.

The transition state for each elemental step was calculated by the Nudged Elastic Band (NEB) method. In the NEB method, the elemental step between reactant and product was discretized into a series of free structural images. Images of intermediates were relaxed until the corresponding perpendicular force was lower than 0.05 eV·Å^{-1}. The reaction energy (G) of different intermediates is calculated from the equation S8:

$$G = G_i - G_{reactant} \tag{S8}$$

Where G_i is the energy of intermediates and $G_{reactant}$ is the total energy of reactants.

Economic model of techno-economic analysis for electrochemical PET upcycling. The assumptive operating condition is 80,000 m^3 alkaline seawater for disposing 3000 tons PET (350 working days·year^{-1}). The current on each reactor is I A. According to the estimated PET hydrolysate oxidation capacity and Faradaic efficiency (95%) of EGOR, total required charge and reaction area are calculated as following:

$$Total\ charge = \frac{3{,}000{,}000\ kg \times 21\%}{62.068 \times 10^{-3}\ kg \cdot mol^{-1}} \times 6\ e^{-} \times \frac{96{,}485\ C}{mol} \times \frac{1}{0.95} = 6.19 \times 10^{12}\ C$$

$$Total\ current = \frac{6.19 \times 10^{12}\ C}{350 \times 24 \times 3600\ s} = 2.05 \times 10^{5}\ A$$

Reaction area is calculated as following:

$$Required\ reactor\ aomount = \frac{2.05 \times 10^{5}}{I}$$

$$Reaction\ area = \frac{2.05 \times 10^5}{I} \times 50 \times 10^{-4}\ m^2 = \frac{1023.5}{I}\ m^2$$

Raw material cost. Assuming that anodic/cathodic catalysts are replaced every 15 days. Sale prices of $Ni(NO_3)_2$, $(NH_4)_6Mo_7O_{24}$, H_2/Ar (10 : 90), KOH and FA (88 wt%) are 4.15 *USD·kg*$^{-1}$, 2.08 *USD·kg*$^{-1}$, 1.05 *USD·kg*$^{-1}$, 0.85 *USD·kg*$^{-1}$ and 0.40 *USD·kg*$^{-1}$, respectively. Assuming that the quality of recycled waste PET is reduced, the sale price of recycled waste PET is set to be 80% of the market price (0.31 *USD·kg*$^{-1}$).

Raw material cost is calculated as following:

$$Ni(NO_3)_2\ cost = 2 \times \frac{1023.5}{I}\ m^2 \times \frac{4.15\ USD}{1000\ g} \times \frac{0.349\ g}{0.001\ m^2} \times 24 = \frac{71154.54}{I}\ USD$$

$$(NH_4)_6Mo_7O_{24}\ cost = 2 \times \frac{1023.5}{I}\ m^2 \times \frac{2.08\ USD}{1000\ g} \times \frac{0.371\ g}{0.001\ m^2} \times 24 = \frac{37911.10}{I}\ USD$$

$$H_2/Ar\ cost = \frac{1023.5}{I} m^2 \times 24 \times \frac{800\ L}{24.5\ L \cdot mol^{-1}} \times \frac{33.4\ g}{1\ mol} \times \frac{1.05\ USD}{1000\ g} = \frac{27789.02}{I}\ USD$$

$$KOH\ cost = 80{,}000\ m^3 \times \frac{89.6\ g}{800\ mL} \times \frac{0.85\ USD}{1000\ g} = 7.62 \times 10^6\ USD$$

$$Formic\ acid\ cost = 80{,}000\ m^3 \times \frac{160\ mL}{800\ mL} \times \frac{1.22\ g}{1\ mL} \times \frac{0.40\ USD}{1000\ g} = 7.81 \times 10^6\ USD$$

$$Waste\ PET\ cost = 3{,}000{,}000\ kg \times 80\% \times \frac{0.31\ USD}{1000\ g} = 7.44 \times 10^5\ USD$$

$$Raw\ material\ cost = (1.62 \times 10^7 + \frac{136854.66}{I})\ USD$$

Total revenue. Assuming that the Faradaic efficiency of HER is 98%, yield rate of PTA is 95% and yield rate of KDF is 67%. Based on current market prices and references,(*13, 15*) sale prices of KDF, PTA and H_2 are 1.59 *USD·kg*$^{-1}$, 1.26 *USD·kg*$^{-1}$ and 1.90 *USD·kg*$^{-1}$. Product revenue is calculated as following:

$$H_2\ revenue = \frac{6.19 \times 10^{12}\ C}{2 \times 96{,}485\ C \cdot mol^{-1}} \times 2\ g \cdot mol^{-1} \times \frac{1.9\ USD}{1000\ g} \times 0.98 = 1.19 \times 10^5\ USD$$

$$KDF\ revenue = 80{,}000\ m^3 \times \frac{1.7\ mol}{800\ mL} \times 67\% \times 130.14\ g \cdot mol^{-1} \times \frac{1.59\ USD}{1000\ g}$$
$$= 2.35 \times 10^7\ USD$$

$$PTA\ revenue = 3{,}000{,}000\ kg \times 79\% \times 95\% \times \frac{1.26\ USD}{1000\ g} = 2.83 \times 10^6\ USD$$

$$Total\ revenue = 2.64 \times 10^7\ USD$$

Capital cost. In this work, we use acrylic glass as the origin material for constructing the electrolyzer and its stack cost is set to be 20% of titanium alloy (460 USD/kW). The balance of plant (BoP) cost is assumed to be 35% of the total cost of the electrolyzer, and these values are calculated from the H2A model.(*51*) The working voltage is *U* V.

$$Electrolyzer\ cost = \frac{92\ USD}{1000\ W} \times 2.05 \times 10^5\ A \times U\ V = (18860 \times U)\ USD$$

$$BoP\ cost = 18860 \times U\ USD \times \frac{0.35}{0.65} = (10155.38 \times U)\ USD$$

PSA cost relates to the outlet gas flow rate, which is calculated according to the reference.(*51*)

$$Volume\ of\ H_2 = \frac{6.19 \times 10^{12}\ C}{2 \times 96{,}485\ C \cdot mol^{-1}} \times 24.5\ L \cdot mol^{-1} \times 0.98 = 7.70 \times 10^5\ m^3$$

$$PSA\ cost = 1{,}989{,}043\ USD \times (\frac{7.70 \times 10^5\ m^3}{350 \times 24\ h \times 10^3\ m^3 \cdot h^{-1}})^{0.7} = 3.74 \times 10^5\ USD$$

$$Captial\ cost = (3.74 \times 10^5 + 29015.38 \times U)\ USD$$

Operating cost. Assuming that the cost of industrial electricity (Beijing, China) is 0.05 $USD\cdot(kW\cdot h)^{-1}$ and the total electricity consumption of the whole technology is twice times than that of the electrolyzer consumption. Electricity cost is calculated as following:

$$Electricity\ cost = 2 \times 2.05 \times 10^5 \times U\ V \times 350 \times 24\ h \times \frac{0.05\ USD}{1000\ W \cdot h}$$
$$= (1.73 \times 10^5 \times U)\ USD$$

Assuming that the working and maintenance cost is 10% of the capital cost.

$$Working\ and\ maintenance\ cost = (3.74 \times 10^5 + 29015.38 \times U) \times 10\%$$
$$= (3.74 \times 10^4 + 2901.54 \times U)\ USD$$

Assuming that hydrogen as the only product of the cathode without separation operation, PET powders need to be hydrolyzed at 70 °C and liquid products need to be separated with 20% cost of revenues for liquid products.

$$Separation\ cost = (2.35 \times 10^7 + 2.83 \times 10^6) \times 20\% = 5.27 \times 10^6\ USD$$

$$Operating\ cost = (5.28 \times 10^6 + 1.76 \times 10^5 \times U)\ USD$$

Other cost. Other costs include pretreatment costs, water costs, labor costs, transportation costs, and sales costs and so on. The value of other cost is set to be 20% of the raw material cost.

$$Other\ cost = (1.62 \times 10^7 + \frac{136854.66}{I})\ USD \times 20\% = (3.24 \times 10^6 + \frac{27370.93}{I})\ USD$$

Retained profit. Retained profit for electrochemical upcycling is calculated as following:

$$\begin{aligned} Total\ cost = {} & 3.24 \times 10^6 + \frac{27370.93}{I} + 5.28 \times 10^6 + 1.76 \times 10^5 \times U + 3.74 \times 10^5 \\ & + 29015.38 \times U + 1.62 \times 10^7 + \frac{136854.66}{I} \\ & = \left(2.51 \times 10^7 + 2.06 \times 10^5 \times U + \frac{164225.59}{I}\right) USD \end{aligned}$$

$$\begin{aligned} Retained\ profit & = \frac{Total\ revenue - Total\ cost}{3000\ tons} \\ & = \left(433.33 - 68.67 \times U - \frac{54.74}{I}\right) USD \cdot ton^{-1} \end{aligned}$$

Techno-economic analysis for mechanical recycling

Total revenue. Assuming that the quality of recycled PET is reduced, the sale price of recycled PET is set to be 80% of the market price (0.31 $USD{\cdot}kg^{-1}$). The recycling efficiency is set to be 60%.

$$PET\ revenue = 3{,}000{,}000\ kg \times 80\% \times \frac{0.31\ USD}{1000\ g} \times 60\% = 4.57 \times 10^5\ USD$$

Capital cost. Mechanical recycling does not require the acquisition of new equipment, its capital cost hence is negligible.

Operating cost. Current commercially available devices for mechanical recycling consume ~200 $kW{\cdot}h{\cdot}(day{\cdot}ton)^{-1}$. Electricity cost is calculated as following:

$$Electricity\ cost = 3000\ tons \times \frac{200 \times 350 \times 0.05\ USD}{1000\ W \cdot h \cdot ton} = 1.05 \times 10^4\ USD$$

The working and maintenance cost is set to be 10% of the total revenue.

$$Working\ and\ maintenance\ cost = 4.57 \times 10^5\ USD \times 10\% = 4.57 \times 10^4\ USD$$

$$Operating\ cost = 5.62 \times 10^4\ USD$$

Other cost. Other costs include pretreatment costs, water costs, labor costs, transportation costs, and sales costs and so on. The value of other cost is set to be 20% of the total revenue.

$$Other\ cost = 4.57 \times 10^5\ USD \times 20\% = 9.14 \times 10^4\ USD$$

Retained profit. Retained profit for mechanical recycling is calculated as following:

$$Retained\ profit = \frac{Total\ revenue - Total\ cost}{3000\ tons} = 99.6\ USD \cdot ton^{-1}$$

Techno-economic analysis for waste incineration

Total revenue. Assuming that the heat of combustion for PET is 25 $MJ{\cdot}kg^{-1}$, the energy efficiency is set to be 30% and the reworked electricity (Beijing, China) is 0.05 $USD{\cdot}(kW{\cdot}h)^{-1}$.

$$Electricity\ revenue = 3{,}000{,}000\ kg \times 30\% \times 25\ MJ \cdot kg^{-1} \times 2.78 \times 10^{-7} \times \frac{0.05\ USD}{1000\ W \cdot h}$$
$$= 3.14 \times 10^{5}\ USD$$

Capital cost. Waste incineration does not require the acquisition of new equipment, its capital cost hence is negligible.

Operating cost. Assuming that the cost of electricity is 450 $USD{\cdot}(kW{\cdot}h{\cdot}ton)^{-1}$. Electricity cost is calculated as following:

$$Electricity\ cost = 3{,}000\ tons \times 450 \times 0.05\ USD = 6.75 \times 10^{4}\ USD$$

The working and maintenance cost is set to be 10% of the total revenue.

$$Working\ and\ maintenance\ cost = 3.14 \times 10^{5}\ USD \times 10\% = 3.14 \times 10^{4}\ USD$$

$$Operating\ cost = 9.89 \times 10^{4}\ USD$$

Other cost. Other costs include pretreatment costs, water costs, labor costs, transportation costs, and sales costs and so on. The value of other cost is set to be 20% of the total revenue.

$$Other\ cost = 3.14 \times 10^{5}\ USD \times 20\% = 6.28 \times 10^{4}\ USD$$

Retained profit. Retained profit for waste incineration is calculated as following:

$$Retained\ profit = \frac{Total\ revenue - Total\ cost}{3000\ tons} = 50.8\ USD \cdot ton^{-1}$$

Life cycle assessment details. Life cycle assessments (LCA) analysis is processed based on the ReCiPe method.(*52*) Terrestrial ecotoxicity, marine ecotoxicity and human non-carcinogenic toxicity are expressed in equivalent of 1,4-dichlorobenzene (1,4-DCB). Terrestrial ecotoxicity refers to the harmful effects of toxic chemicals on terrestrial organisms, mainly including pesticides, heavy metals and organic pollutants. Marine ecotoxicity refers to the impact of toxic chemicals on marine life. Human non-carcinogenic toxicity refers to the non-carcinogenic hazards to human health from exposure to toxic chemicals. The simulated technical conditions in LCA analysis are consistent with the above economic analysis. Products with unsuccessful recycling are treated as solid waste pollutions.

Supplementary Figures

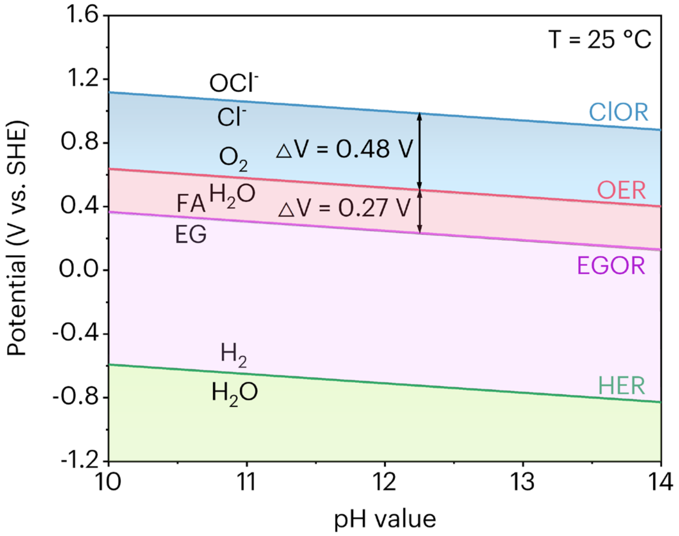

Fig. S1. Thermodynamic equilibrium potential analysis. Pourbaix diagram of HER, EGOR, OER and ClOR under different pH values.

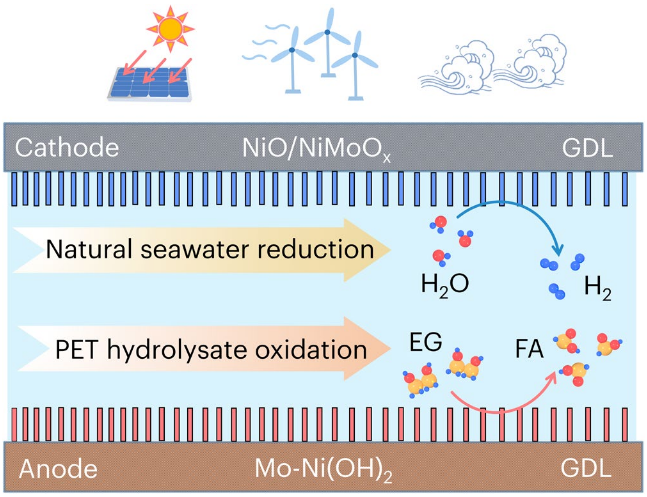

Fig. S2. Technical diagram. Proposed schematic diagram of the hybrid electrolysis system coupling natural seawater reduction and PET hydrolysate oxidation.

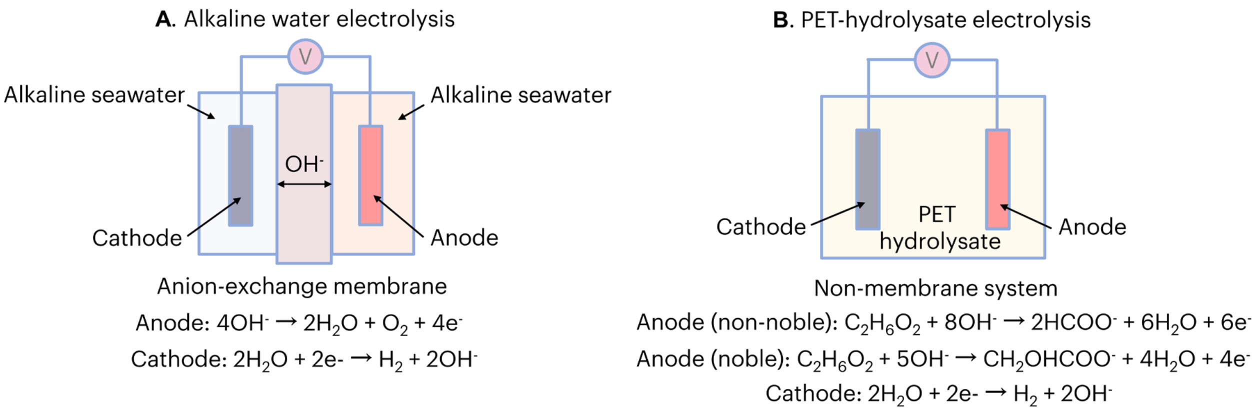

Fig. S3. Technical diagram. Device diagrams of the (**A**) conventional alkaline seawater electrolysis system and (**B**) hybrid PET-hydrolysate electrolysis system.

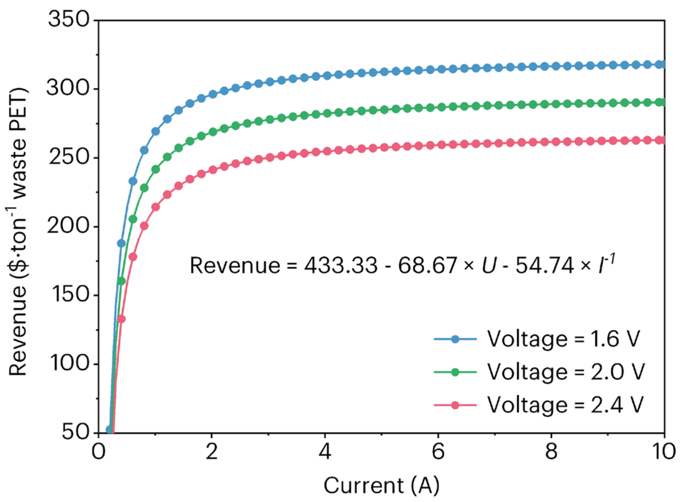

Fig. S4. Techno-economic analysis. Techno-economic analysis of revenue for electrochemical PET upcycling.

.

.

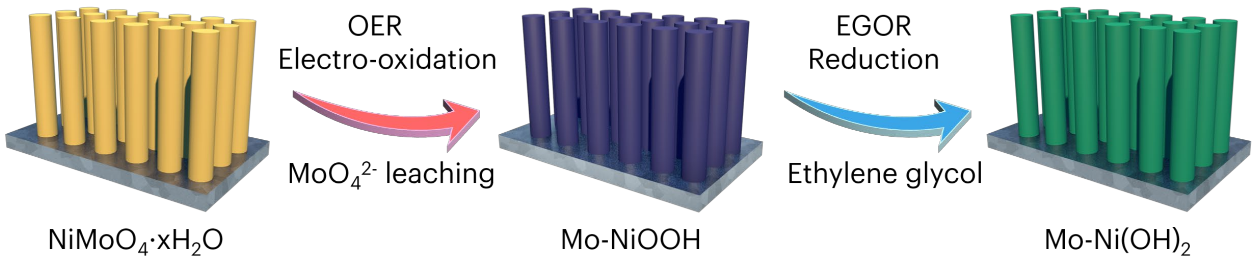

Fig. S5. Synthesis diagram. Schematic illustration of the synthesis process for Mo-$Ni(OH)_2$.

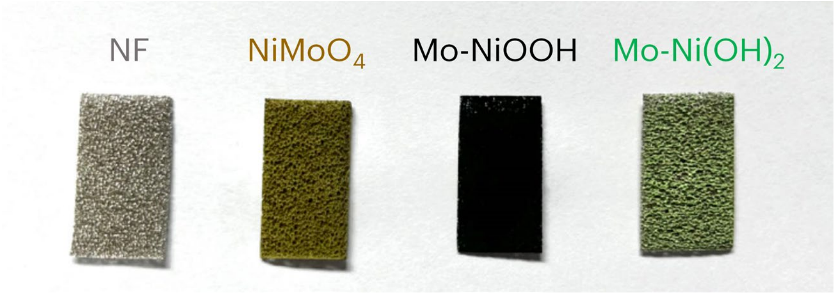

Fig. S6. Real catalyst image. Digital image of bare NF, $NiMoO_4 \cdot xH_2O$ precursor, Mo-NiOOH NRs and Mo-$Ni(OH)_2$ NRs.

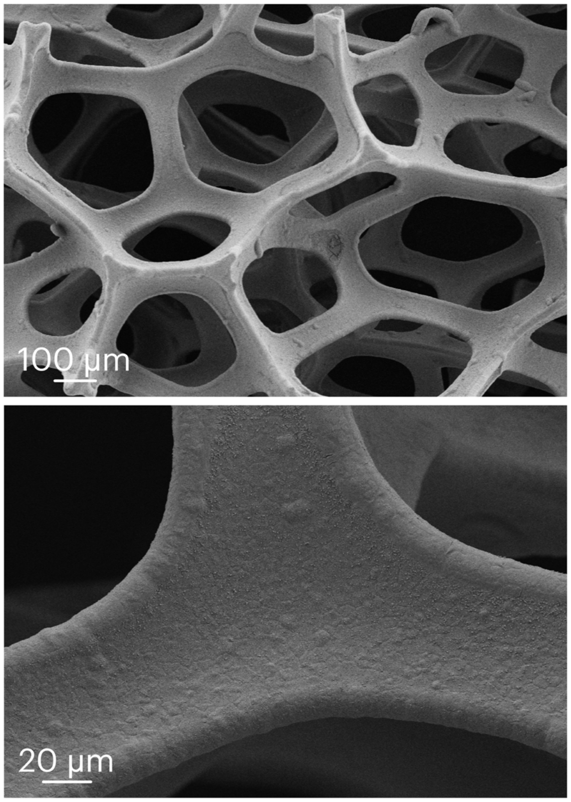

Fig. S7. Structural analysis. Scanning electron microscope (SEM) images of the bare NF.

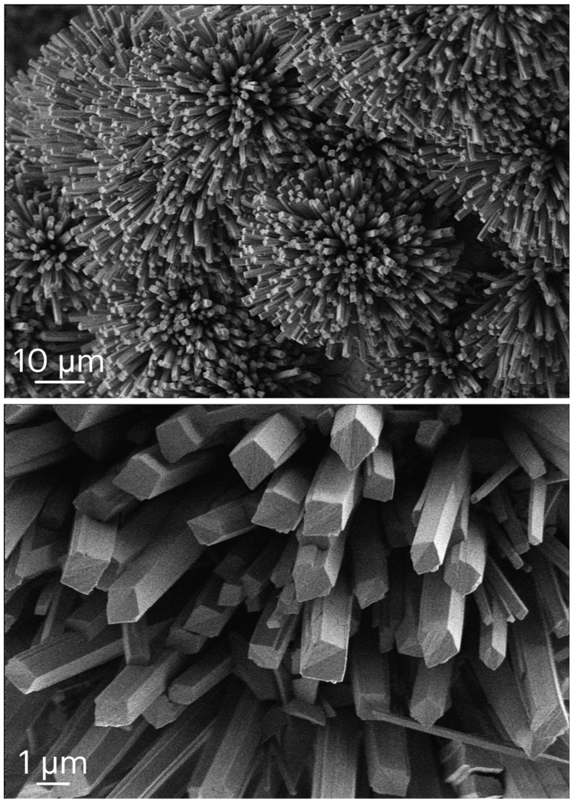

Fig. S8. Structural analysis. SEM images of the $NiMoO_4 \cdot xH_2O$ precursor.

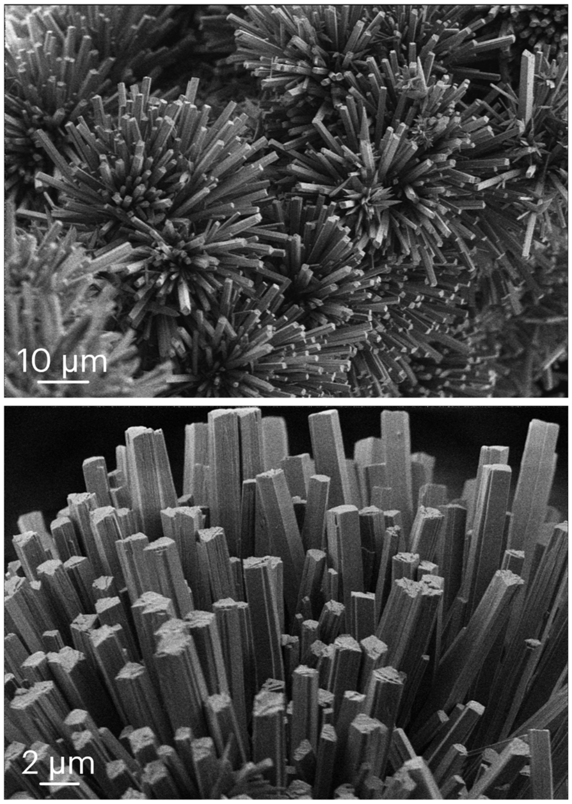

Fig. S9. Structural analysis. SEM images of Mo-NiOOH NRs.

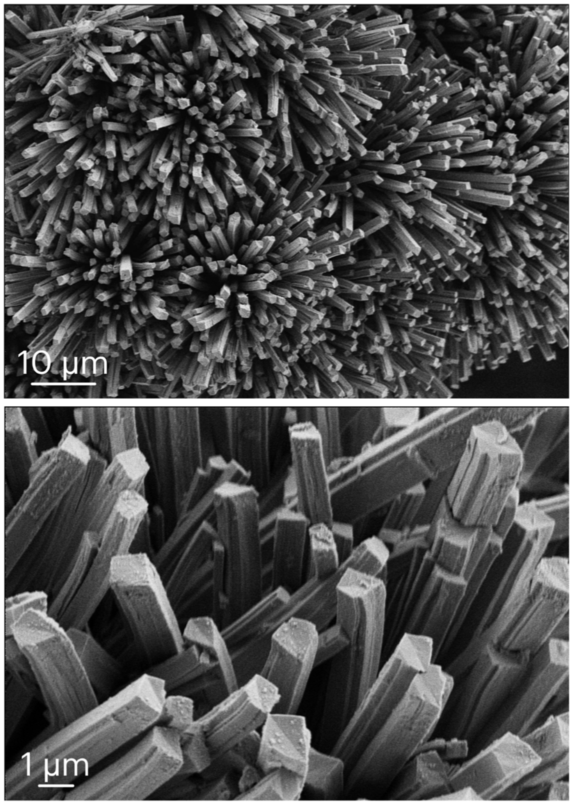

Fig. S10. Structural analysis. SEM images of Mo-Ni$(OH)_2$ NRs.

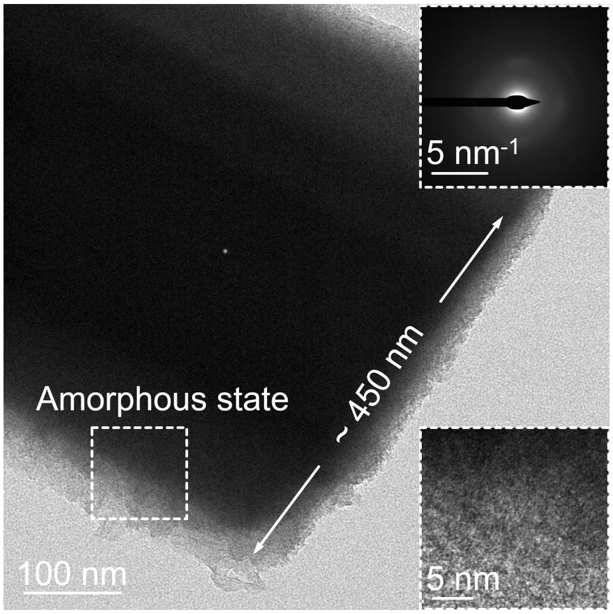

Fig. S11. Structural analysis. Transmission electron microscope (TEM) image with high resolution (HR)-TEM image and corresponding selected area electron diffraction (SAED) pattern of Mo-Ni(OH)$_2$.

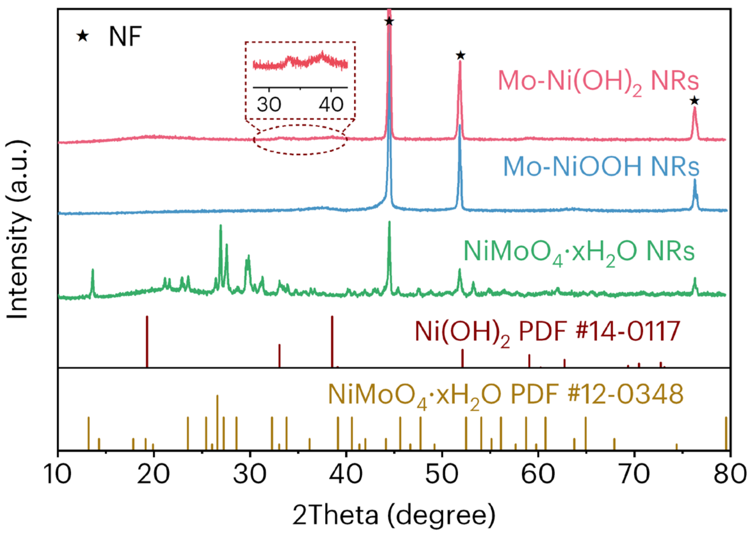

Fig. S12. Structural analysis. X-ray diffraction (XRD) patterns of $NiMoO_4 \cdot xH_2O$, Mo-NiOOH and Mo-$Ni(OH)_2$.

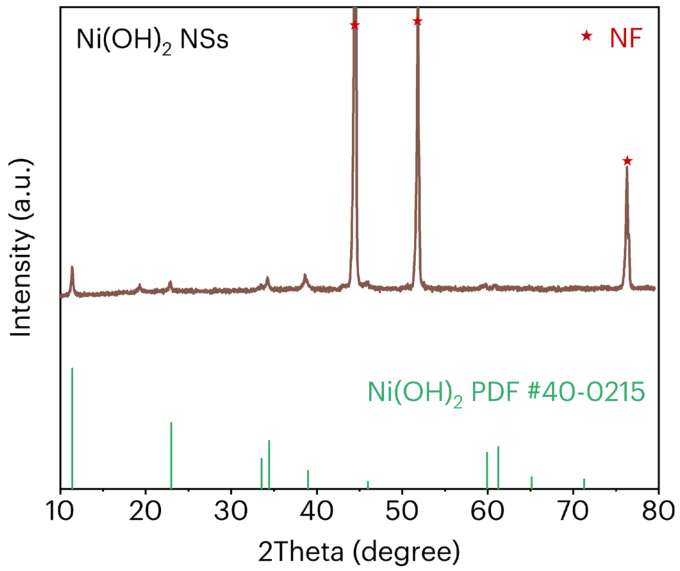

Fig. S13. Structural analysis. XRD pattern of $Ni(OH)_2$ NSs.

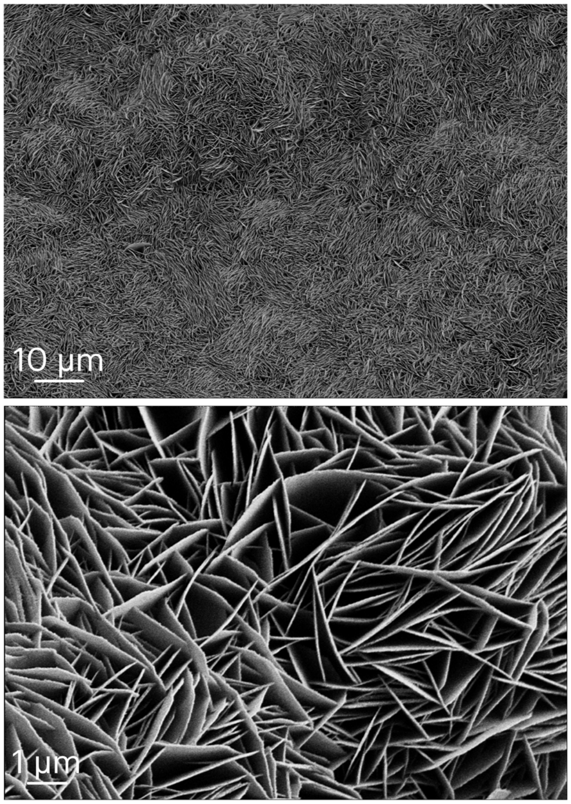

Fig. S14. Structural analysis. SEM images of $Ni(OH)_2$ NSs.

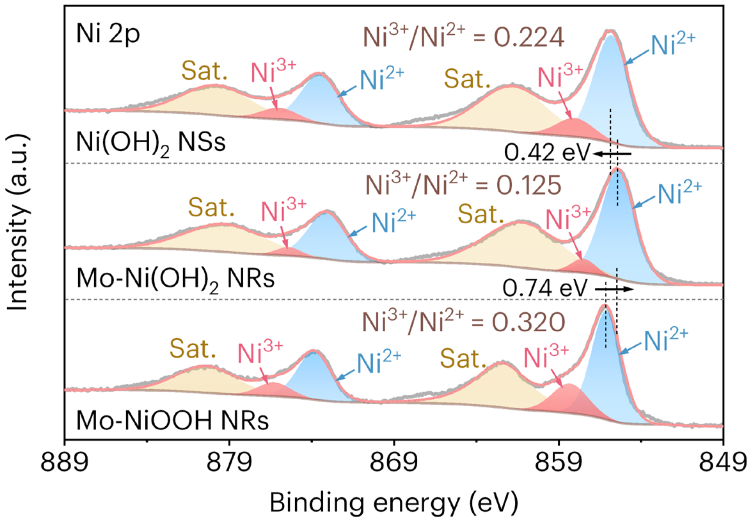

Fig. S15. XPS characterization. High-resolution X-ray photoelectron spectroscopy (XPS) spectra of Ni 2p for Mo-NiOOH, Mo-$Ni(OH)_2$ and $Ni(OH)_2$.

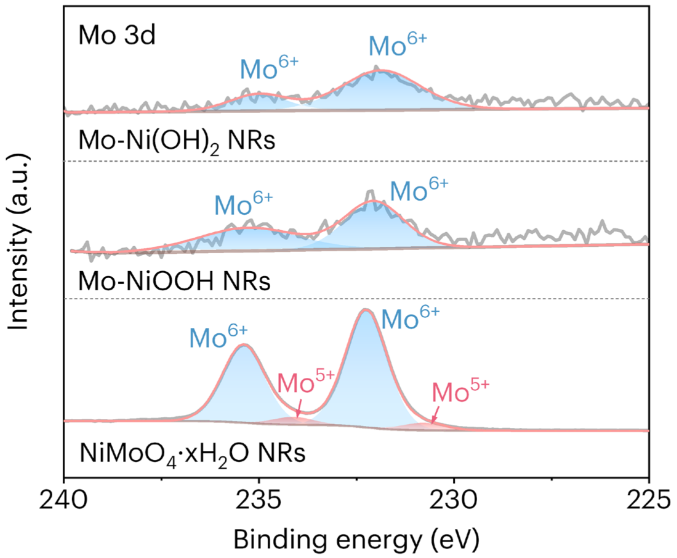

Fig. S16. XPS characterization. High-resolution XPS spectra of Mo 3d for $NiMoO_4 \cdot xH_2O$, Mo-NiOOH and Mo-$Ni(OH)_2$.

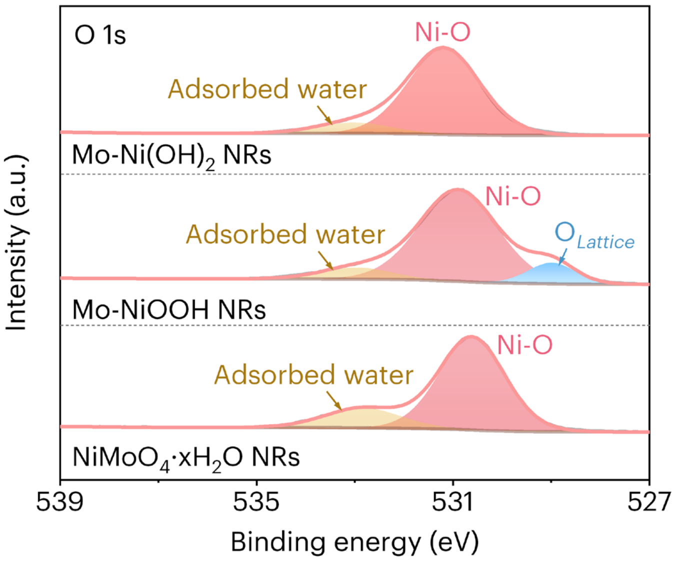

Fig. S17. XPS characterization. High-resolution XPS spectra of O 1s for $NiMoO_4 \cdot xH_2O$, Mo-NiOOH and Mo-$Ni(OH)_2$.

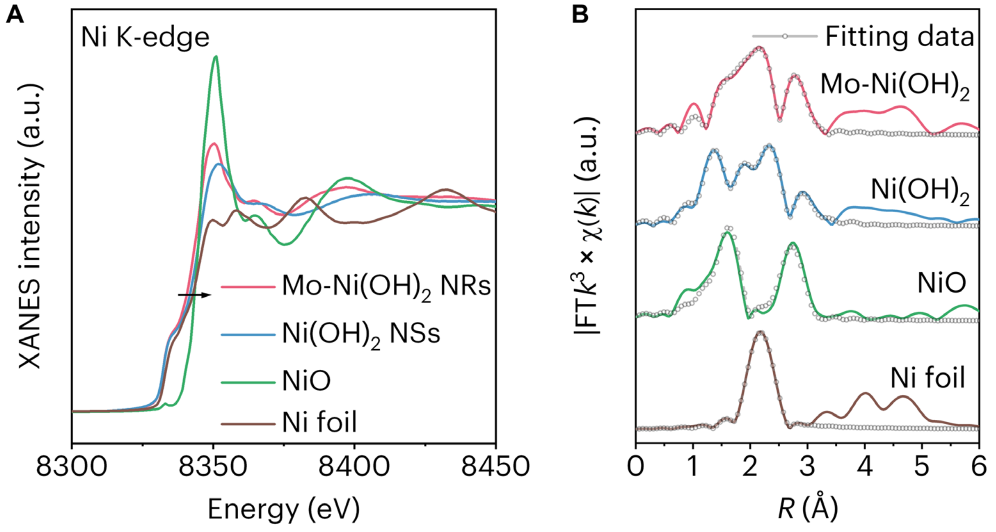

Fig. S18. XANES characterization. (**A**) Normalized X-ray absorption near-edge structure (XANES) spectra and (**B**) Fourier-transform extended X-ray absorption fine structure (EXAFS) spectra at the Ni K-edge for Mo-$Ni(OH)_2$, $Ni(OH)_2$, NiO and Ni foil.

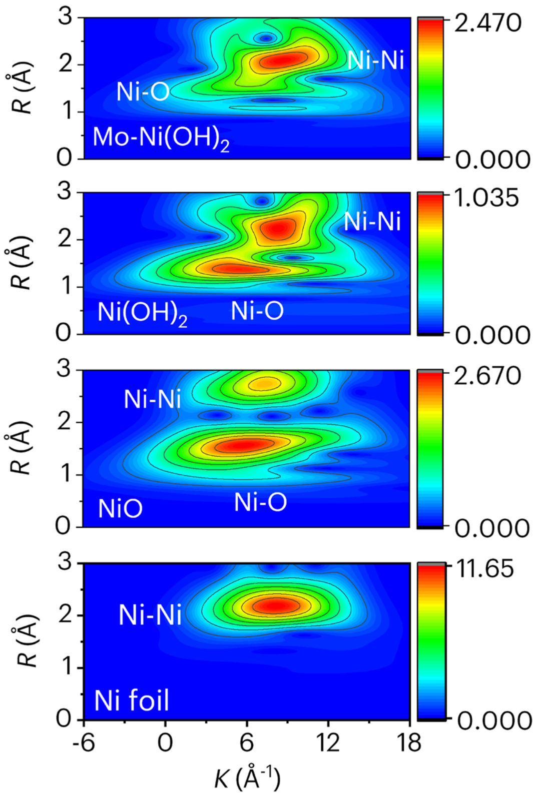

Fig. S19. WT-EXAFS analysis. Wavelet transformed (WT) k^3-weighted $\chi(k)$-function for Mo-$Ni(OH)_2$, $Ni(OH)_2$, NiO and Ni foil at the Ni K-edge.

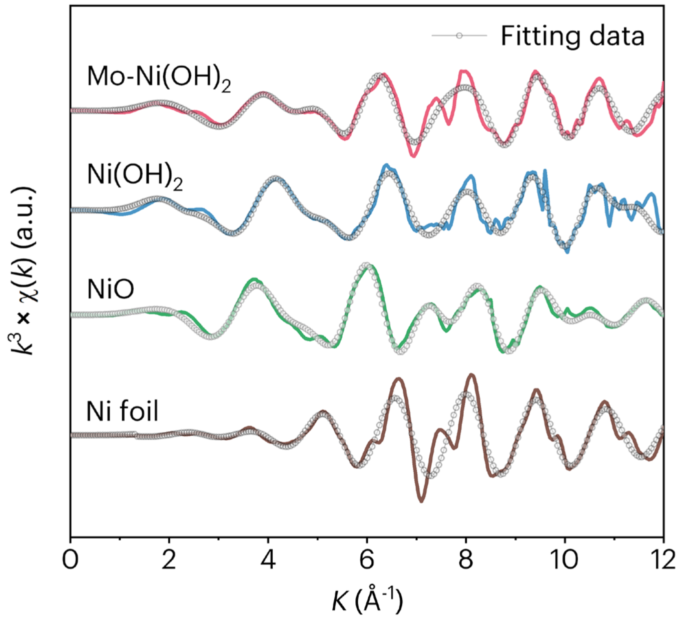

Fig. S20. *K*-space fitting. EXAFS spectra with fitting data in k^3-weighted *K*-space at the Ni K-edge for Mo-$Ni(OH)_2$, $Ni(OH)_2$, NiO and Ni foil.

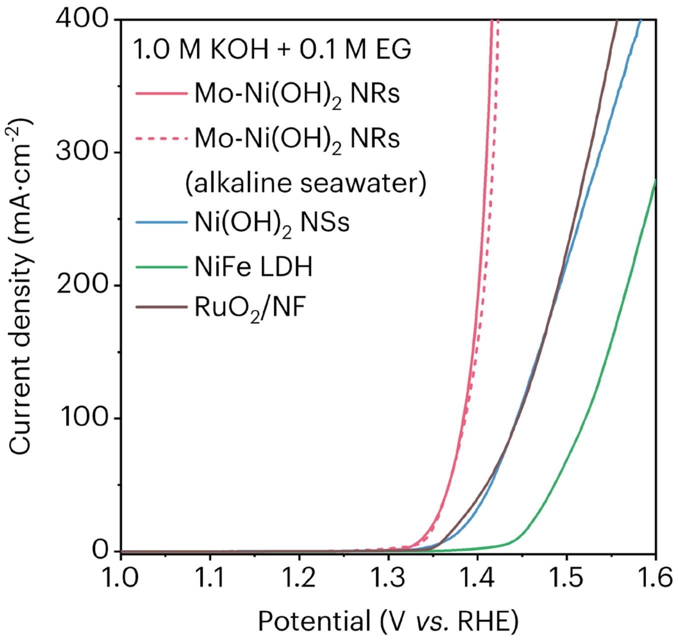

Fig. S21. EGOR activity. EGOR LSV curves of Mo-$Ni(OH)_2$ NRs, $Ni(OH)_2$ NSs, NiFe LDH and RuO_2/NF in 1.0 M KOH/alkaline seawater with 0.1 M EG.

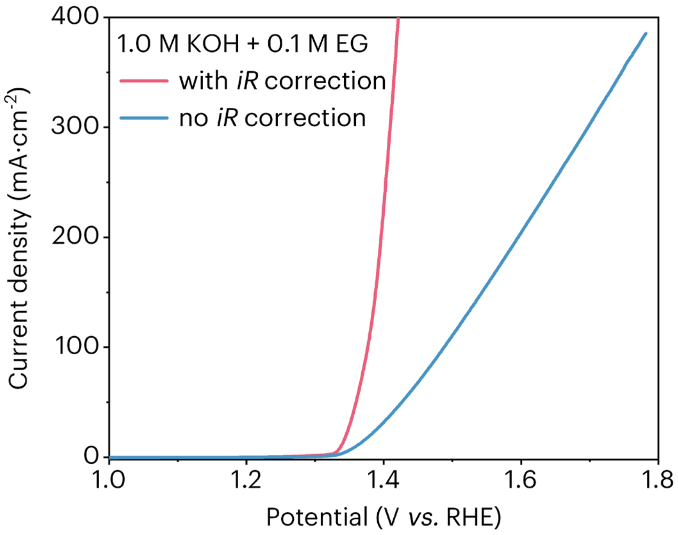

Fig. S22. EGOR activity. EGOR LSV curves of Mo-Ni$(OH)_2$ NRs in the solution of 1.0 M KOH with 0.1 M EG with/without *iR* correction.

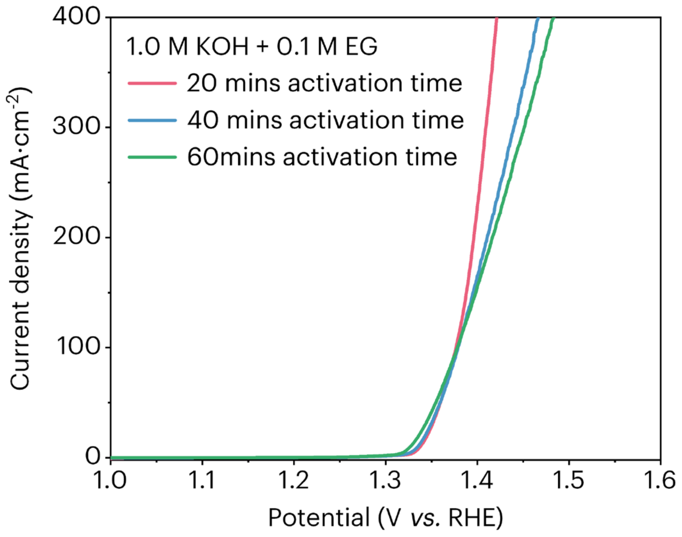

Fig. S23. EGOR activity. EGOR LSV curves of Mo-Ni$(OH)_2$ NRs under different electro-oxidation times.

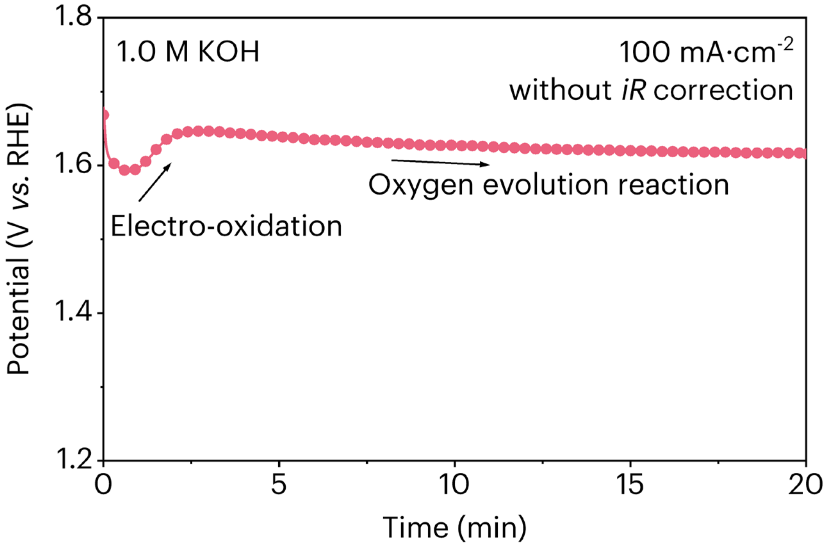

Fig. S24. Activation curve. Chronopotentiometry test for Mo-NiOOH NRs in the solution of 1.0 M KOH.

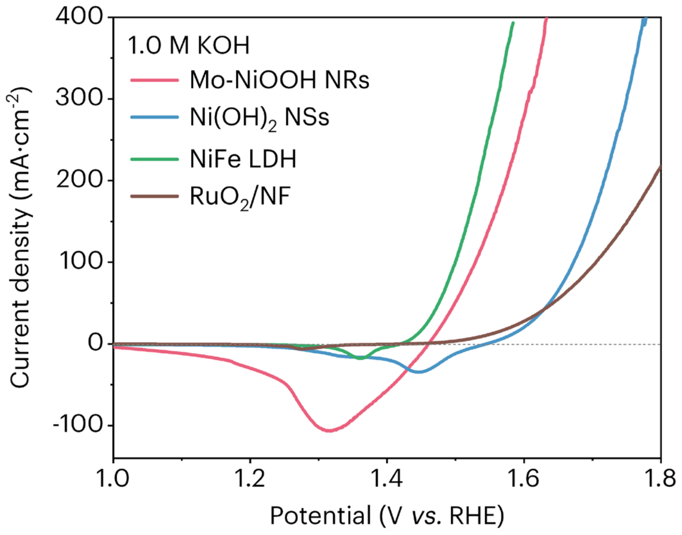

Fig. S25. OER activity. OER LSV curves of Mo-NiOOH NRs, $Ni(OH)_2$ NSs, NiFe LDH and RuO_2/NF in the solution of 1.0 M KOH.

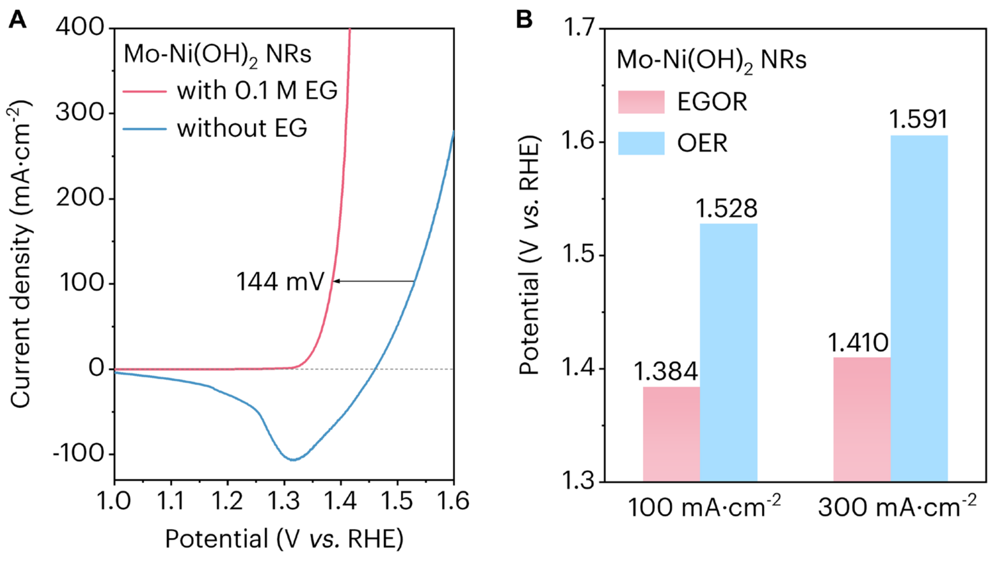

Fig. S26. Activity comparison. The comparisons of (**A**) LSV curves and (**B**) potentials for driving current densities of 100 and 300 mA·cm^{-2} of EGOR and OER.

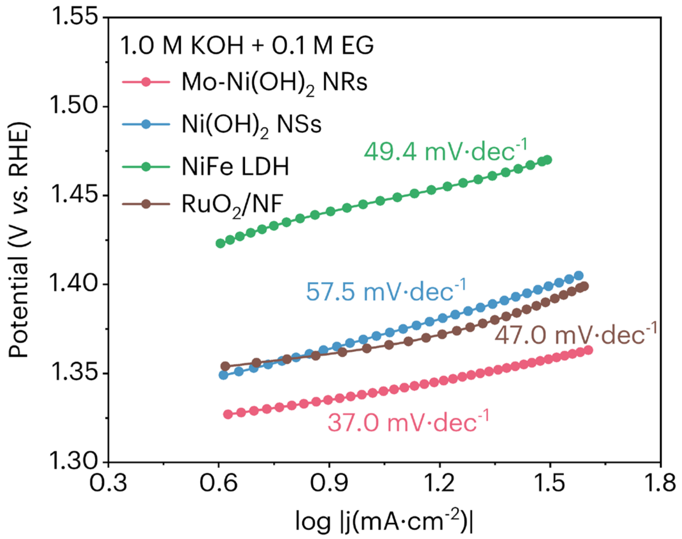

Fig. S27. EGOR activity. Tafel plots of Mo-Ni$(OH)_2$ NRs, Ni$(OH)_2$ NSs, NiFe LDH and RuO_2/NF for EGOR.

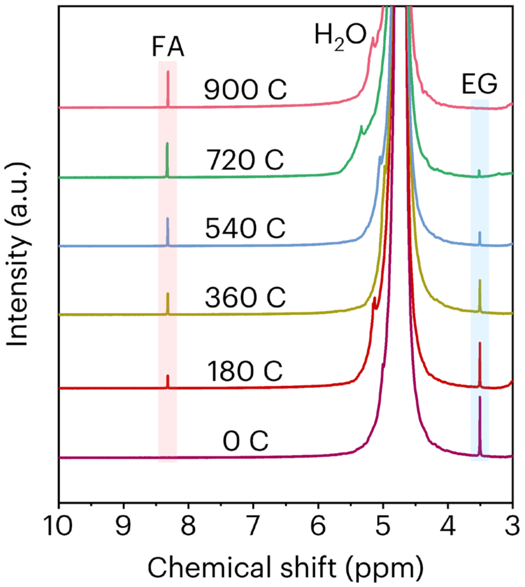

Fig. S28. ^{1}H NMR analysis. ^{1}H nuclear magnetic resonance (NMR) spectra in 1.0 M KOH with 10 mM EG after various electrolysis charges.

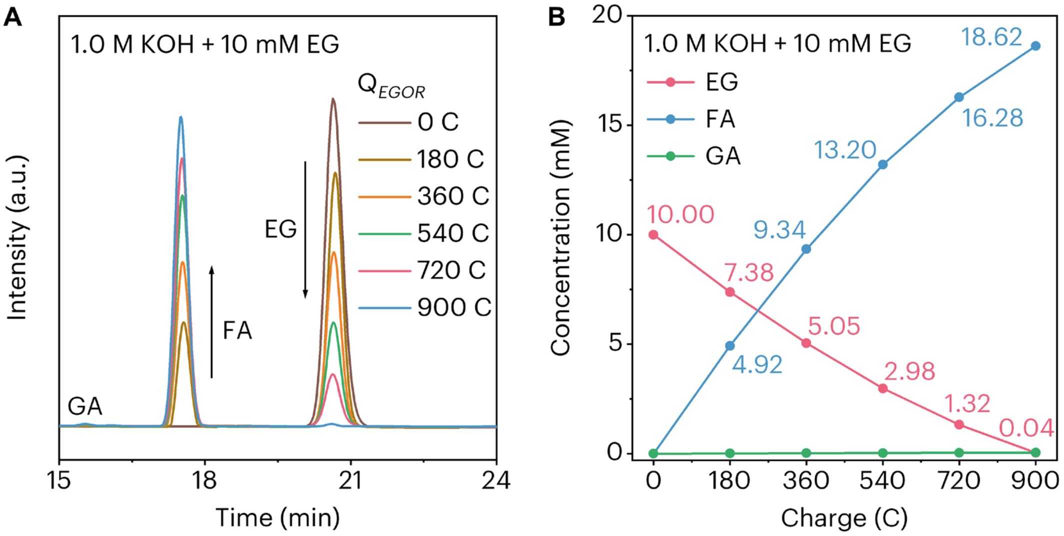

Fig. S29. HPLC analysis. (**A**) High-performance liquid chromatography (HPLC) chromatograms obtained in different concentrations of GA, FA and EG. (**B**) HPLC standard curve measurements of different concentrations of GA, FA and EG.

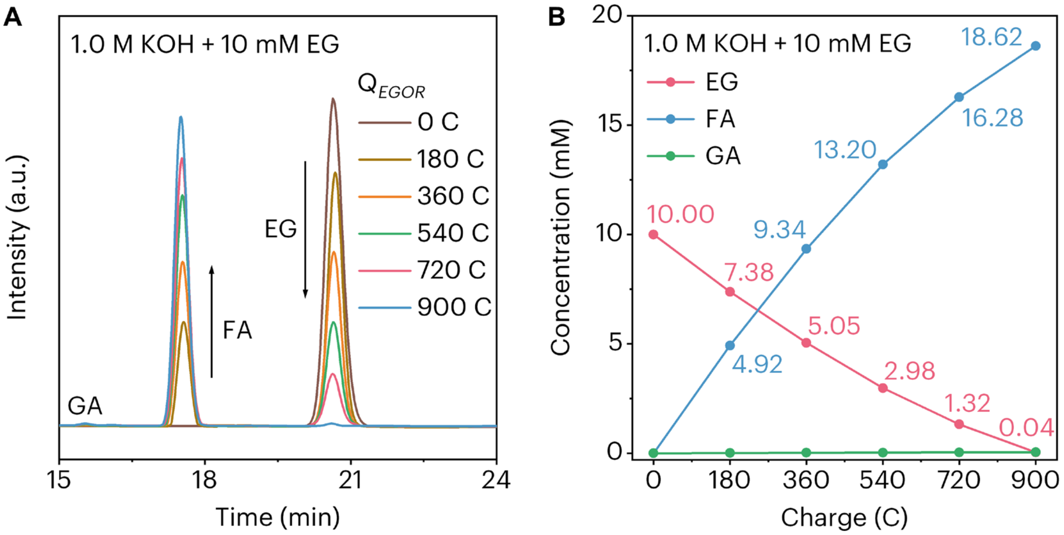

Fig. S30. HPLC analysis. (**A**) HPLC chromatograms obtained in 1.0 M KOH with 10 mM EG after various electrolysis charges during EGOR. (**B**) Concentrations of EG, FA and GA in 1.0 M KOH with 10 mM EG after various electrolysis charges during EGOR.

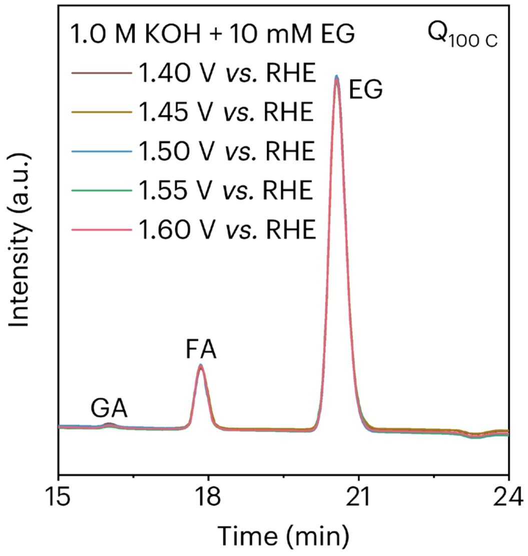

Fig. S31. HPLC analysis. HPLC chromatograms obtained in 1.0 M KOH with 10 mM EG after 100 C electrolysis charges during EGOR at different potentials.

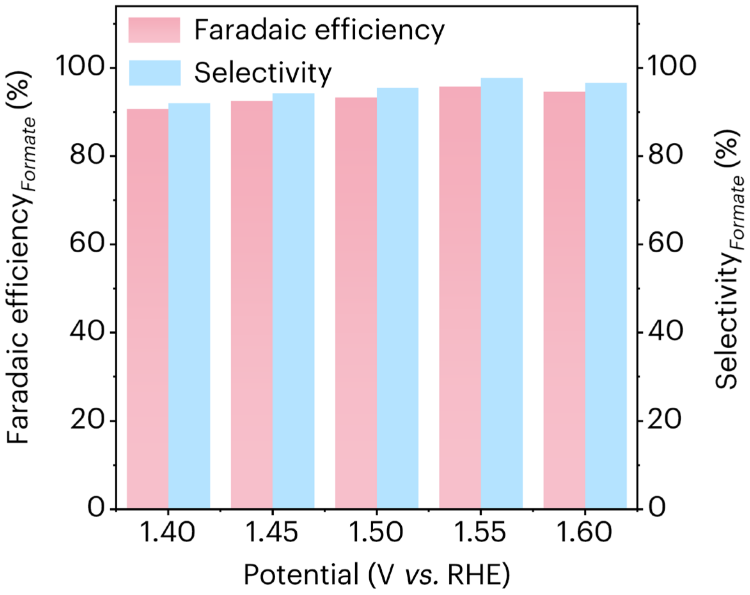

Fig. S32. Product analysis. Faradaic efficiency and selectivity of formate at different potentials.

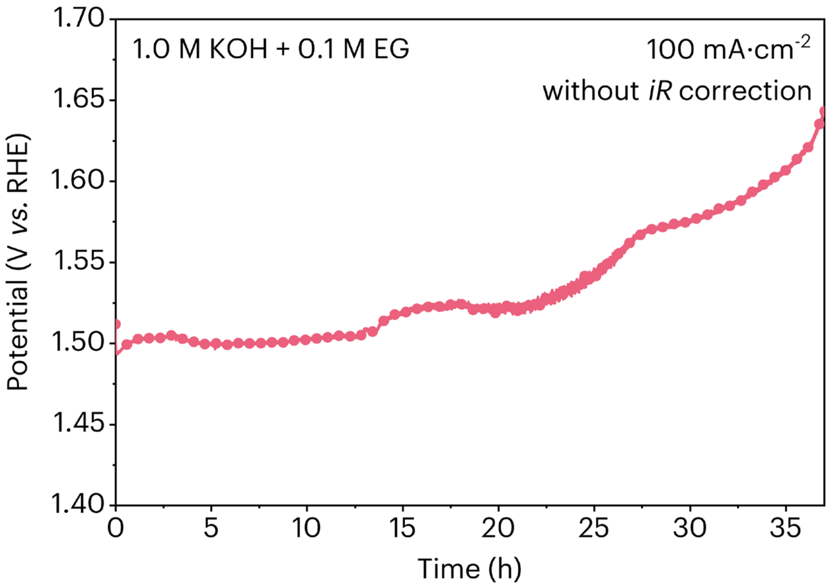

Fig. S33. EGOR stability analysis. Chronopotentiometry test for Mo-Ni(OH)$_2$ NRs in the solution of alkaline seawater with 0.1 M EG at the 150 mL beaker.

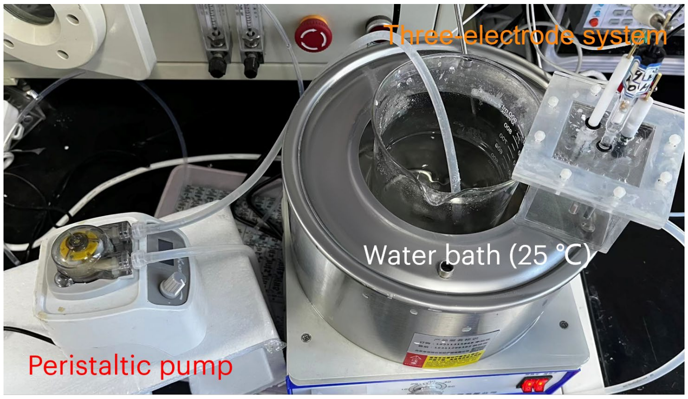

Fig. S34. System image. Digital image of the home-made flow electrolyzer for EGOR.

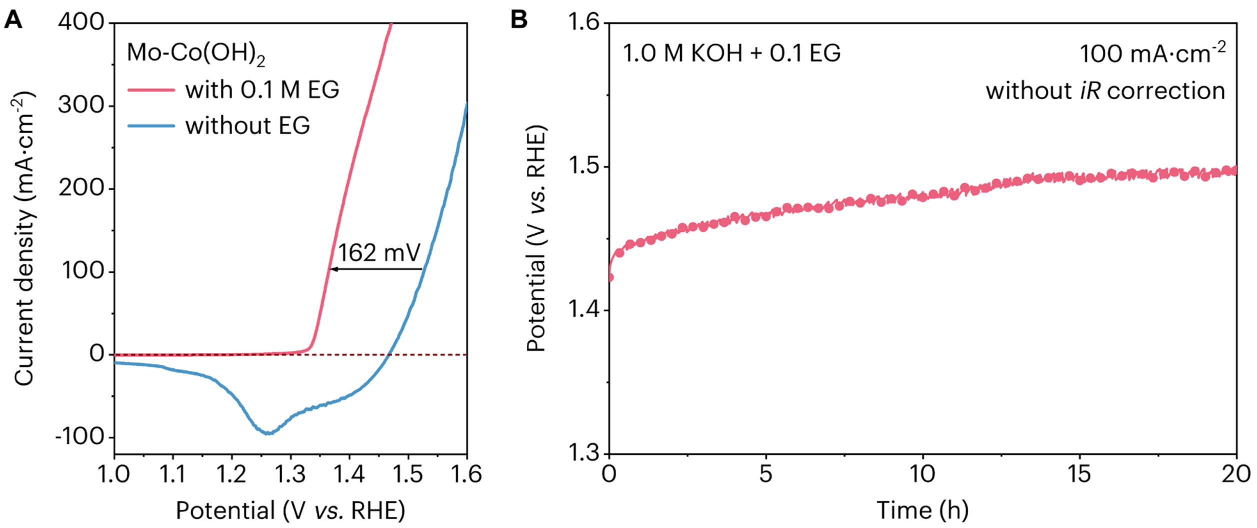

Fig. S35. EGOR activity. (**A**) The comparison of EGOR and OER (negative scanning direction) LSV curves for Mo-Co(OH)$_2$. (**B**) Chronopotentiometry test for Mo-Co(OH)$_2$ in the solution of 1.0 M KOH with 0.1 M EG (without *iR* correction).

Note: $Ni(NO_3)_2·6H_2O$ in raw material was replaced by $Co(NO_3)_2·6H_2O$ and other prepared methods were same.

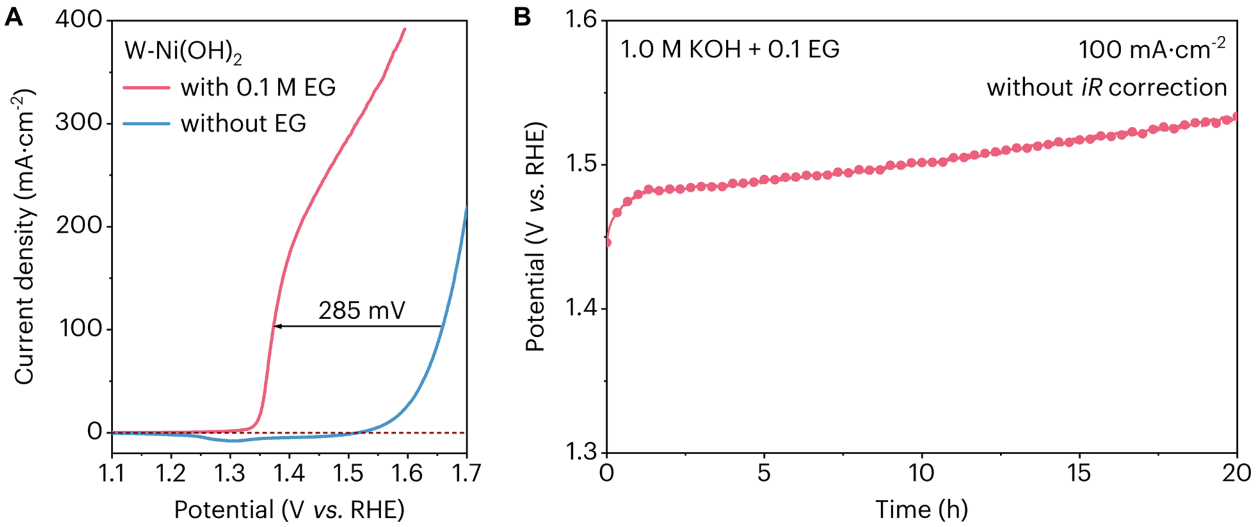

Fig. S36. EGOR activity. (**A**) The comparison of EGOR and OER (negative scanning direction) LSV curves for W-Ni$(OH)_2$. (**B**) Chronopotentiometry test for W-Ni$(OH)_2$ in the solution of 1.0 M KOH with 0.1 M EG (without *iR* correction).

Note: $NiWO_4 \cdot xH_2O$ precursor was prepared according to the previous report, (*52*) and other prepared methods were same.

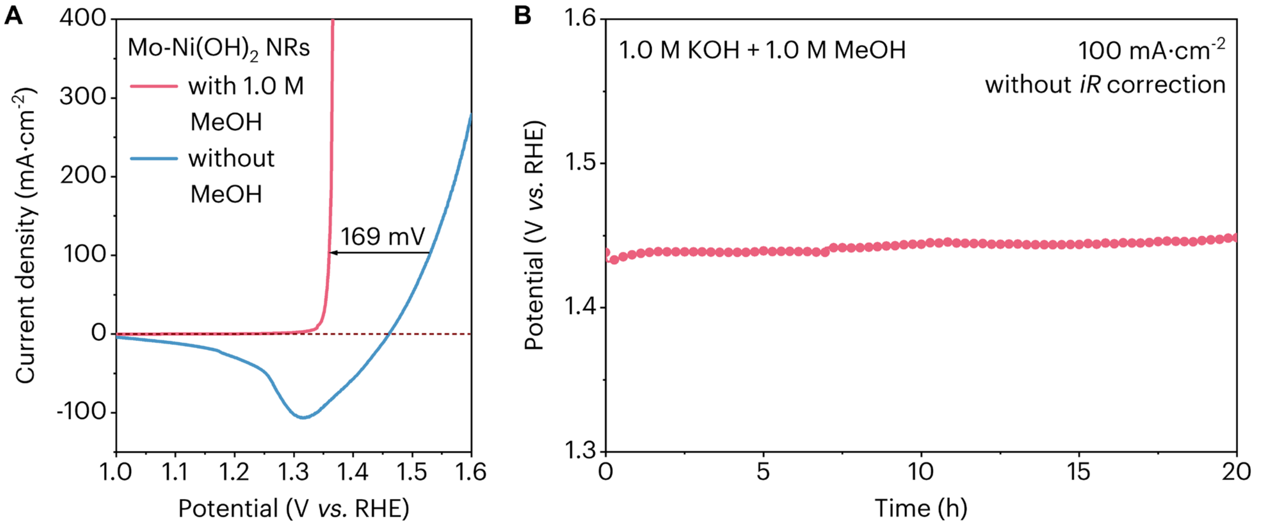

Fig. S37. Activity of methanol oxidation. (**A**) The comparison of methanol oxidation and OER (negative scanning direction) LSV curves for Mo-Ni$(OH)_2$ NRs. (**B**) Chronopotentiometry test for Mo-Ni$(OH)_2$ NRs in the solution of 1.0 M KOH with 1.0 M methanol (without *iR* correction).

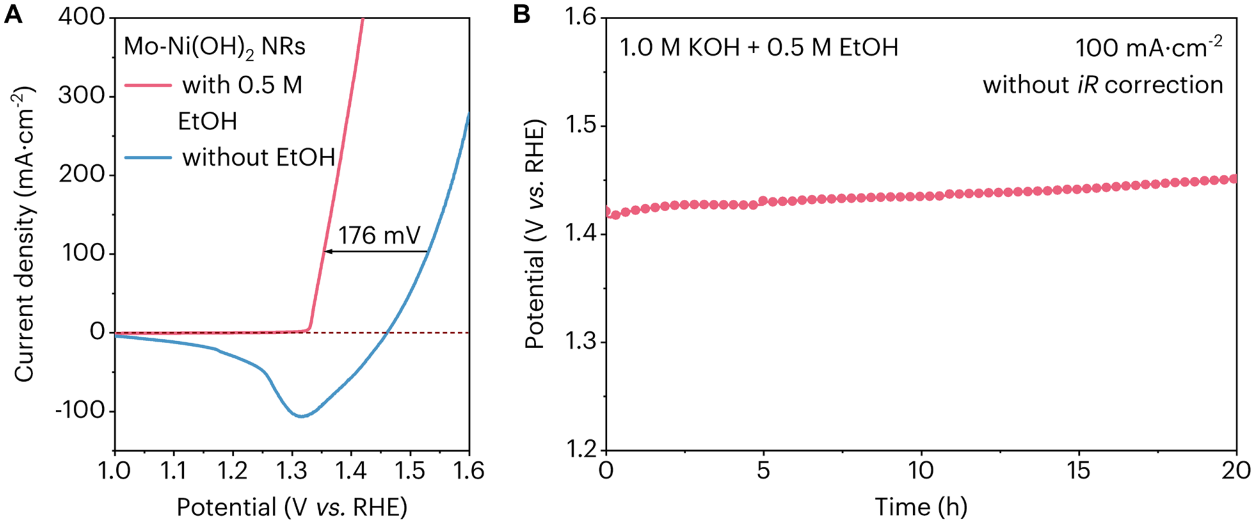

Fig. S38. Activity of ethanol oxidation. (**A**) The comparison of ethanol oxidation and OER (negative scanning direction) LSV curves for Mo-Ni$(OH)_2$ NRs. (**B**) Chronopotentiometry test for Mo-Ni$(OH)_2$ NRs in the solution of 1.0 M KOH with 0.5 M ethanol (without *iR* correction).

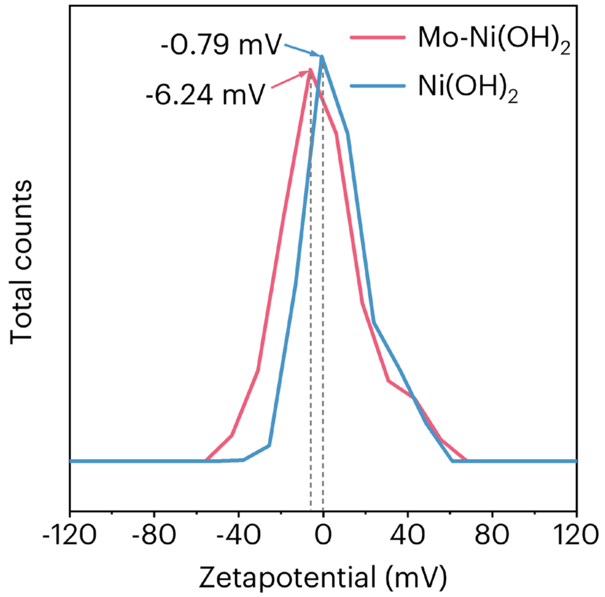

Fig. S39. Zeta potential analysis. Zeta potentials of Mo-$Ni(OH)_2$ NRs and $Ni(OH)_2$ NSs.

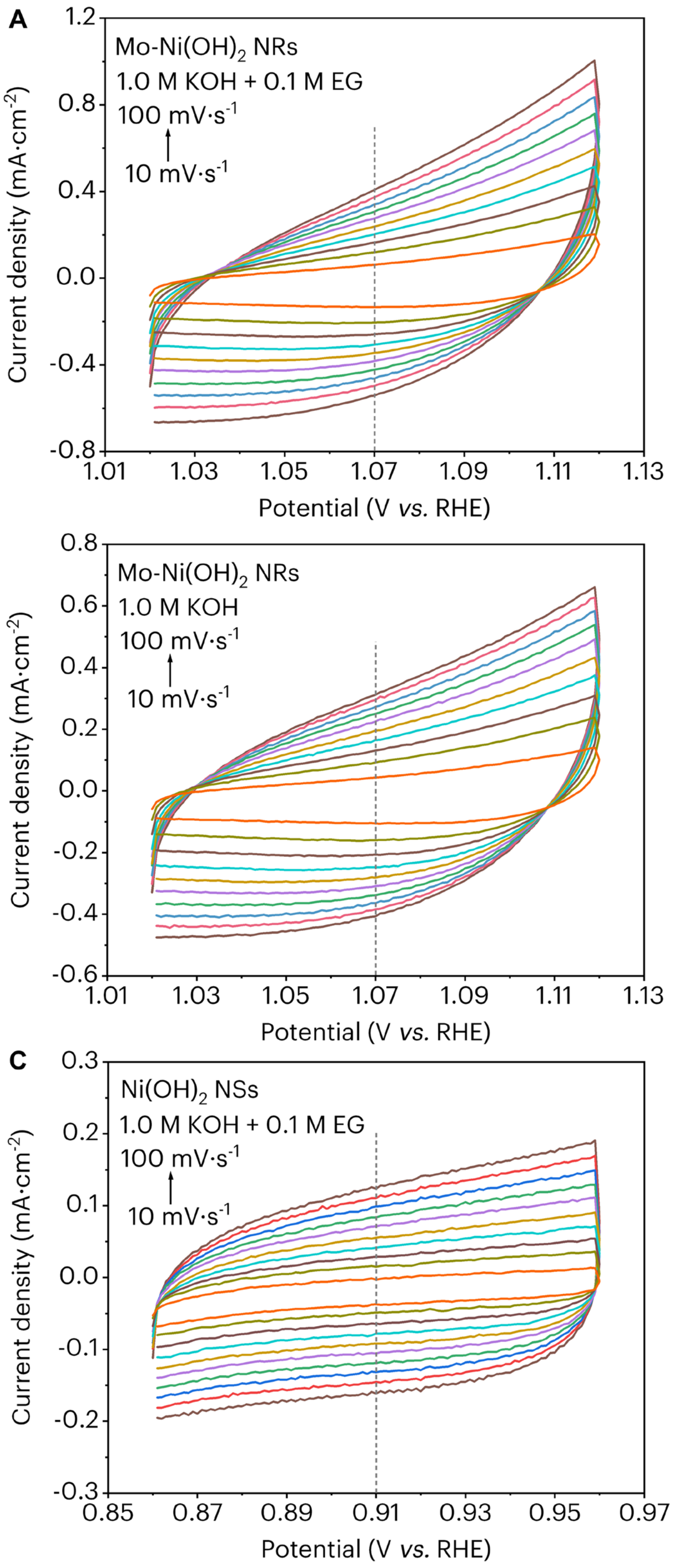

Fig. S40. CV measurement. CVs of Mo-$Ni(OH)_2$ NRs for (**A**) EGOR and (**B**) OER, and (**C**) CVs of $Ni(OH)_2$ NSs for EGOR under different scan rates in the non-Faradaic region.

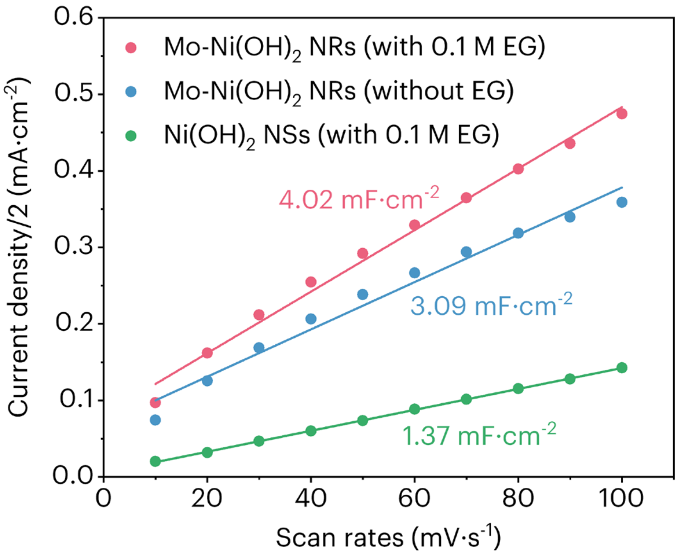

Fig. S41. C_{dl} analysis. Double-layer capacitance (C_{dl}) values of Mo-$Ni(OH)_2$ NRs and $Ni(OH)_2$ NSs for EGOR, and Mo-$Ni(OH)_2$ NRs for OER.

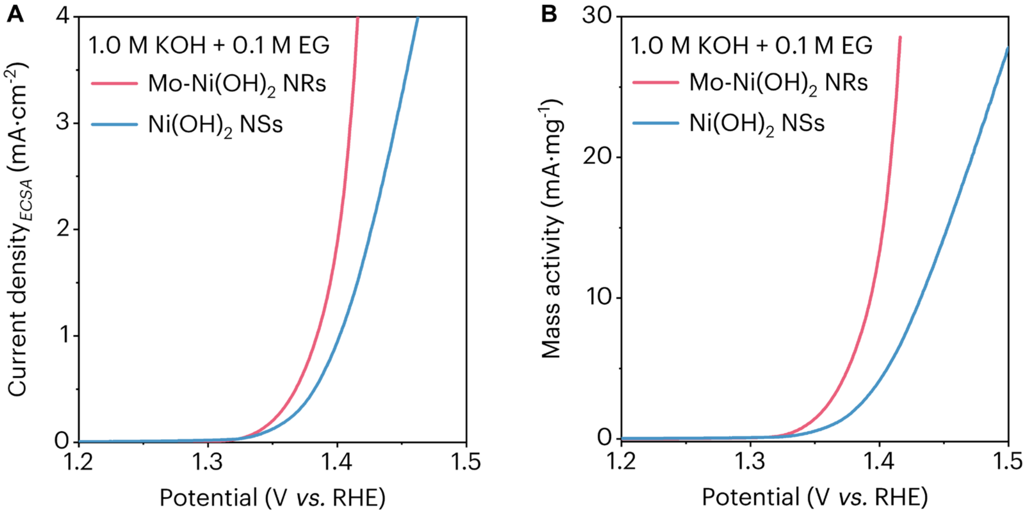

Fig. S42. EGOR activity. (**A**) EGOR LSV curves of Mo-$Ni(OH)_2$ NRs and $Ni(OH)_2$ NSs with ECSA correction. (**B**) EGOR LSV curves of Mo-$Ni(OH)_2$ NRs and $Ni(OH)_2$ NSs with mass loading correction.

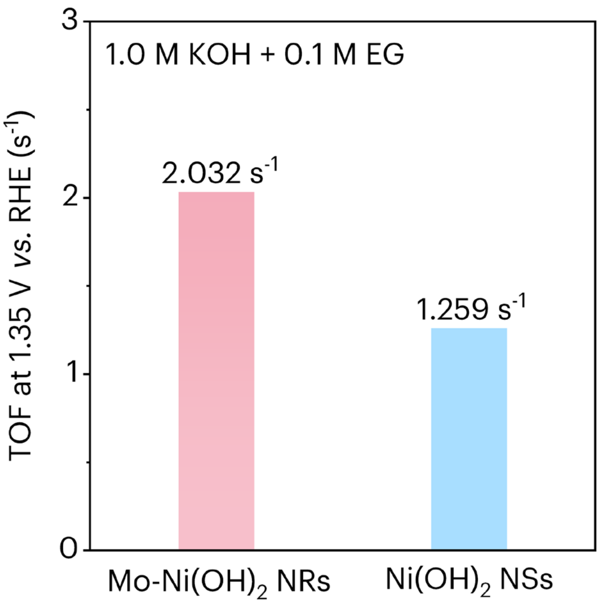

Fig. S43. TOF analysis. TOF values of Mo-$Ni(OH)_2$ NRs and $Ni(OH)_2$ NSs at 1.35 V *vs.* RHE.

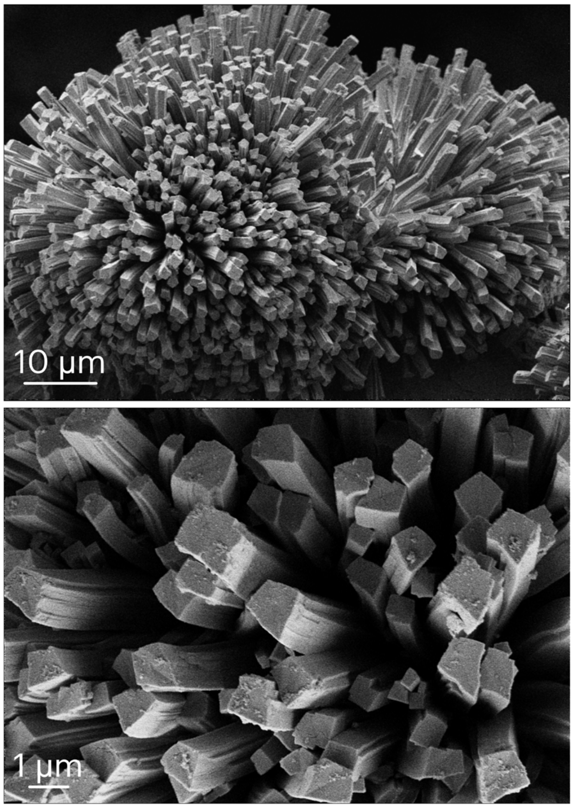

Fig. S44. Structural analysis. SEM images of Mo-$Ni(OH)_2$ NRs post EGOR stability test.

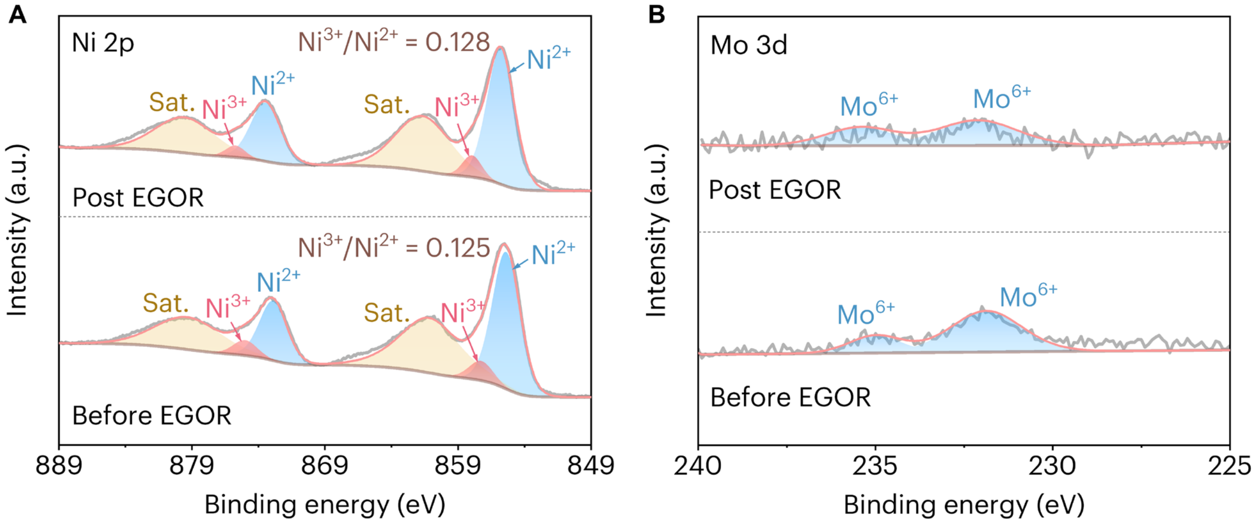

Fig. S45. XPS characterization. High-resolution (**A**) Ni 2p and (**B**) Mo 3d XPS spectra of Mo-Ni(OH)$_2$ NRs before and post EGOR stability test.

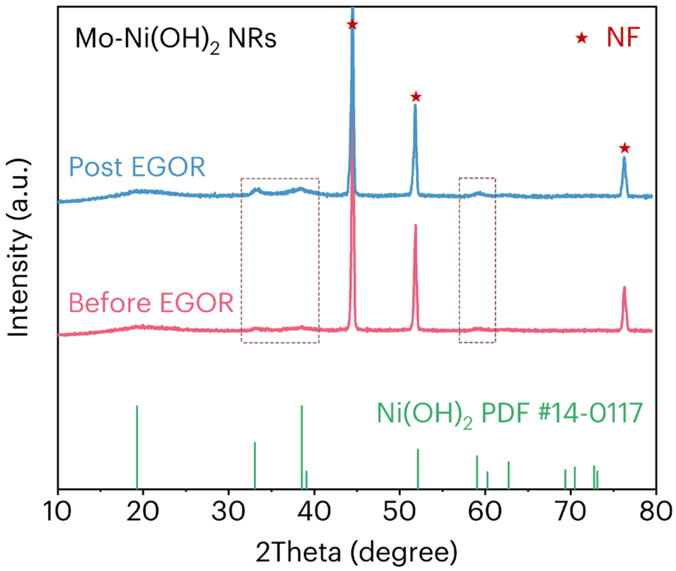

Fig. S46. Structural analysis. XRD patterns of Mo-$Ni(OH)_2$ NRs before and post EGOR stability test.

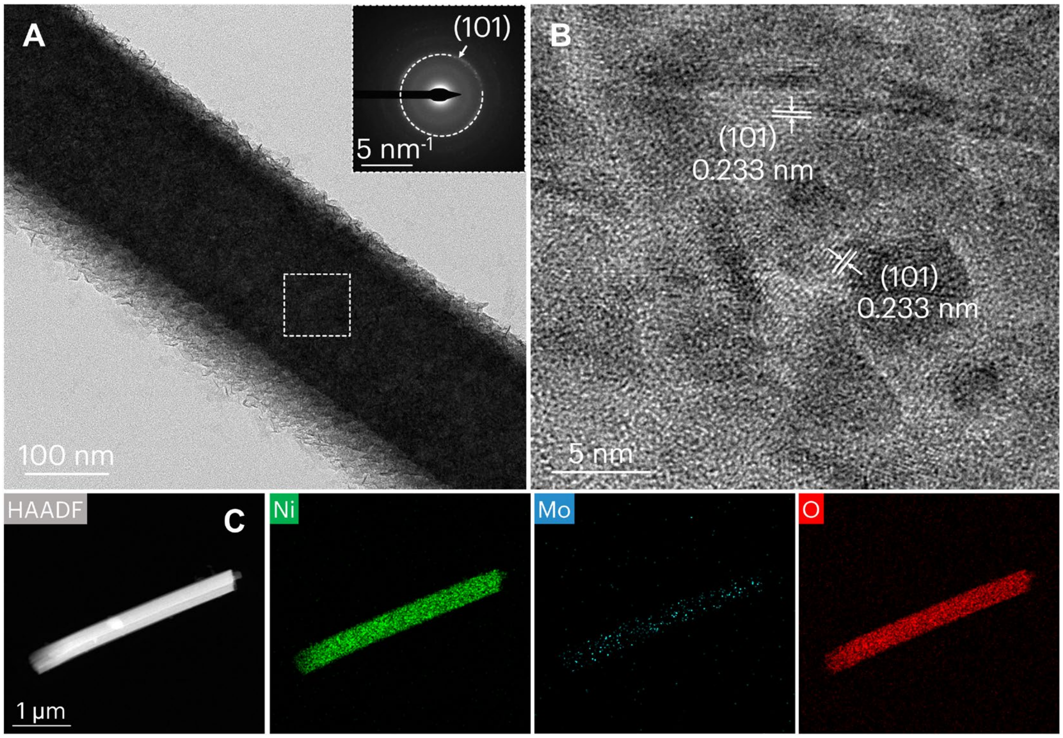

Fig. S47. Structural analysis. (**A**) TEM image with corresponding SAED pattern of Mo-Ni(OH)$_2$ NRs post EGOR stability test. (**B**) HR-TEM image of Mo-Ni(OH)$_2$ NRs post EGOR stability test. (**C**) The energy-dispersive spectroscopy (EDS) mapping images of Ni, Mo and O of Mo-Ni(OH)$_2$ NRs post EGOR stability test.

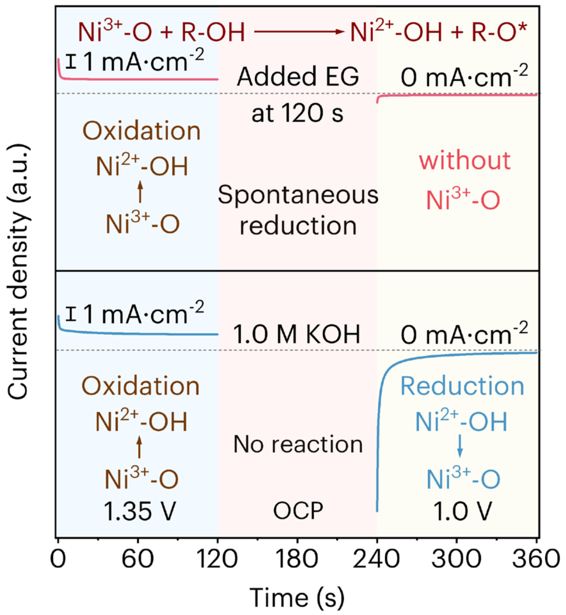

Fig. S48. EGOR mechanism analysis. Multi-potential step measurements of Mo-$Ni(OH)_2$.

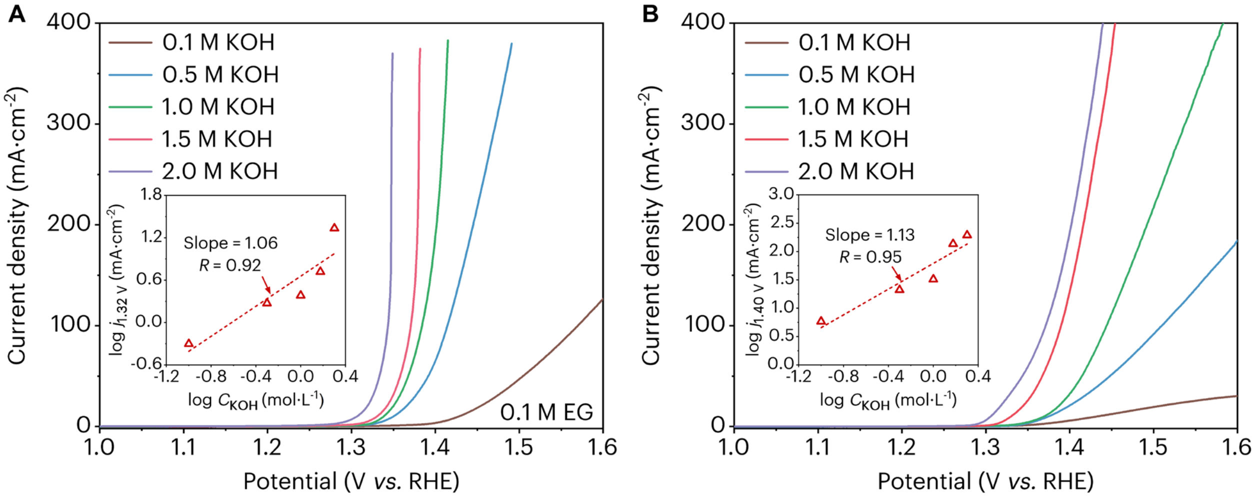

Fig. S49. EGOR mechanism analysis. LSV curves for (**A**) Mo-$Ni(OH)_2$ NRs and (**B**) $Ni(OH)_2$ NSs in different concentrations of KOH with 0.1 M EG. Inset: the liner dependence of current density with various KOH concentrations.

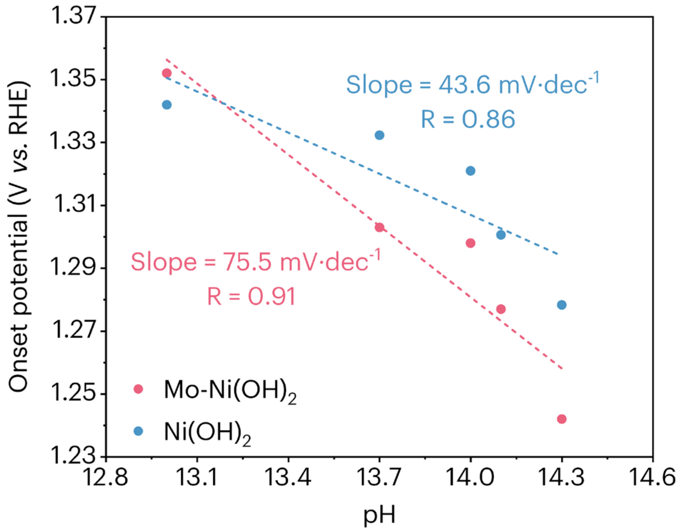

Fig. S50. EGOR mechanism analysis. Slope of pH with the onset potential (potential at the current density of 1 mA·cm^{-2}) on Mo-Ni$(OH)_2$ NRs.

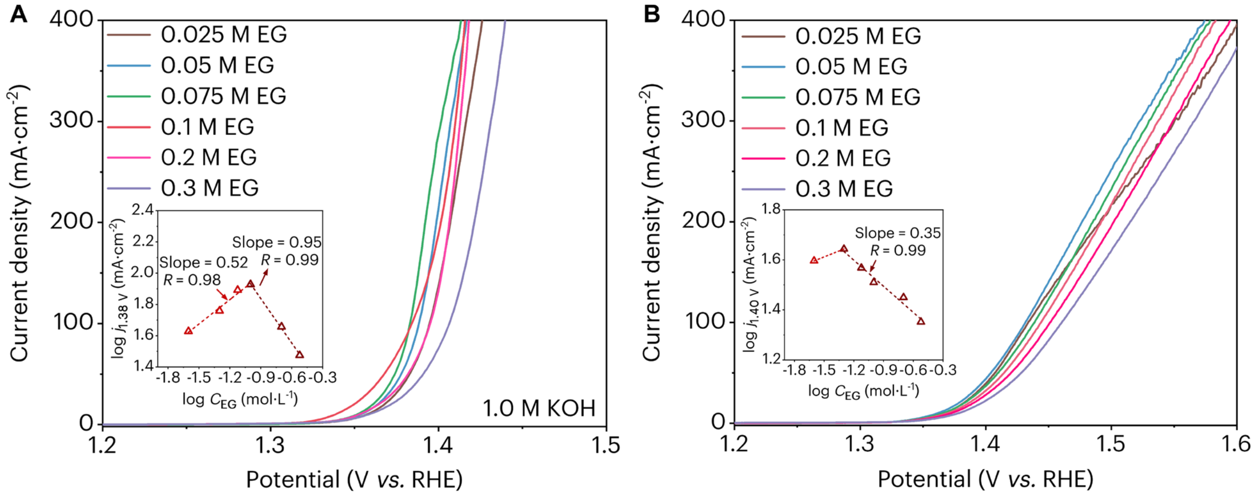

Fig. S51. EGOR mechanism analysis. LSV curves for (**A**) Mo-Ni(OH)$_2$ NRs and (**B**) Ni(OH)$_2$ NSs in different concentrations of EG with 1.0 M KOH. Inset: the liner dependence of current density with various EG concentrations.

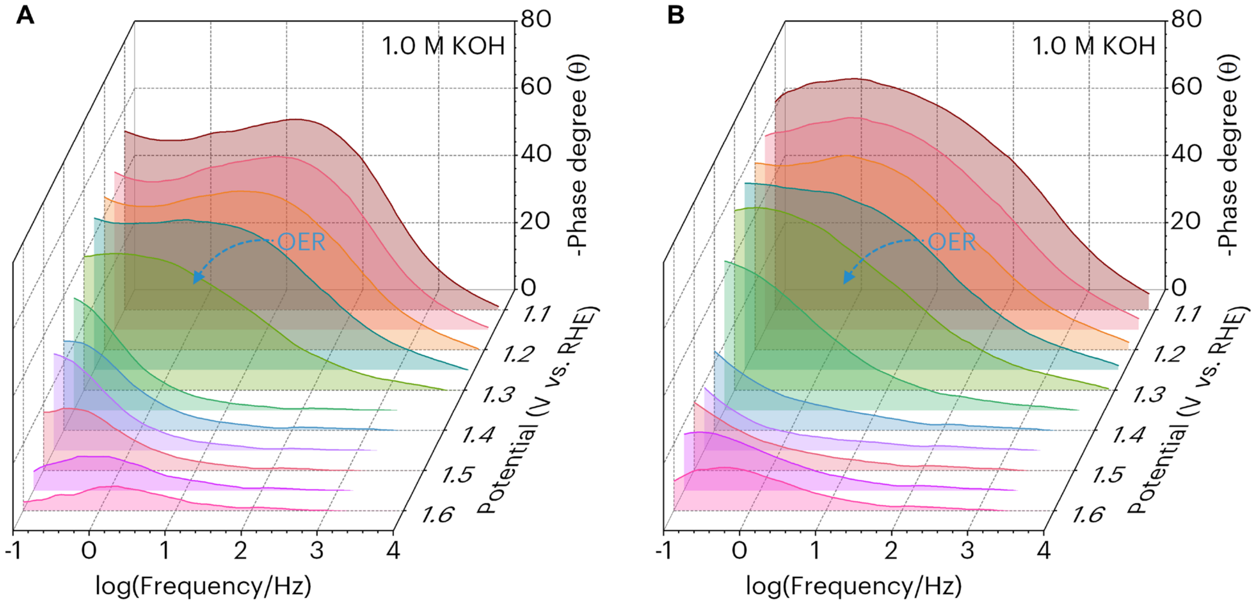

Fig. S52. Impedance analysis. *In-situ* Bode plots of (**A**) Mo-$Ni(OH)_2$ NRs and (**B**) $Ni(OH)_2$ NSs in 1.0 M KOH.

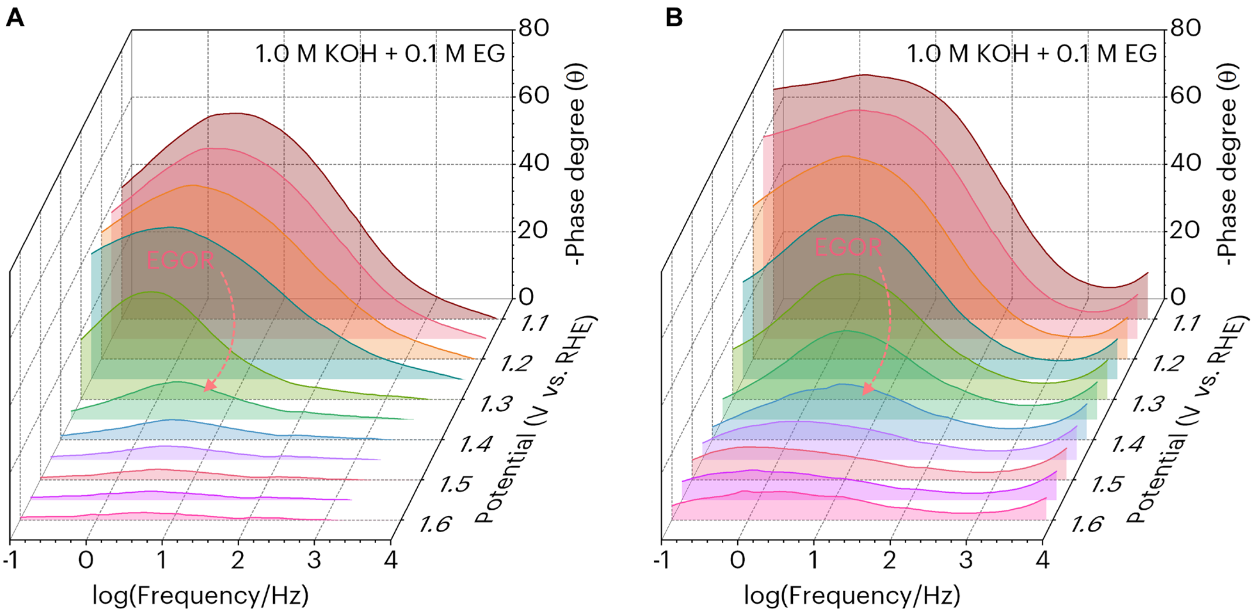

Fig. S53. Impedance analysis. *In-situ* Bode plots of (**A**) Mo-$Ni(OH)_2$ NRs and (**B**) $Ni(OH)_2$ NSs in 1.0 M KOH with 0.1 M EG.

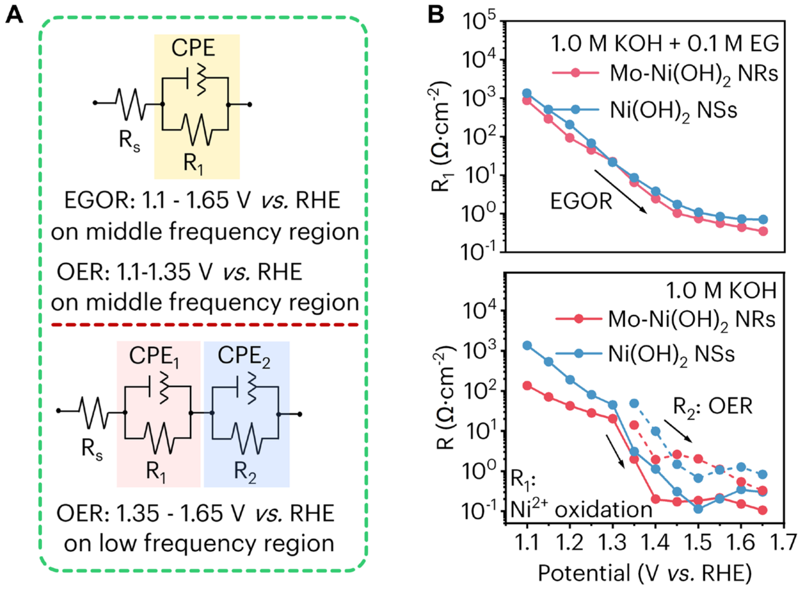

Fig. S54. Impedance analysis. (**A**) The equivalent circuits for fitting *In-situ* Bode plots with (**B**) corresponding resistance values.

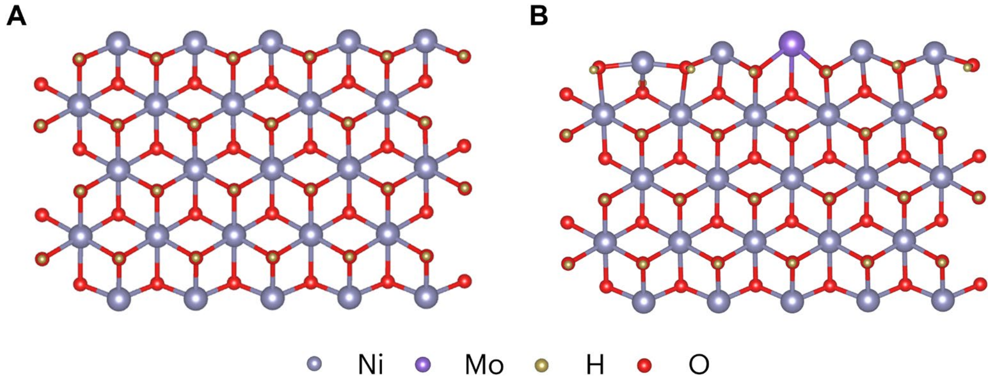

Fig. S55. Model diagram. DFT-optimized configurations of (**A**) $Ni(OH)_2$ (101) and (**B**) Mo-$Ni(OH)_2$ (101).

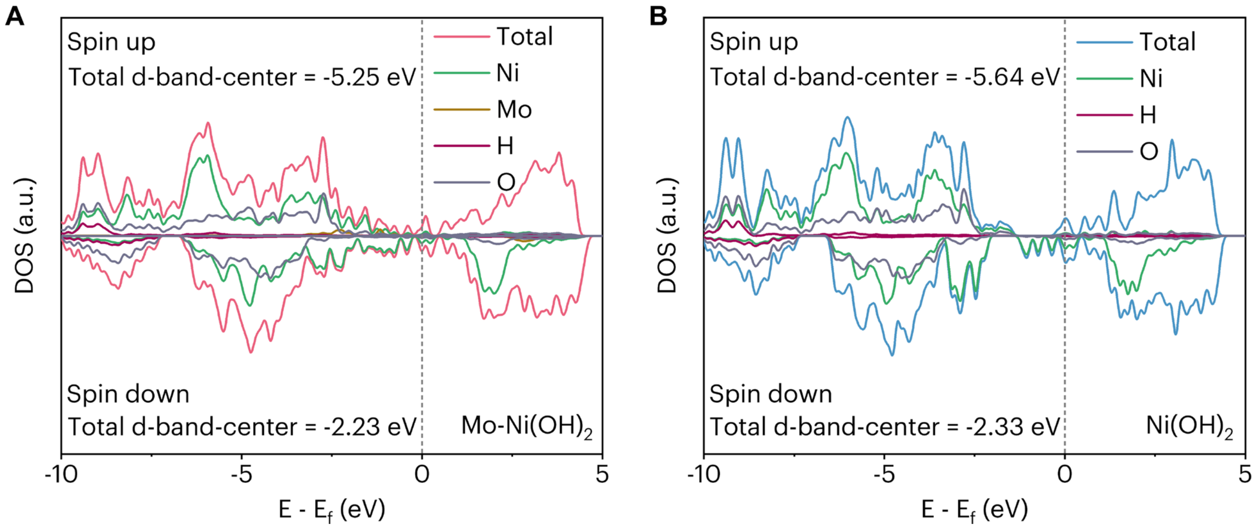

Fig. S56. DOS analysis. Density of states (DOS) analysis of d-orbitals for (**A**) Mo-Ni(OH)$_2$ (101) and (**B**) Ni(OH)$_2$ (101). Isosurface value is set to be 0.005 eV·Å^{-3}.

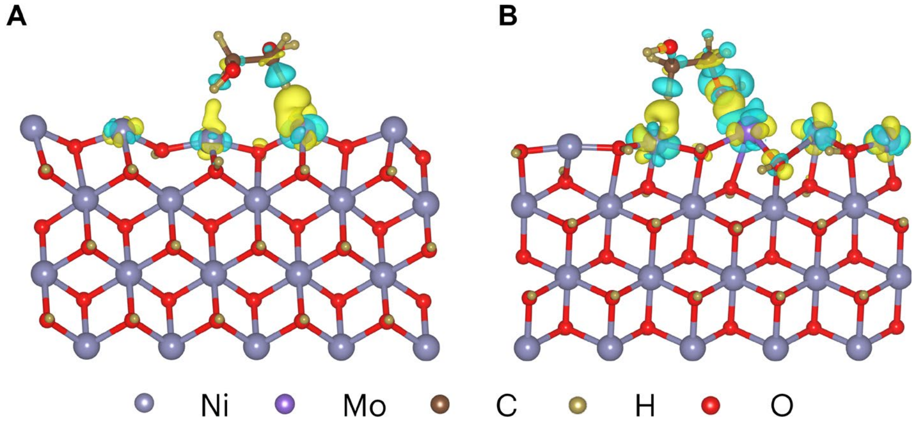

Fig. S57. Differential charge density distribution analysis. Differential charge density distributions of (**A**) EG-adsorbed $Ni(OH)_2$ (101) and (**B**) Mo-$Ni(OH)_2$ (101). Blue and yellow areas represent electron accumulation and depletion regions.

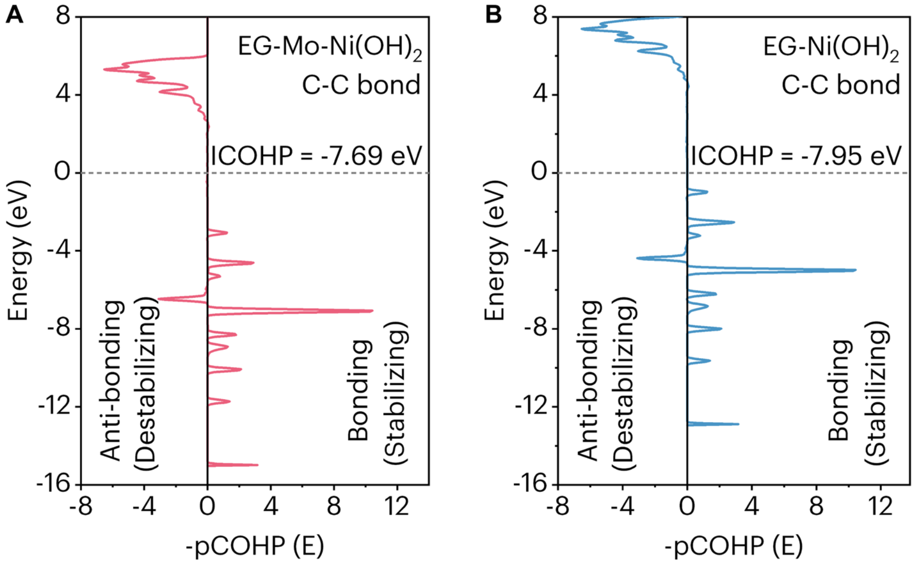

Fig. S58. COHP analysis. Crystal orbital Hamilton population (COHP) analysis for EG-adsorbed (**A**) Mo-$Ni(OH)_2$ (101) and (**B**) $Ni(OH)_2$ (101).

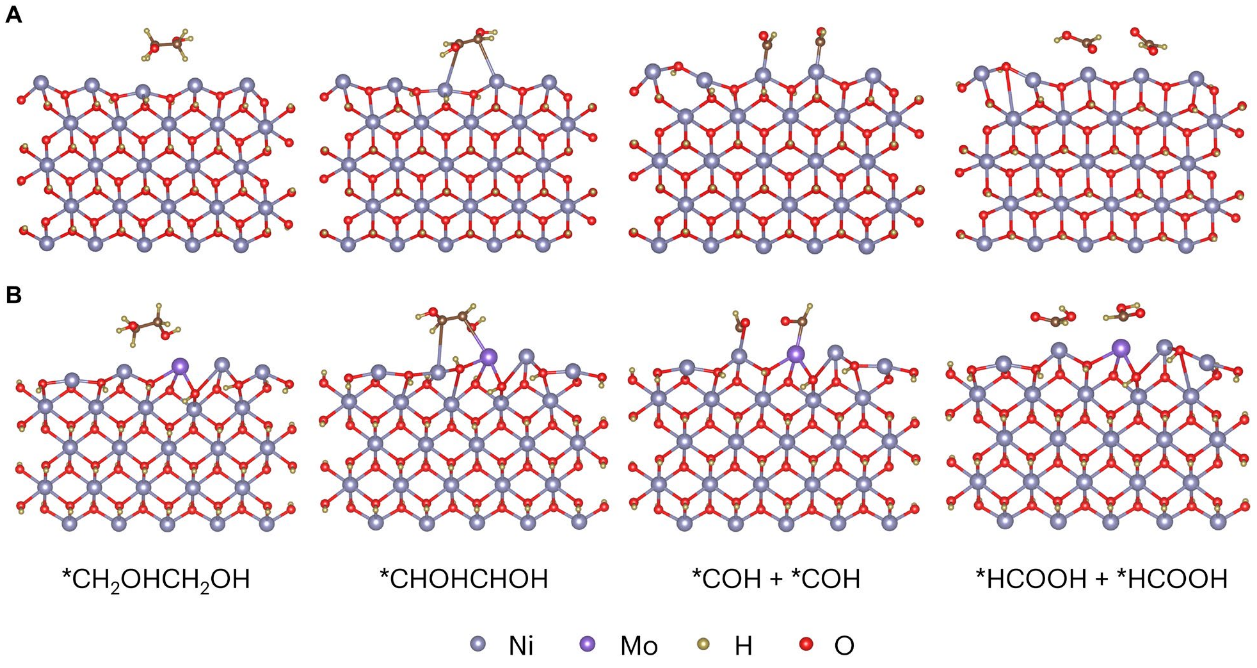

Fig. S59. Model diagram. DFT-optimized configurations of the adsorbed intermediates during EGOR on (**A**) $Ni(OH)_2$ (101) and (**B**) Mo-$Ni(OH)_2$ (101).

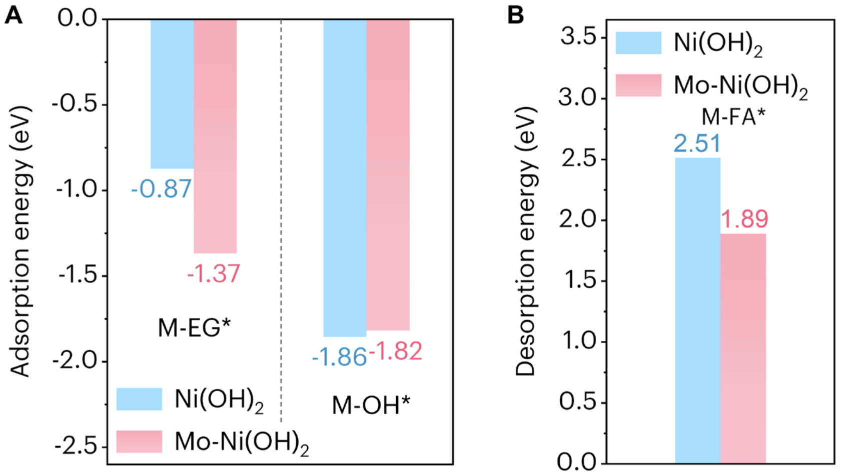

Fig. S60. Binding energy analysis. (**A**) EG and hydroxyl adsorption energy and (**B**) FA desorption energy for Mo-Ni$(OH)_2$ (101) and Ni$(OH)_2$ (101).

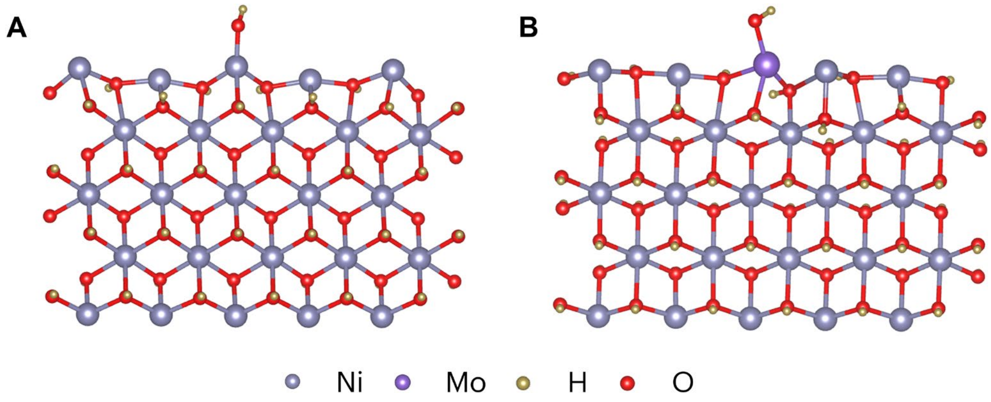

Fig. S61. Model diagram. DFT-optimized configurations of hydroxyl-adsorbed (**A**) $Ni(OH)_2$ (101) and (**B**) Mo-$Ni(OH)_2$ (101).

Reaction mechanism of FLPs for EGOR:

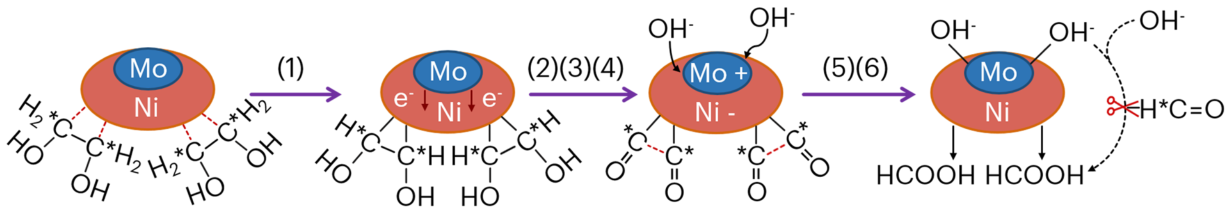

$$HOCH_2CH_2OH + 2OH^- \rightarrow HOC^*HC^*HOH_{ads} + 2H_2O + 2e^- \quad (1)$$

$$\text{FLPs: } Mo + Ni \rightarrow Mo(+)/Ni(-) \quad (2)$$

$$Mo(+) + OH^- \rightarrow [Mo(+)\text{-}OH]^- \quad (3)$$

$$HOC^*HC^*HOH_{ads} + 2Ni(-) + 2OH^- \rightarrow 2Ni(-)\text{-}C^*O + 2H_2O + 2e^- \quad (4)$$

$$2Ni(-)\text{-}C^*O + 2[Mo(+)\text{-}OH]^- \rightarrow 2HCOOH + 2Ni(-) + 2Mo(+) + 2e^- \quad (5)$$

$$Mo(+) + Ni(-) \rightarrow Mo + Ni \quad (6)$$

Fig. S62. EGOR mechanism diagram. Proposed synergetic catalytic mechanism of FLPs for EGOR.

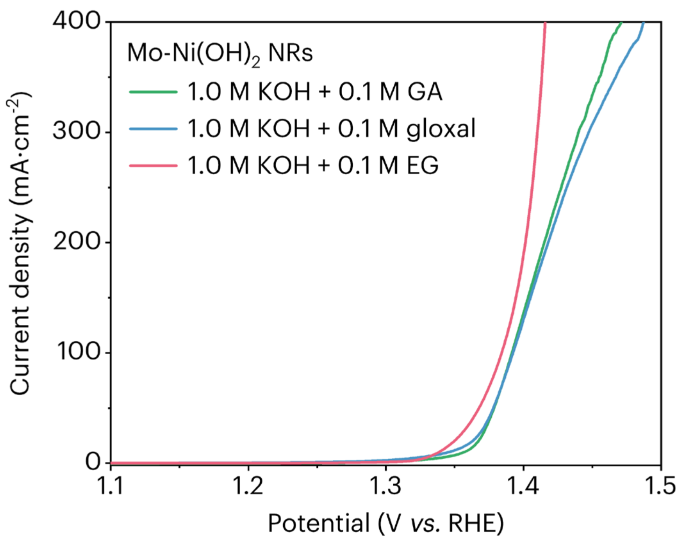

Fig. S63. Product analysis. LSV curves of Mo-$Ni(OH)_2$ NRs in 1.0 M KOH with 0.1 M GA, 0.1 M gloxal and 0.1 M EG.

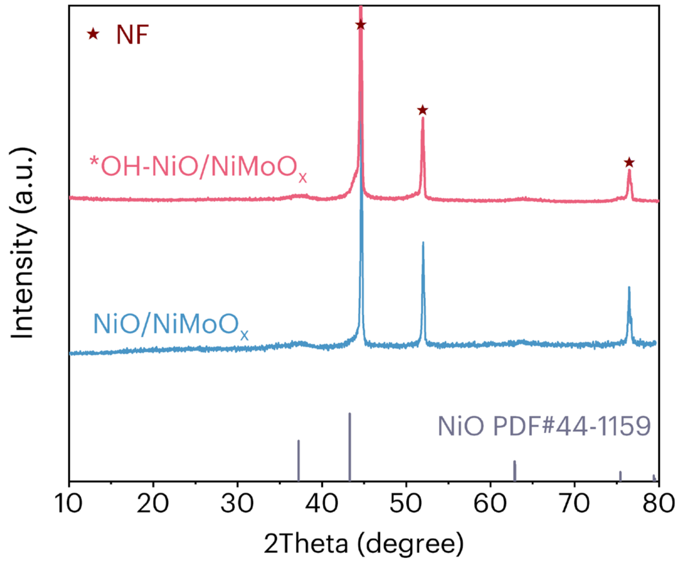

Fig. S64. Structural analysis. XRD patterns of *OH-NiO/NiMoO$_x$ (post HER stability test) and NiO/NiMoO$_x$ (before HER stability test).

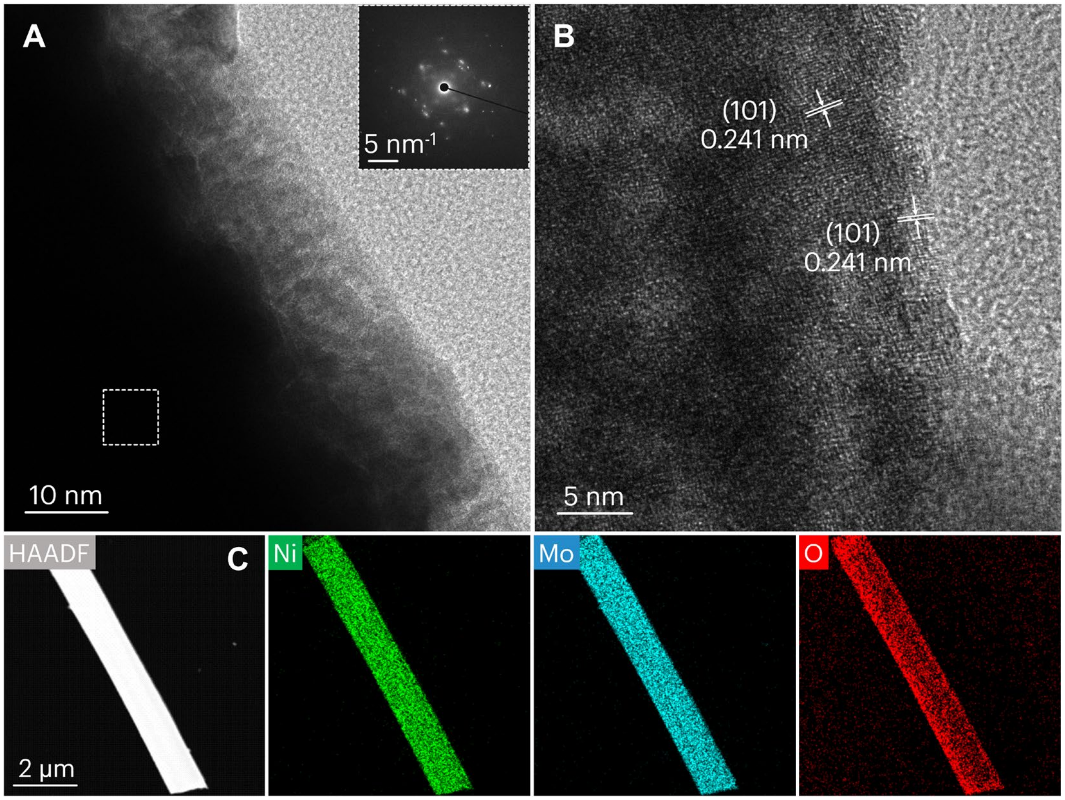

Fig. S65. Structural analysis. (**A**) TEM image with corresponding SAED pattern of NiO/NiMoO$_x$. (**B**) HR-TEM image of NiO/NiMoO$_x$. (**C**) The EDS elemental mapping images of Ni, Mo and O for NiO/NiMoO$_x$.

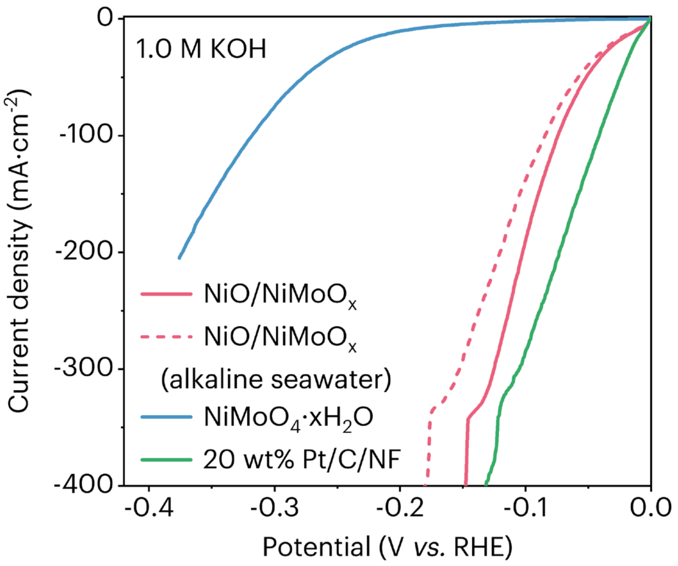

Fig. S66. HER activity. HER LSV curves of NiO/NiMoO$_x$, NiMoO$_4$·xH$_2$O and 20 wt% Pt/C/NF in 1.0 M KOH/alkaline seawater.

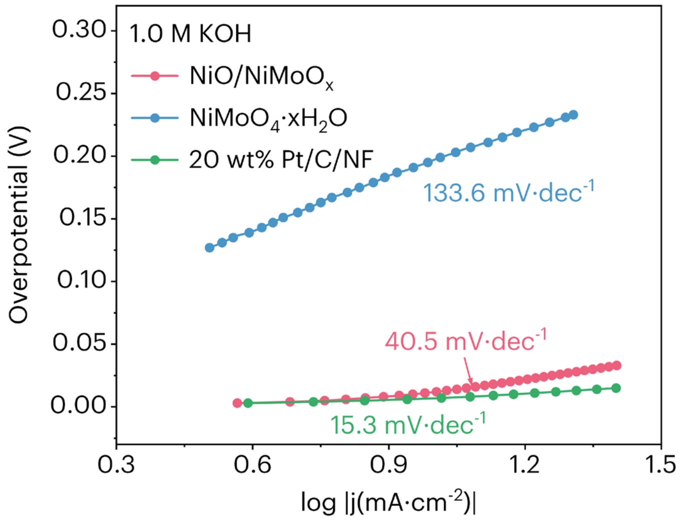

Fig. S67. HER activity. Tafel plots of NiO/$NiMoO_x$, $NiMoO_4 \cdot xH_2O$ and 20 wt% Pt/C/NF for HER.

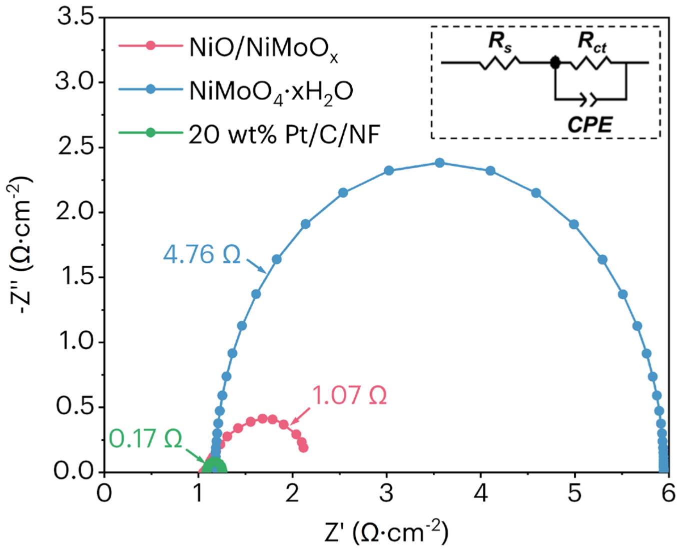

Fig. S68. Impedance analysis. EIS plots of NiO/NiMoO$_x$, NiMoO$_4$·xH$_2$O and 20 wt% Pt/C/NF for HER at the current density of 10 mA·cm^{-2}. Inset shows the fitting circuit diagram.

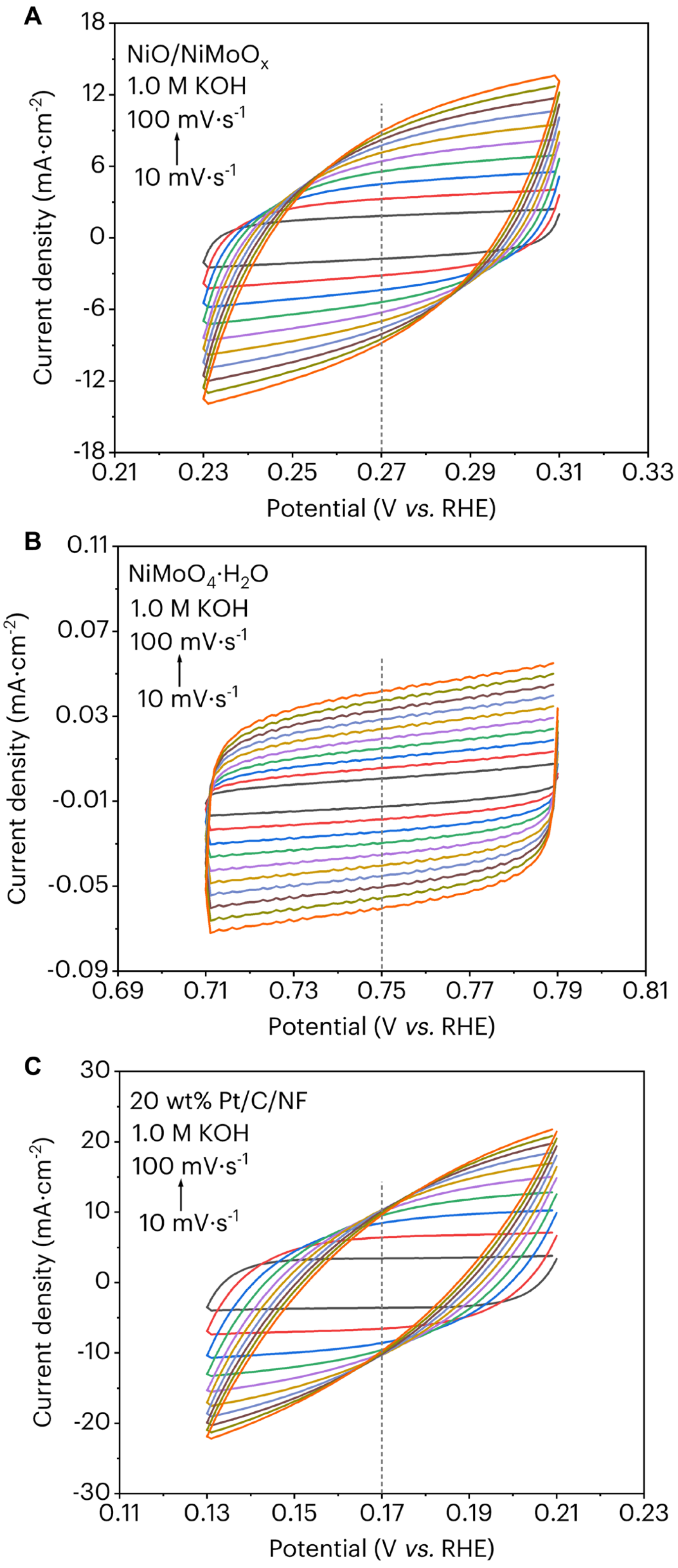

Fig. S69. CV measurement. CVs of (**A**) NiO/NiMoO$_x$, (**B**) $NiMoO_4 \cdot xH_2O$ and (**C**) 20 wt% Pt/C/NF for HER under different scan rates in the non-Faradaic region.

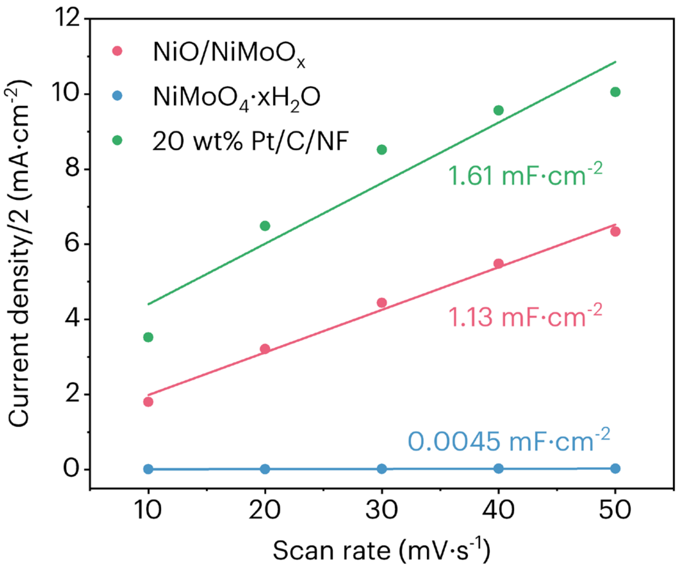

Fig. S70. C_{dl} analysis. C_{dl} values of $NiO/NiMoO_x$, $NiMoO_4 \cdot xH_2O$ and 20 wt% Pt/C/NF for HER.

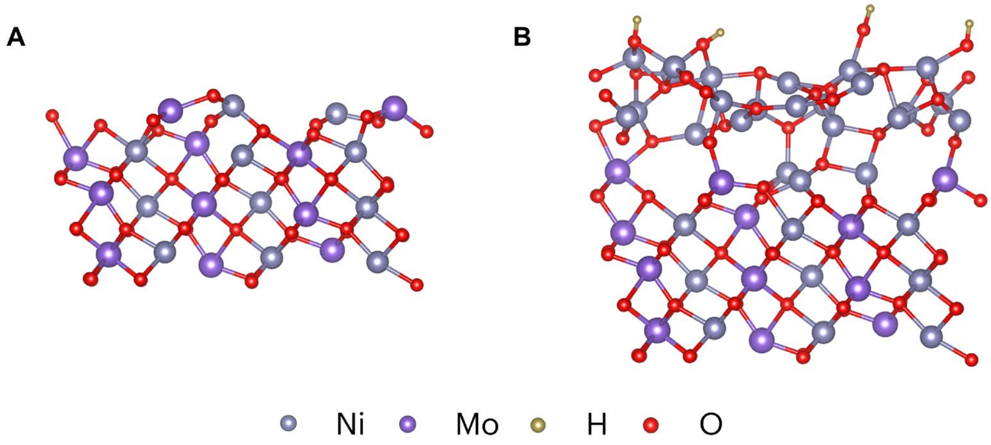

Fig. S71. Model diagram. DFT-optimized configurations of (**A**) $NiMoO_4$ (101) and (**B**) *OH-$NiO/NiMoO_4$.

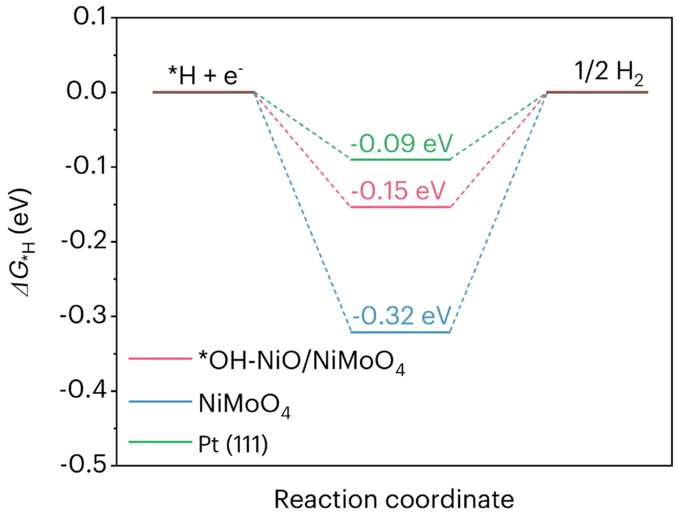

Fig. S72. DFT calculation. Gibbs free energy barrier for *H adsorption on the surface of *OH-NiO/$NiMoO_4$, $NiMoO_4$ (101) and Pt (111).

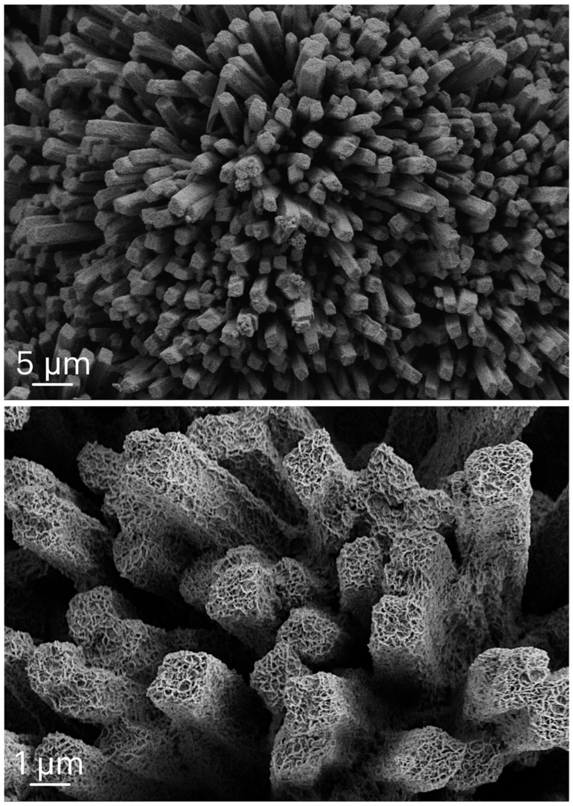

Fig. S73. Structural analysis. SEM images of the NiO/NiMoO$_x$ catalyst.

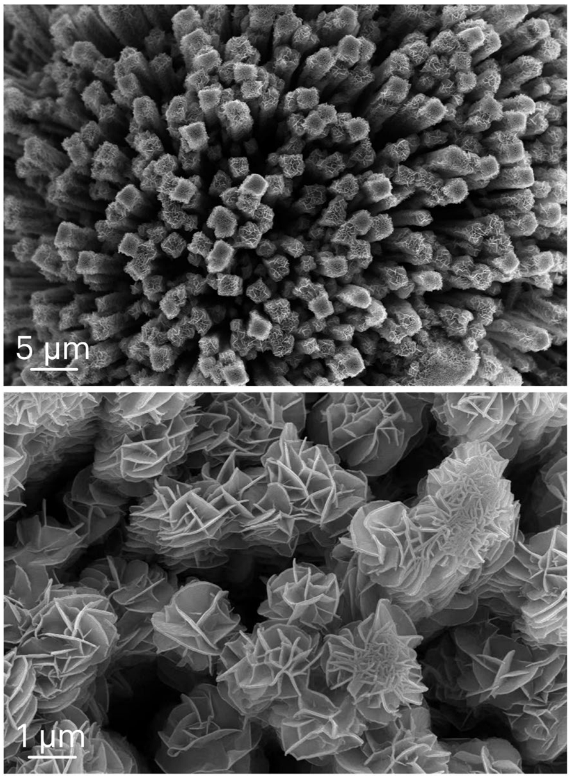

Fig. S74. Structural analysis. SEM images of the *OH-NiO/NiMoO$_x$ catalyst.

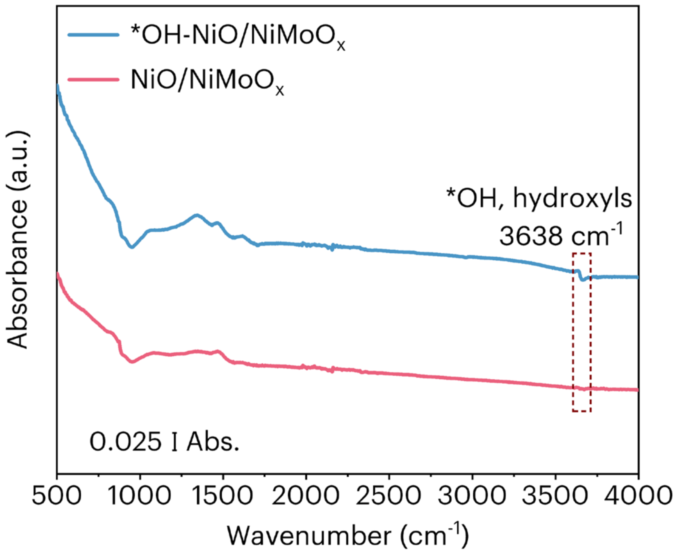

Fig. S75. Structural analysis. Fourier transform infrared (FTIR) spectra of *OH-NiO/NiMoO$_x$ and NiO/NiMoO$_x$.

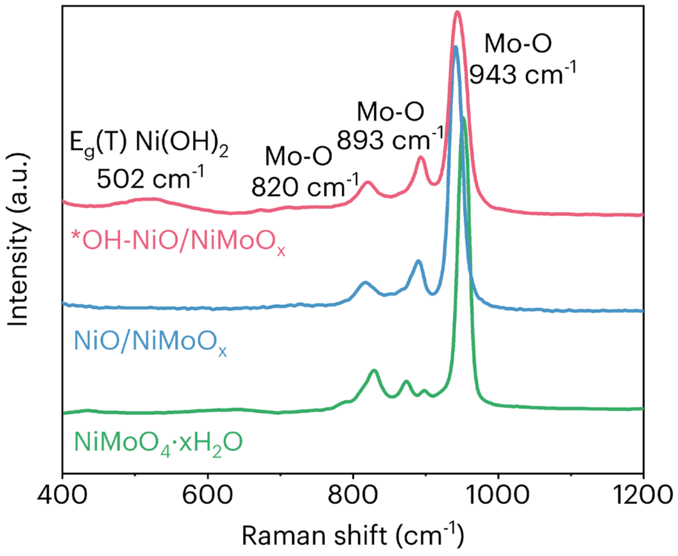

Fig. S76. Structural analysis. Raman spectra of *OH-NiO/NiMoO$_x$, NiO/NiMoO$_x$ and NiMoO$_4$·xH$_2$O.

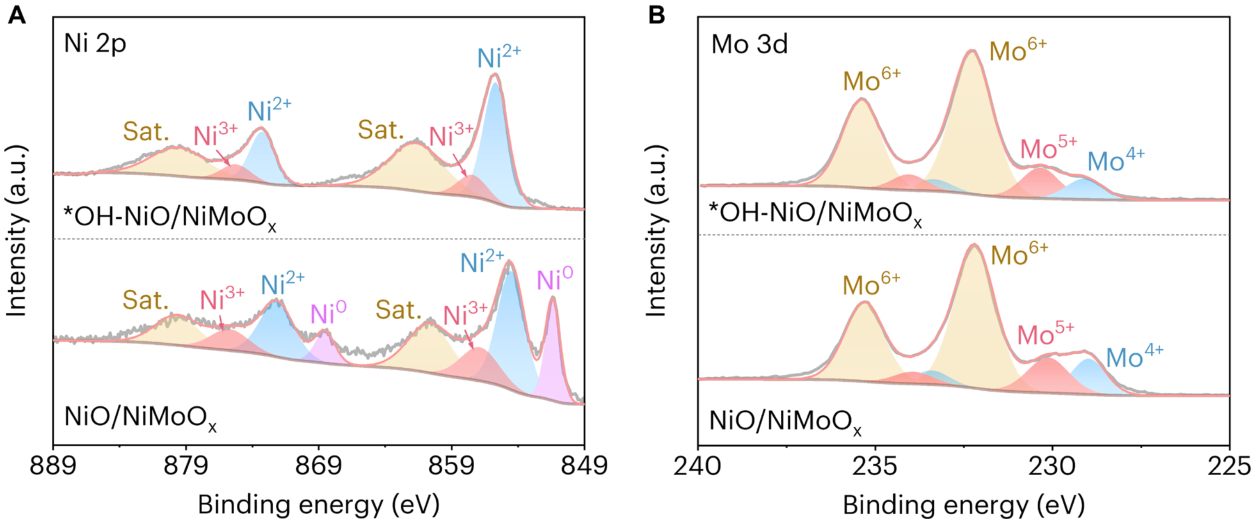

Fig. S77. XPS characterization. High-resolution (**A**) Ni 2p and (**B**) Mo 3d XPS spectra of NiO/NiMoO*x* and *OH-NiO/NiMoO*x*.

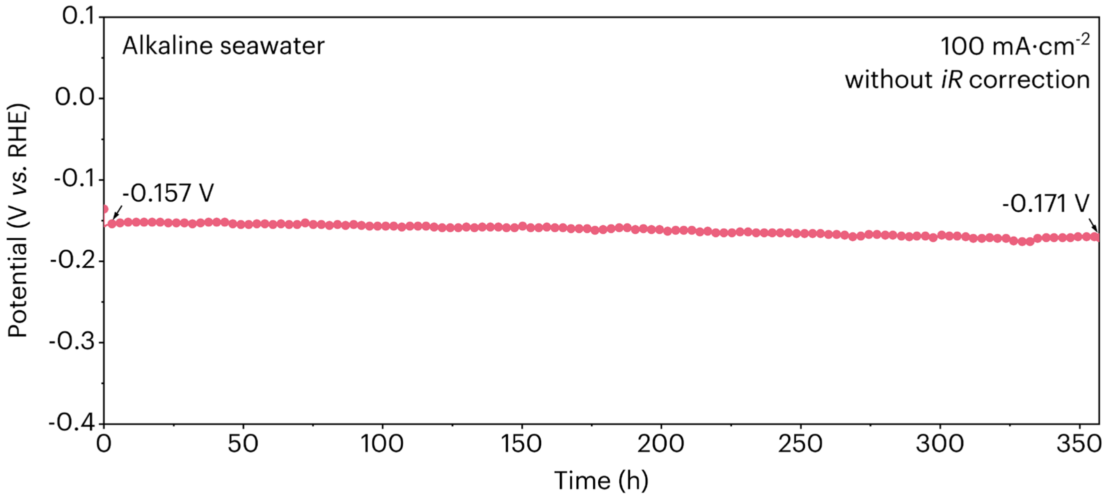

Fig. S78. HER stability analysis. Chronopotentiometry test of NiO/$NiMoO_x$ for HER in alkaline seawater under current density of 100 mA·cm^{-2}.

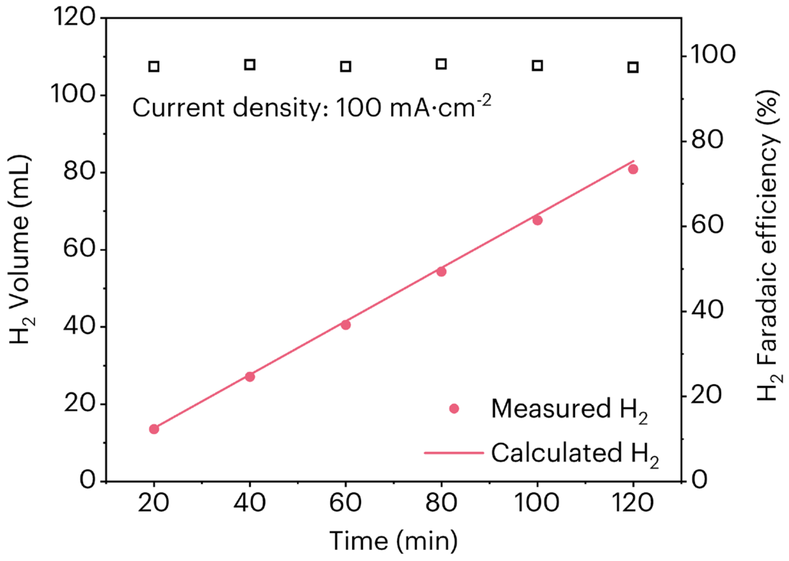

Fig. S79. Product analysis. Calculated Faradaic efficiency of NiO/NiMoO$_x$ for HER at different reaction times under current density of 100 mA·cm^{-2}.

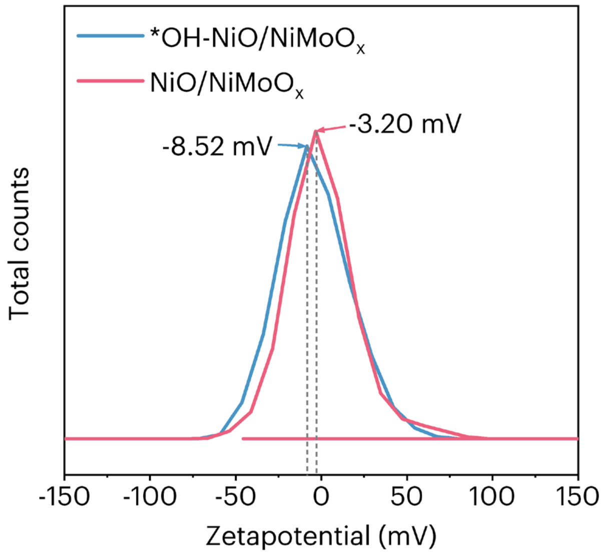

Fig. S80. Zeta potential analysis. Zeta potentials of *OH-NiO/NiMoO$_x$ and NiO/NiMoO$_x$.

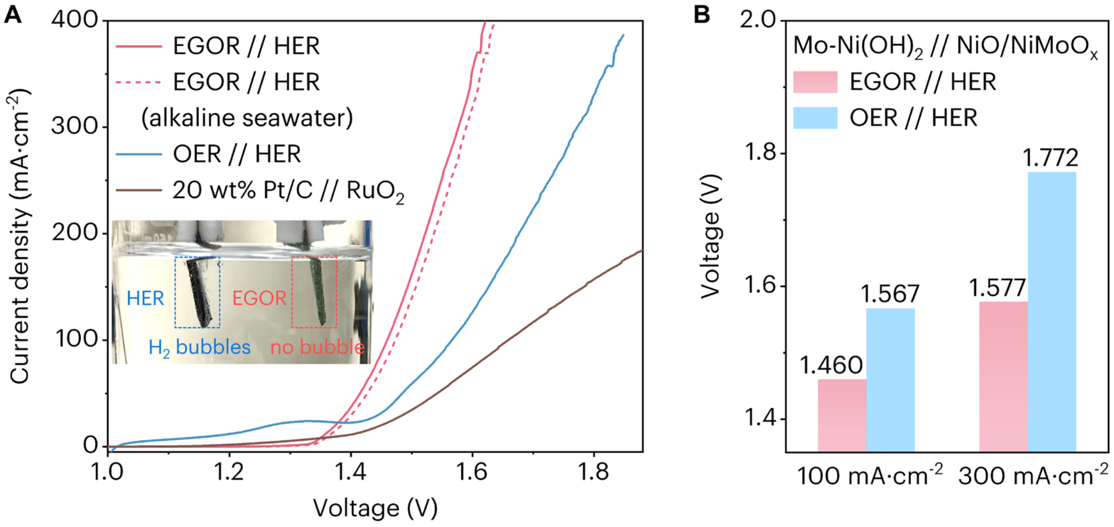

Fig. S81. Bifunctional activity. (**A**) LSV curves for EGOR // HER and OER // HER of the Mo-$Ni(OH)_2$ // NiO/$NiMoO_x$ system. Inset shows the digital image of the two-electrode system. (**B**) Comparison of cell voltages for EGOR // HER and OER // HER at the two-electrode system.

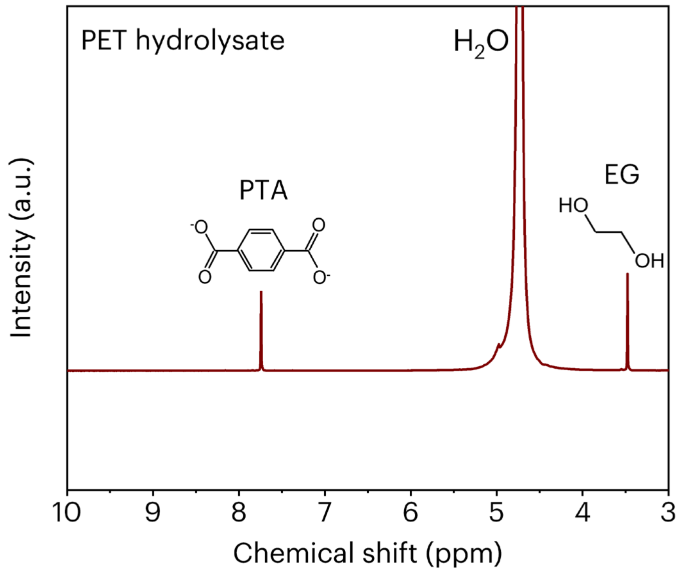

Fig. S82. ^{1}H NMR analysis. ^{1}H NMR spectra of the PET hydrolysate.

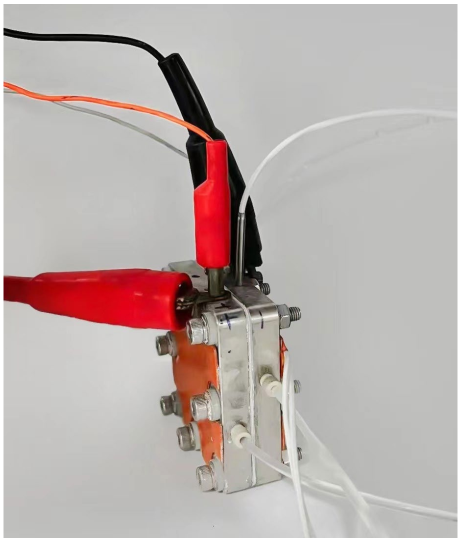

Fig. S83. Electrolyzer image. Digital image of the commercial titanium-alloy-made flow electrolyzer.

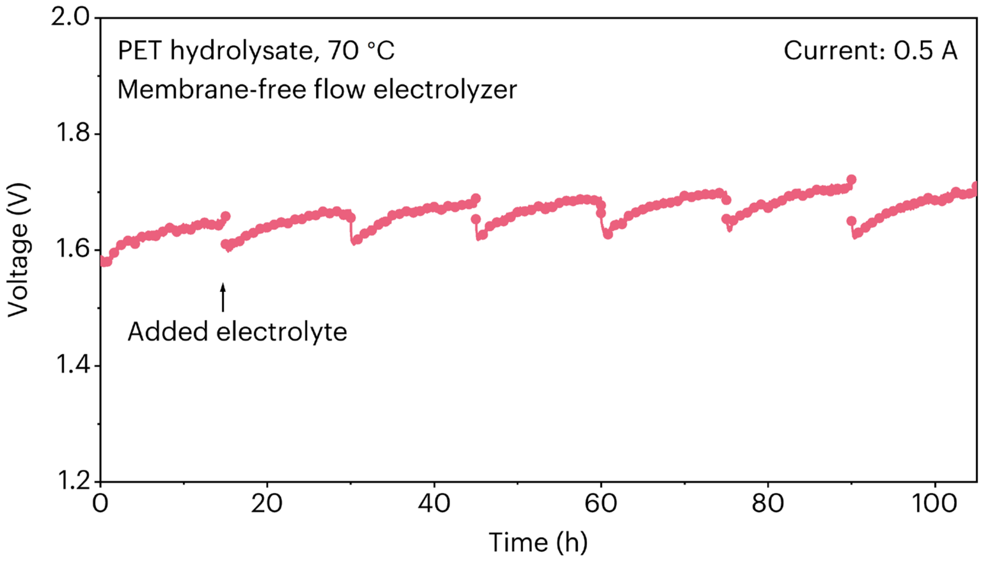

Fig. S84. Stability analysis. Chronopotentiometry test for PET hydrolysate electrolysis under current of 0.5 A at the commercial titanium-alloy-made flow electrolyzer.

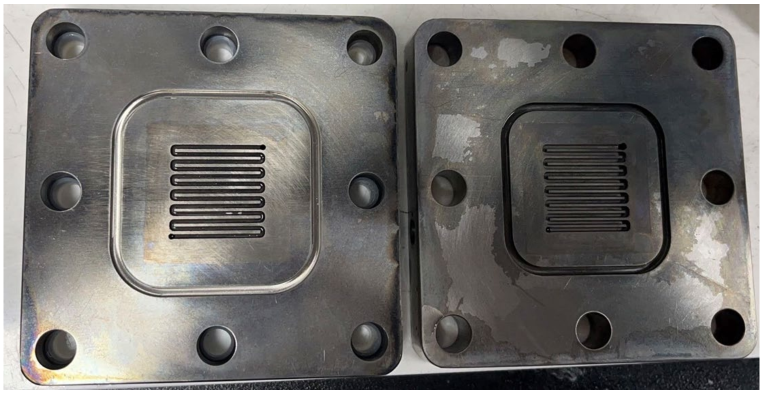

Fig. S85. Electrolyzer image. Digital image of the commercial titanium-alloy-made flow electrolyzer after PET hydrolysate electrolysis.

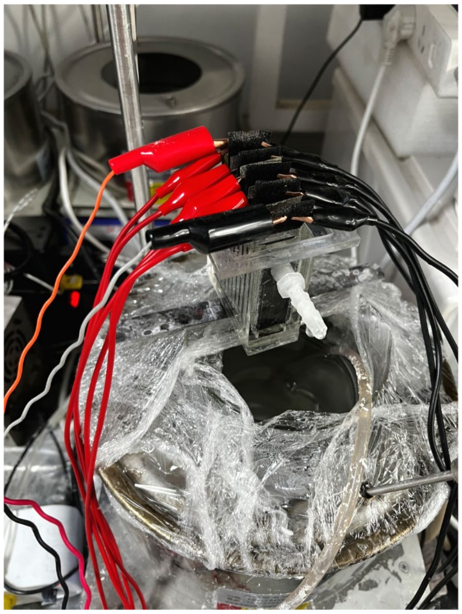

Fig. S86. Electrolyzer image. Digital image of the slot-type flow electrolyzer for PET hydrolysate electrolysis.

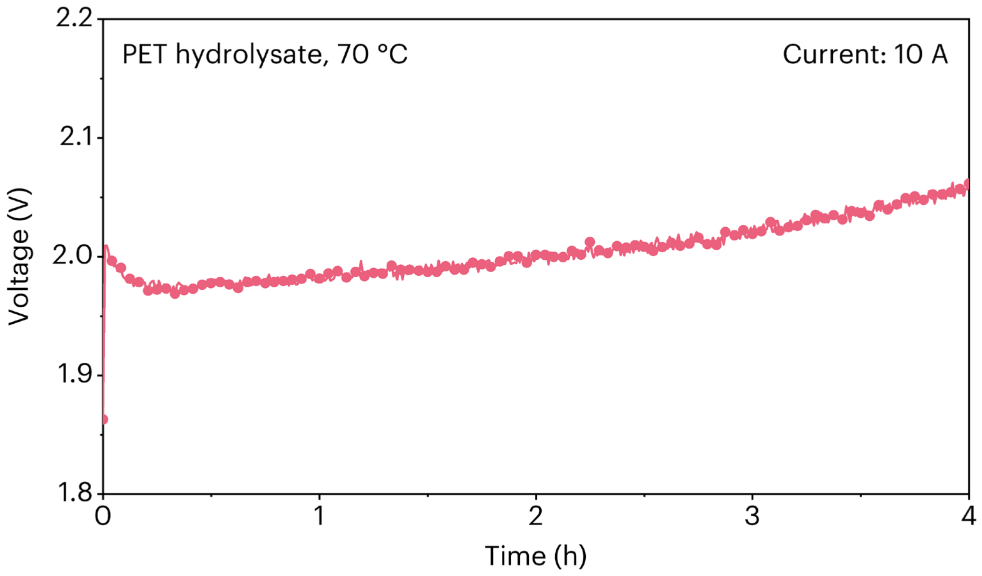

Fig. S87. Stability analysis. Chronopotentiometry test for PET hydrolysate electrolysis under current of 10 A at the five-unit parallel flow electrolyzer.

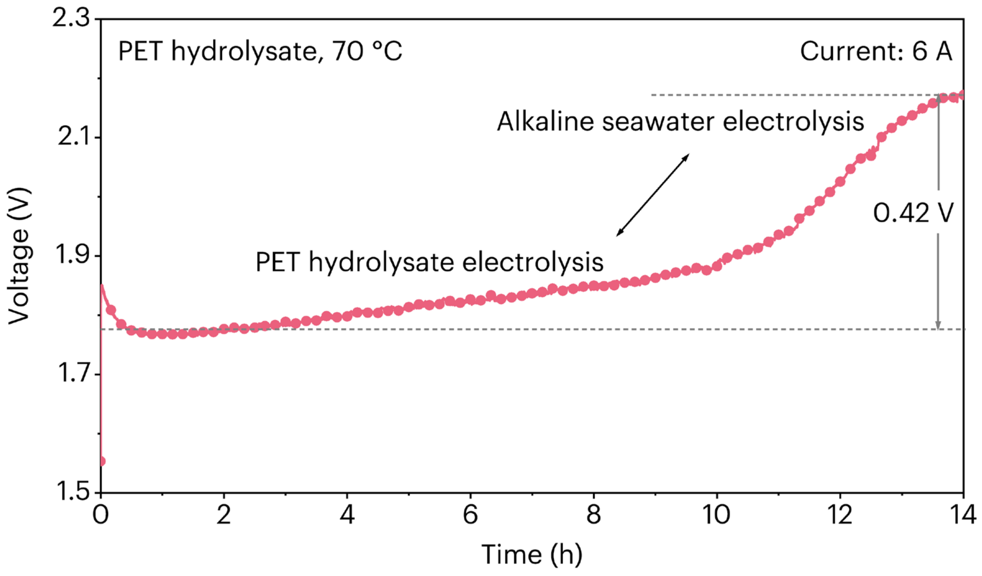

Fig. S88. Stability analysis. Chronopotentiometry test for PET hydrolysate electrolysis under current of 6 A at the five-unit parallel flow electrolyzer without replenishing electrolyte.

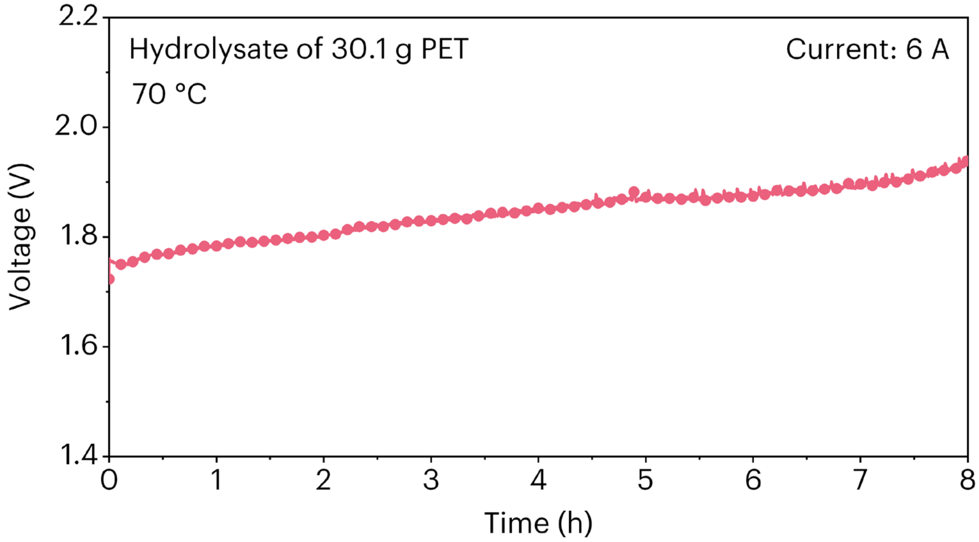

Fig. S89. Stability analysis. Chronopotentiometry test for the hydrolysate electrolysis of 30.1 g PET powders under current of 6 A at the five-unit parallel flow electrolyzer.

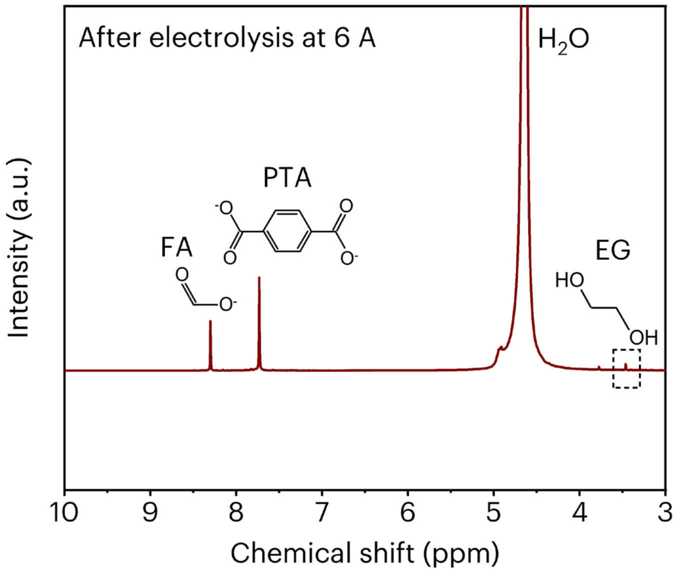

Fig. S90. ^{1}H NMR analysis. ^{1}H NMR spectra of the PET hydrolysate after electrolysis at 6 A.

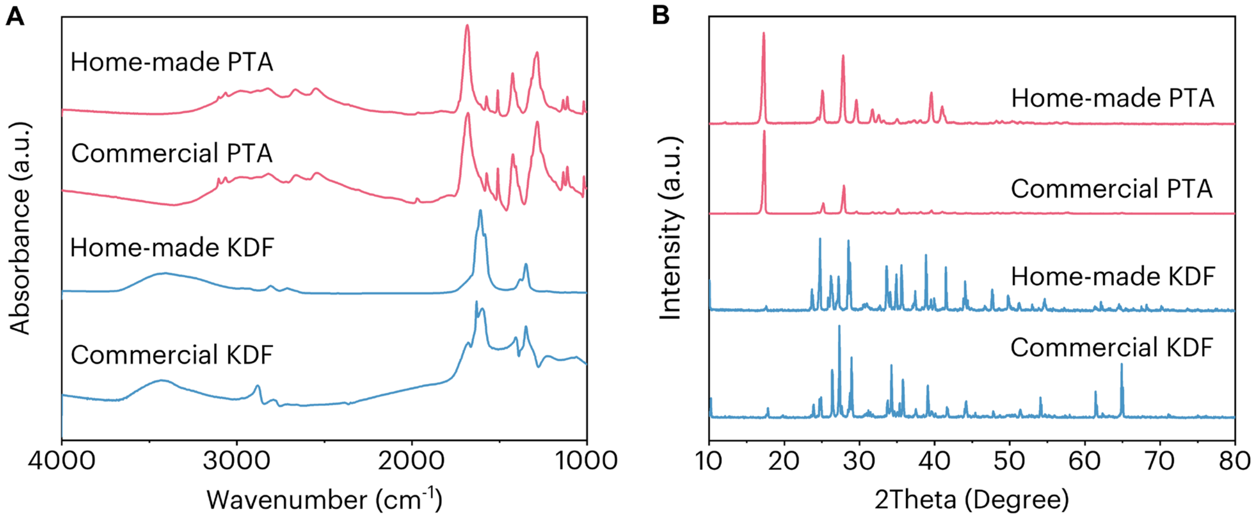

Fig. S91. Product analysis. (**A**) FTIR spectra and (**B**) XRD patterns of home-made and commercial PTA and KDF.

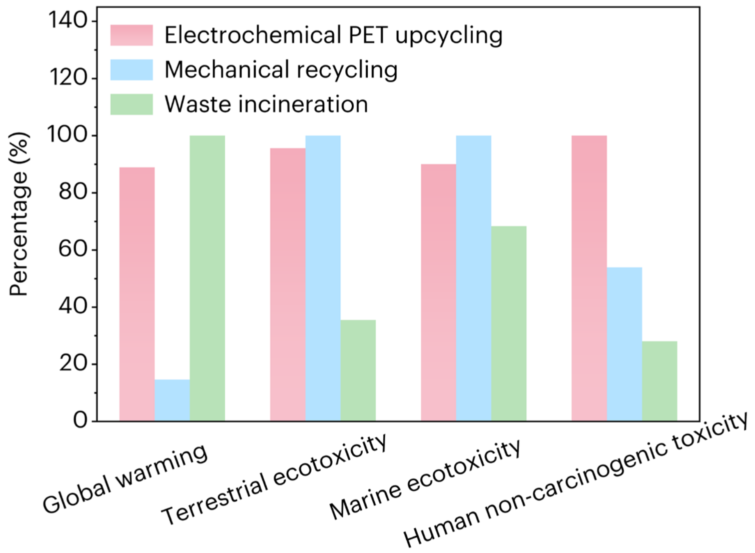

Fig. S92. LCA analysis. Life cycle assessment (LCA) analysis of electrochemical PET upcycling, mechanical recycling, waste incineration.

Table S1. Analysis of the Mo content in $NiMoO_4{\cdot}xH_2O$, Mo-NiOOH and Mo-$Ni(OH)_2$ (Before and post EGOR stability test).

Sample	Mass density ($mg{\cdot}cm^{-2}$)	Mass percent (wt%)
$NiMoO_4{\cdot}xH_2O$	7.16	35.98
Mo-NiOOH	0.35	2.46
Mo-$Ni(OH)_2$	0.19	1.34
Mo-$Ni(OH)_2$ (Post EGOR)	0.12	0.85

Table S2. EXAFS fitting parameters at the Ni K-edge (S_0^2 = 0.76).

Sample	Path	C.N.	R (Å)	$\sigma^2 \times 10^3$ (Å^2)	ΔE (eV)	R factor
Ni foil	Ni-Ni	12	2.48±0.01	0.0061	8.6±0.2	0.0012
$Ni(OH)_2$	Ni-O	1.8±0.2	1.89±0.02	0.0050	-1.9±3.3	0.0042
	Ni-O_1	1.3±0.3	2.08±0.03	0.0039		
	Ni-Ni	0.9±0.1	2.47±0.01	0.0055		
	Ni-Ni_1	1.0±0.4	3.17±0.02	0.0036		
	Ni-O_2	3.7±1.1	3.36±0.03	0.0023		
Mo-$Ni(OH)_2$	Ni-O	0.4±0.2	1.71±0.03	0.0013	-1.8±3.0	0.0069
	Ni-O_1	5.2±0.5	2.04±0.02	0.0122		
	Ni-Ni	2.3±0.2	2.48±0.01	0.0053		
	Ni-Ni_1	1.1±0.3	3.06±0.02	0.0024		
	Ni-O_2	2.0±1.3	3.44±0.05	0.0013		
NiO	Ni-O	6.3±0.5	2.05±0.01	0.0060	-3.9±1.5	0.0192
	Ni-Ni	7.3±0.8	3.10±0.01	0.0104		

Note: C.N. is the coordination number; R is the bond distance; σ^2 is the Debye-Waller factor; ΔE is the inner potential correction; R factor is the goodness of fitting data.

Table S3. Comparison of the Mo-$Ni(OH)_2$ NRs catalyst with previously reported catalysts in the alcohol oxidation performance.

Catalyst	Stability time (h)	Potential (V) at 100 $mA \cdot cm^{-2}$	Reference
Mo-$Ni(OH)_2$ NRs	306.9	1.384	This work
$NiCo_2O_4$/N-CNTG	5	1.57	*(53)*
Ni electrode	4.17	1.85	*(54)*
$NiSe_2$	10	1.62	*(55)*
$RuNiO_3$/NF	2.78	1.48	*(56)*
$NiCo_2O_4$/CFP	2	1.46	*(57)*
Co-Ni_2P/NF	10	1.4	*(58)*
N-CoO_x	15	1.53	*(59)*
OMS-Ni_1-CoP	10	1.43	*(60)*
Ni/Ni_3N_{1-x}	50	1.38	*(61)*
A-CoFeNi	35	1.38	*(62)*

Table S4. Comparison of energy-saving system driven by alcohol-oxidation with recently reported catalysts.

System	Cell voltage (V)	Current (A)	Reference
$Mo\text{-}Ni(OH)_2$ NRs	1.6	3.0	This work
	1.7	5.0	
	1.8	7.8	
Co, Cl-NiS	1.55	0.2	*(14)*
$CoNi_xP$/NF	1.76	0.5	*(13)*
Ni_1Mn_1-MOF-Se	1.84	0.4	*(15)*
Mn-CoN@C/NF	1.82	0.8	*(63)*
MnO_2-CuO/CF	1.7	0.68	*(64)*
$Co\text{-}Ni_2P$/NF	2	2.1	*(65)*
$LC\text{-}Ni(OH)_2$	2	0.5	*(66)*
$Pt_1/Ni(OH)_2$-3	1.444	1	*(1)*

Table S5. Performance comparison of different technologies for PET upcycling.

Technology	Upcycling rate ($g \cdot h^{-1}$)	Reaction temperature (°C)	Reference
Hydrolysis + Electrolysis ($Mo\text{-}Ni(OH)_2$ // $NiO/NiMoO_x$)	0.94	70	This work
Thermocatalysis (Zr-MOF MIL-140)	0.00089	260	*(67)*
Hydrogenation/Hydrogenolysis (Pd/*r*-GO and *og*-CuZn)	0.025	200	*(68)*
Methanolysis + hydrogenation (Cu/ZrO_2)	0.17	220	*(69)*
Supercritical ethanol depolymerization ($Ni\text{-}ZnO/Al_2O_3$)	6.58	262.8	*(70)*
Aminolysis-assisted hydrothermal conversion	0.21	180	*(71)*
Enzyme catalysis (FAST-PETase)	0.021	50	*(72)*
Biological-electrolysis (LCC^{ICCG}-M3 + $Pd/Ni(OH)_2$)	1.15	50	*(73)*
Photocatalysis ($CN_x|Ni_2P$)	0.00042	40	*(74)*
Photocatalysis ($Cu_1\text{-}O_4$ SACs)	0.00044	25	*(75)*
Photocatalysis ($d\text{-}NiPS_3/CdS$)	0.0011	25	(76)
Photocatalysis ($Pt\text{-}DSA/TiO_2$)	0.026	25	*(10)*

Photocatalysis ($O-CuIn_5S_8$)	0.0082	25	*(77)*
Chemoenzymatic Photoreforming (TiO_2|CotpyP)	0.00023	65	*(78)*
Photochromic-Photothermal (TiO_2-DEG)	1	190	*(79)*
Photothermal catalysis (Co SSCs)	0.17	180	*(80)*
Photoelectrocatalysis ($Ti-Fe_2O_3/Ni(OH)_x$)	0.0097	25	*(81)*
Photoelectrocatalysis ($Ni-Pi/\alpha-Fe_2O_3$)	0.00086	25	*(82)*

Table S6. Impact categories of the LCA analysis.

Impact category	PET electrochemical upcycling	PET mechanical recycling	Waste incineration
Global warming (kg CO_2-eq/kg PET)	2.97	0.49	3.34
Terrestrial ecotoxicity (kg 1,4-eq/kg PET)	12.52	13.09	4.65
Marine ecotoxicity (kg 1,4-eq/kg PET)	10.21	11.34	7.75
Human non-carcinogenic toxicity (kg 1,4-eq/kg PET)	9.57	5.16	2.69

REFERENCES AND NOTES

1. M. Song, Y. Wu, Z. Zhao, M. Zheng, C. Wang, J. Lu, Corrosion engineering of part-per-million single atom $Pt_1/Ni(OH)_2$ electrocatalyst for PET upcycling at ampere-level current density. *Adv. Mater.* **36**, e2403234 (2024).

2. Plastic upcycling. *Nat. Catal.* **2**, 945–946 (2019).

3. M. Macleod, H. P. H. Arp, M. B. Tekman, A. Jahnke, The global threat from plastic pollution. *Science* **373**, 61–65 (2021).

4. C. Jehanno, J. W. Alty, M. Roosen, S. De Meester, A. P. Dove, E. Y. X. Chen, F. A. Leibfarth, H. Sardon, Critical advances and future opportunities in upcycling commodity polymers. *Nature* **603**, 803–814 (2022).

5. Z. Gao, B. Ma, S. Chen, J. Tian, C. Zhao, Converting waste PET plastics into automobile fuels and antifreeze components. *Nat. Commun.* **13**, 3343 (2022).

6. M. Wang, Y. Gao, S. Yuan, J. Deng, J. Yang, J. Yan, S. Yu, B. Xu, D. Ma, Complete hydrogenolysis of mixed plastic wastes. *Nat. Chem. Eng.* **1**, 376–384 (2024).

7. Y. Yan, H. Zhou, S.-M. Xu, J. Yang, P. Hao, X. Cai, Y. Ren, M. Xu, X. Kong, M. Shao, Z. Li, H. Duan, Electrocatalytic upcycling of biomass and plastic wastes to biodegradable polymer monomers and hydrogen fuel at high current densities. *J. Am. Chem. Soc.* **145**, 6144–6155 (2023).

8. M. Du, Y. Zhang, S. Kang, C. Xu, Y. Ma, L. Cai, Y. Zhu, Y. Chai, B. Qiu, Electrochemical production of glycolate fuelled by polyethylene terephthalate plastics with improved techno-economics. *Small* **19**, e2303693 (2023).

9. H. Yue, Y. Zhao, X. Ma, J. Gong, Ethylene glycol: Properties, synthesis, and applications. *Chem. Soc. Rev.* **41**, 4218–4244 (2012).

10. C. W. Lee, B.-H. Lee, S. Park, Y. Jung, J. Han, J. Heo, K. Lee, W. Ko, S. Yoo, M. S. Bootharaju, J. Ryu, K. T. Nam, M. Kim, T. Hyeon, Photochemical tuning of dynamic defects for high-performance atomically dispersed catalysts. *Nat. Mater.* **23**, 552–559 (2024).

11. Z. Guo, H. Zhang, H. Chen, M. Zhang, X. Tang, M. Wang, D. Ma, Hydrogenating polyethylene terephthalate into degradable polyesters. *Angew. Chem. Int. Ed. Engl.* **64**, e202418157 (2025).

12. X. Liu, Z. Fang, D. Xiong, S. Gong, Y. Niu, W. Chen, Z. Chen, Upcycling PET in parallel with energy-saving H_2 production via bifunctional nickel-cobalt nitride nanosheets. *Nano Res.* **16**, 4625–4633 (2023).

13. H. Zhou, Y. Ren, Z. Li, M. Xu, Y. Wang, R. Ge, X. Kong, L. Zheng, H. Duan, Electrocatalytic upcycling of polyethylene terephthalate to commodity chemicals and H_2 fuel. *Nat. Commun.* **12**, 4679 (2021).

14. Z. Chen, R. Zheng, T. Bao, T. Ma, W. Wei, Y. Shen, B.-J. Ni, Dual-doped nickel sulfide for electro-upgrading polyethylene terephthalate into valuable chemicals and hydrogen fuel. *Nano Micro Lett.* **15**, 210 (2023).

15. J. Qi, Y. Du, Q. Yang, N. Jiang, J. Li, Y. Ma, Y. Ma, X. Zhao, J. Qiu, Energy-saving and product-oriented hydrogen peroxide electrosynthesis enabledby electrochemistry pairing and product engineering. *Nat. Commun.* **14**, 6263 (2023).

16. Y. Zhang, C. Li, F. H. S. Chiew, D. A. Post, X. Zhang, N. Ma, J. Tian, D. Kong, L. R. Leung, Q. Yu, J. Shi, C. Liu, Southern Hemisphere dominates recent decline in global water availability. *Science* **382**, 579–584 (2023).

17. China Meteorological Administration Wind and Solar Energy Centre. Annual report on wind and solar energy resources in China (2024).

18. L. Zhang, Z. Wang, J. Qiu, Energy-saving hydrogen production by seawater electrolysis coupling sulfion degradation. *Adv. Mater.* **34**, 2109321 (2022).

19. Y. Li, L. Q. Lee, H. Zhao, Y. Zhao, P. Gao, H. Li, Alcohol-alkali hydrolysis for high-throughput PET waste electroreforming-assisted green hydrogen generation. *J. Mater. Chem. A* **12**, 2121–2128 (2024).

20. Y. Mao, S. Fan, X. Li, J. Shi, M. Wang, Z. Niu, G. Chen, Trash to treasure: Electrocatalytic upcycling of polyethylene terephthalate (PET) microplastic to value-added products by $Mn_{0.1}Ni_{0.9}Co_2O_{4-\delta}$ RSFs spinel. *J. Hazardous Mater.* **457**, 131743 (2023).

21. M. Yuan, J. Chen, Y. Xu, R. Liu, T. Zhao, J. Zhang, Z. Ren, Z. Liu, C. Streb, H. He, C. Yang, S. Zhang, G. Zhang, Highly selective electroreduction of N_2 and CO_2 to urea over artificial frustrated Lewis pairs. *Energy Environ. Sci.* **14**, 6605–6615 (2021).

22. L. Wang, G. Kehr, C. G. Daniliuc, M. Brinkkötter, T. Wiegand, A.-L. Wübker, H. Eckert, L. Liu, J. G. Brandenburg, S. Grimme, G. Erker, Solid state frustrated Lewis pair chemistry. *Chem. Sci.* **9**, 4859–4865 (2018).

23. Y. Huang, M. Li, F. Pan, Z. Zhu, H. Sun, Y. Tang, G. Fu, Plasma-induced Mo-doped Co_3O_4 with enriched oxygen vacancies for electrocatalytic oxygen evolution in water splitting. *Carbon Energy* **5**, e279 (2023).

24. J. Hu, S. Li, Y. Li, J. Wang, Y. Du, Z. Li, X. Han, J. Sun, P. Xu, A crystalline-amorphous Ni-$Ni(OH)_2$ core-shell catalyst for the alkaline hydrogen evolution reaction. *J. Mater. Chem. A* **8**, 23323–23329 (2020).

25. Z. Xiao, Y. Qian, T. Tan, H. Lu, C. Liu, B. Wang, Q. Zhang, M. T. Sarwar, R. Gao, A. Tang, H. Yang, Energy-saving hydrogen production by water splitting coupling urea decomposition and oxidation reactions. *J. Mater. Chem. A* **11**, 259–267 (2022).

26. Y.-Y. Chen, Y. Zhang, X. Zhang, T. Tang, H. Luo, S. Niu, Z.-H. Dai, L.-J. Wan, J.-S. Hu, Self-templated fabrication of $MoNi_4/MoO_{3-x}$ nanorod arrays with dual active components for highly efficient hydrogen evolution. *Adv. Mater.* **29**, 1703311 (2017).

27. D. Wang, C. Han, Z. Xing, Q. Li, X. Yang, Pt-like catalytic behavior of MoNi decorated $CoMoO_3$ cuboid arrays for the hydrogen evolution reaction. *J. Mater. Chem. A* **6**, 15558–15563 (2018).

28. D. Jia, H. Gao, L. Xing, X. Chen, W. Dong, X. Huang, G. Wang, 3D self-supported porous NiO@$NiMoO_4$ core-shell nanosheets for highly efficient oxygen evolution reaction. *Inorg. Chem.* **58**, 6758–6764 (2019).

29. Y. Zhao, B. Zhao, J. Liu, G. Chen, R. Gao, S. Yao, M. Li, Q. Zhang, L. Gu, J. Xie, X. Wen, L.-Z. Wu, C.-H. Tung, D. Ma, T. Zhang, Oxide-modified nickel photocatalysts for the production of hydrocarbons in visible light. *Angew. Chem. Int. Ed. Engl.* **55**, 4215–4219 (2016).

30. M. Fleischmann, K. Korinek, D. Pletcher, The oxidation of organic compounds at a nickel anode in alkaline solution. *J. Electroanal. Chem. Interfacial Electrochem.* **31**, 39–49 (1971).

31. J. M. Mayer, Bonds over electrons: Proton coupled electron transfer at solid-solution interfaces. *J. Am. Chem. Soc.* **145**, 7050–7064 (2023).

32. S.-K. Geng, Y. Zheng, S.-Q. Li, H. Su, X. Zhao, J. Hu, H.-B. Shu, M. Jaroniec, P. Chen, Q.-H. Liu, S.-Z. Qiao, Nickel ferrocyanide as a high-performance urea oxidation electrocatalyst. *Nat. Energy* **6**, 904–912 (2021).

33. Y. Qi, Y. Zhang, L. Yang, Y. Zhao, Y. Zhu, H. Jiang, C. Li, Insights into the activity of nickel boride/nickel heterostructures for efficient methanol electrooxidation. *Nat. Commun.* **13**, 4602 (2022).

34. L. Fan, Y. Ji, G. Wang, J. Chen, K. Chen, X. Liu, Z. Wen, High entropy alloy electrocatalytic electrode toward alkaline glycerol valorization coupling with acidic hydrogen production. *J. Am. Chem. Soc.* **144**, 7224–7235 (2022).

35. F. Liu, X. Gao, R. Shi, Z. Guo, E. C. M. Tse, Y. Chen, Concerted and selective electrooxidation of polyethylene-terephthalate-derived alcohol to glycolic acid at an industry-

level current density over a Pd-Ni(OH)$_2$ catalyst. *Angew. Chem. Int. Ed. Engl.* **62**, e202300094 (2023).

36. Y. Wang, H. Zhou, H. Sun, X. Zhang, X. Dai, C. Qin, H. Zhao, J. Li, M. Wang, J.-Y. Ye, S.-G. Sun, Implanting Mo atoms into surface lattice of Pt$_3$Mn alloys enclosed by high-indexed facets: Promoting highly active sites for ethylene glycol oxidation. *ACS Catal.* **9**, 442–455 (2019).

37. J. Schnaidt, M. Heinen, Z. Jusys, R. J. Behm, Electro-oxidation of ethylene glycol on a Pt-film electrode studied by combined in situ infrared spectroscopy and online mass spectrometry. *J. Phys. Chem. C* **116**, 2872–2883 (2012).

38. L. Demarconnay, S. Brimaud, C. Coutanceau, J. M. Léger, Ethylene glycol electrooxidation in alkaline medium at multi-metallic Pt based catalysts. *J. Electroanal. Chem.* **601**, 169–180 (2007).

39. L. W. H. Leung, M. J. Weaver, Real-time FTIR spectroscopy as a quantitative kinetic probe of competing electrooxidation pathways of small organic molecules. *J. Phys. Chem.* **92**, 4019–4022 (1988).

40. R. Nelson, C. Ertural, J. George, V. L. Deringer, G. Hautier, R. Dronskowski, LOBSTER: Local orbital projections, atomic charges, and chemical-bonding analysis from projector-augmented-wave-based density-functional theory. *J. Comput. Chem.* **41**, 1931–1940 (2020).

41. Y. Luo, Z. Zhang, M. Chhowalla, B. Liu, Recent advances in design of electrocatalysts for high-current-density water splitting. *Adv. Mater.* **34**, e2108133 (2022).

42. Z. Jia, R. Ding, W. Yu, Y. Li, A. Wang, M. Liu, F. Yang, X. Sun, E. Liu, Unraveling the charge storage and activity-enhancing mechanisms of Zn-doping perovskite fluorides and engineering the electrodes and electrolytes for wide-temperature aqueous supercabatteries. *Adv. Funct. Mater.* **32**, 2107674 (2022).

43. C. Li, J.-Y. Xue, W. Zhang, F.-L. Li, H. Gu, P. Braunstein, J.-P. Lang, Accelerating water dissociation at carbon supported nanoscale Ni/NiO heterojunction electrocatalysts for high-efficiency alkaline hydrogen evolution. *Nano Res.* **16**, 4742–4750 (2023).

44. G. Huyghebaert, R. Ducatelle, F. V. Immerseel, An update on alternatives to antimicrobial growth promoters for broilers. *Vet. J.* **187**, 182–188 (2011).

45. Y. Peng, J. Yang, C. Deng, J. Deng, L. Shen, Y. Fu, Acetolysis of waste polyethylene terephthalate for upcycling and life-cycle assessment study. *Nat. Commun.* **14**, 3249 (2023).

46. G. Kresse, J. Furthmüller, Efficient iterative schemes for ab initio total-energy calculations using a plane-wave basis set. *Phys. Rev. B* **54**, 11169–11186 (1996).

47. G. Kresse, D. Joubert, From ultrasoft pseudopotentials to the projector augmented-wave method. *Phys. Rev. B* **59**, 1758–1775 (1999).

48. J. P. Perdew, K. Burke, M. Ernzerhof, Generalized gradient approximation made simple. *Phys. Rev. Lett.* **77**, 3865–3868 (1996).

49. S. Grimme, J. Antony, S. Ehrlich, H. Krieg, A consistent and accurate ab initio parametrization of density functional dispersion correction (DFT-D) for the 94 elements H-Pu. *J. Chem. Phys.* **132**, 154104 (2010).

50. Y. Jin, S. Huang, X. Yue, H. Du, P. K. Shen, Mo- and Fe-modified $Ni(OH)_2$/NiOOH nanosheets as highly active and stable electrocatalysts for oxygen evolution reaction. *ACS Catal.* **8**, 2359–2363 (2018).

51. H. Shin, K. U. Hansen, F. Jiao, Techno-economic assessment of low-temperature carbon dioxide electrolysis. *Nat. Sustain.* **4**, 911–919 (2021).

52. T. Zhang, Y.-H. Yu, C.-W. Liu, G.-W. Qin, S. Li, Constructing Ni_4W/WO_3/NF with strongly coupled interface for hydrogen evolution in alkaline media. *Rare Met.* **42**, 3945–3951 (2023).

53. A. Suárez-Barajas, C. M. Ramos-Castillo, A. Olivas, M. Guerra-Balcázar, L. Álvarez-Contreras, N. Arjona, Oxygen vacancy-enriched $NiCo_2O_4$ spinels/N-doped carbon nanotubes-graphene composites for the ethylene glycol electro-oxidation. *Fuel* **360**, 130371 (2024).

54. X.-Y. Ma, H.-Z. Ma, S.-H. He, Y. Zhang, Y.-N. Yi, Y.-Y. Yang, The electrocatalytic activity and selectivity of ethylene glycol oxidation into value-added chemicals at iron-group electrodes in alkaline media. *Mater. Today Phys.* **37**, 101191 (2023).

55. J. Li, L. Li, X. Ma, X. Han, C. Xing, X. Qi, R. He, J. Arbiol, H. Pan, J. Zhao, J. Deng, Y. Zhang, Y. Yang, A. Cabot, Selective ethylene glycol oxidation to formate on nickel selenide with simultaneous evolution of hydrogen. *Adv. Sci.* **10**, 2300841 (2023).

56. A. G. Prince, L. Durai, S. Badhulika, Solid state synthesis of a $RuNiO_3$ perovskite nanomaterial as an electro-catalyst for direct alcohol (ethanol, methanol and ethylene glycol) fuel cell applications. *New J. Chem.* **47**, 3870–3879 (2023).

57. J. Wang, X. Li, M. Wang, T. Zhang, X. Chai, J. Lu, T. Wang, Y. Zhao, D. Ma, Electrocatalytic valorization of poly(ethylene terephthalate) plastic and CO_2 for simultaneous production of formic acid. *ACS Catal.* **12**, 6722–6728 (2022).

58. Y. Li, L. Q. Lee, Z. G. Yu, H. Zhao, Y.-W. Zhang, P. Gao, H. Li, Coupling of PET waste electroreforming with green hydrogen generation using bifunctional catalyst. *Sustain. Energy Fuels* **6**, 4916–4924 (2022).

59. Z. Ke, N. Williams, X. Yan, S. Younan, D. He, X. Song, X. Pan, X. Xiao, J. Gu, Solar-assisted co-electrolysis of glycerol and water for concurrent production of formic acid and hydrogen. *J. Mater. Chem. A* **9**, 19975–19983 (2021).

60. N. Wang, X. Li, M.-K. Hu, W. Wei, S.-H. Zhou, X.-T. Wu, Q.-L. Zhu, Ordered macroporous superstructure of bifunctional cobalt phosphide with heteroatomic modification for paired hydrogen production and polyethylene terephthalate plastic recycling. *Appl. Catal. B* **316**, 121667 (2022).

61. C. Zhou, C. Jia, X. Xiang, L. Wang, S. Wu, N. Zhang, S. Zhao, G. Yang, Y. Chen, Self-supported $Ni/Ni_3N_{1-}x$ heterostructures with abundant nitrogen vacancies as efficient electrocatalysts for ethylene glycol oxidation. *J. Mater. Chem. A* **12**, 15772–15780 (2024).

62. Z.-H. Zhang, Z.-R. Yu, Y. Zhang, A. Barras, A. Addad, P. Roussel, L.-C. Tang, S. Szunerits, R. Boukherroub, Seawater corrosive engineering assisted in-situ room temperature synthesis of Ni/Co/Fe trimetallic composition to achieve polyester plastics upgrading and green hydrogen production. *Chem. Eng. J.* **498**, 155472 (2024).

63. J. Li, X. Meng, X. Song, J. Qi, F. Liu, X. Xiao, Y. Du, G. Xu, Z. Jiang, S. Ye, S. Huang, J. Qiu, Valence engineering via manganese-doping on cobalt nitride nanoarrays for efficient electrochemically paired glycerol valorization and H_2 production. *Adv. Funct. Mater.* **34**, 2316718 (2024).

64. Z. Huang, H. Ren, J. Guo, Y. Tang, D. Ye, J. Zhang, H. Zhao, High DHA selectivity and low-cost electrode for glycerol oxidation: CuO regulates MnO_2 electron density to promote DHA desorption. *Appl Catal B* **351**, 123986 (2024).

65. L. Ma, Y. Miao, J. Yang, Y. Fu, Y. Yan, Z. Zhang, Z. Li, M. Shao, Promoting electrocatalytic glycerol C─C bond cleavage to formate coupled with H_2 production over a $Cu_xNi_{2-}xP$ catalyst. *Adv. Energy Mater.* **14**, 2401061 (2024).

66. G. Fu, X. Kang, Y. Zhang, X. Yang, L. Wang, X.-Z. Fu, J. Zhang, J.-L. Luo, J. Liu, Coordination effect-promoted durable $Ni(OH)_2$ for energy-saving hydrogen evolution from water/methanol co-electrocatalysis. *Nano Micro Lett.* **14**, 200 (2022).

67. Y. Wu, X. Wang, K. O. Kirlikovali, X. Gong, A. Atilgan, K. Ma, N. M. Schweitzer, N. C. Gianneschi, Z. Li, X. Zhang, O. K. Farha, Catalytic degradation of polyethylene terephthalate using a phase-transitional zirconium-based metal-organic framework. *Angew. Chem. Int. Ed. Engl.* **61**, e202117528 (2022).

68. Z. Sun, K. Wang, Q. Lin, W. Guo, M. Chen, C. Chen, C. Zhang, J. Fei, Y. Zhu, J. Li, Y. Liu, H. He, Y. Cao, Value-added upcycling of PET to 1,4-cyclohexanedimethanol by a

hydrogenation/hydrogenolysis relay catalysis. *Angew. Chem. Int. Ed. Engl.* **63**, e202408561 (2024).

69. J. Cheng, J. Xie, Y. Xi, X. Wu, R. Zhang, Z. Mao, H. Yang, Z. Li, C. Li, Selective upcycling of polyethylene terephthalate towards high-valued oxygenated chemical methyl p-methyl benzoate using a Cu/ZrO_2 catalyst. *Angew. Chem. Int. Ed. Engl.* **63**, e202319896 (2024).

70. P. Wang, J. Xiao, R. Liu, X. Qiang, Z. Duan, C. Liang, A hybrid classification and evaluation method based on deep learning for decoration and renovation waste in view of recycling. *Waste Manag.* **191**, 1–12 (2025).

71. K. Chan, A. Zinchenko, Aminolysis-assisted hydrothermal conversion of waste PET plastic to N-doped carbon dots with markedly enhanced fluorescence. *J. Environ. Chem. Eng.* **10**, 107749 (2022).

72. H. Lu, D. J. Diaz, N. J. Czarnecki, C. Zhu, W. Kim, R. Shroff, D. J. Acosta, B. R. Alexander, H. O. Cole, Y. Zhang, N. A. Lynd, A. D. Ellington, H. S. Alper, Machine learning-aided engineering of hydrolases for PET depolymerization. *Nature* **604**, 662–667 (2022).

73. M. Du, R. Xue, W. Yuan, Y. Cheng, Z. Cui, W. Dong, B. Qiu, Tandem integration of biological and electrochemical catalysis for efficient polyester upcycling under ambient conditions. *Nano Lett.* **24**, 9768–9775 (2024).

74. T. Uekert, H. Kasap, E. Reisner, Photoreforming of nonrecyclable plastic waste over a carbon nitride/nickel phosphide catalyst. *J. Am. Chem. Soc.* **141**, 15201–15210 (2019).

75. Z. Li, Y. Yang, C. Zhang, W. Fan, G. Li, J. Fang, L. Lu, Atomically engineering the metal-support interaction of single-atom Cu/TiO_2 for efficient polyethylene terephthalate plastic photoreforming. *Chem Catal.* **4**, 100902 (2024).

76. S. Zhang, H. Li, L. Wang, J. Liu, G. Liang, K. Davey, J. Ran, S.-Z. Qiao, Boosted photoreforming of plastic waste via defect-rich $NiPS_3$ nanosheets. *J. Am. Chem. Soc.* **145**, 6410–6419 (2023).

77. M. Du, M. Xing, W. Yuan, L. Zhang, T. Sun, T. Sheng, C. Zhou, B. Qiu, Upgrading polyethylene terephthalate plastic into commodity chemicals paired with hydrogen evolution over a partially oxidized $CuIn_5S_8$ nanosheet photocatalyst. *Green Chem.* **25**, 9818–9825 (2023).

78. S. Bhattacharjee, C. Guo, E. Lam, J. M. Holstein, M. Rangel Pereira, C. M. Pichler, C. Pornrungroj, M. Rahaman, T. Uekert, F. Hollfelder, E. Reisner, Chemoenzymatic photoreforming: A sustainable approach for solar fuel generation from plastic feedstocks. *J. Am. Chem. Soc.* **145**, 20355–20364 (2023).

79. Y. Liu, C. Zhang, J. Feng, X. Wang, Z. Ding, L. He, Q. Zhang, J. Chen, Y. Yin, Integrated photochromic-photothermal processes for catalytic plastic upcycling. *Angew. Chem. Int. Ed. Engl.* **62**, e202308930 (2023).

80. Y. Liu, X. Wang, Q. Li, T. Yan, X. Lou, C. Zhang, M. Cao, L. Zhang, T.-K. Sham, Q. Zhang, L. He, J. Chen, Photothermal catalytic polyester upcycling over cobalt single-site catalyst. *Adv. Funct. Mater.* **33**, 2210283 (2023).

81. X. Li, J. Wang, M. Sun, X. Qian, Y. Zhao, Ti-Fe_2O_3/$Ni(OH)_x$ as an efficient and durable photoanode for the photoelectrochemical catalysis of PET plastic to formic acid. *J. Energy Chem.* **78**, 487–496 (2023).

82. B. Zhang, H. Zhang, Y. Pan, J. Shao, X. Wang, Y. Jiang, X. Xu, S. Chu, Photoelectrochemical conversion of plastic waste into high-value chemicals coupling hydrogen production. *Chem. Eng. J.* **462**, 142247 (2023).
